# Genotoxic stress inhibits Ewing sarcoma cell growth by modulating alternative pre-mRNA processing of the RNA helicase *DHX9*

**DOI:** 10.18632/oncotarget.5033

**Published:** 2015-10-02

**Authors:** Marco Fidaleo, Francesca Svetoni, Elisabetta Volpe, Belén Miñana, Daniela Caporossi, Maria Paola Paronetto

**Affiliations:** ^1^ Department of Movement, Human and Health Sciences, University of Rome “Foro Italico”, Rome, Italy; ^2^ Laboratories of Cellular and Molecular Neurobiology and of Neuroimmunology, CERC, Fondazione Santa Lucia, Rome, Italy; ^3^ Centre de Regulació Genòmica, Barcelona, Spain; ^4^ Universitat Pompeu Fabra, Barcelona, Spain

**Keywords:** Ewing sarcoma, alternative splicing, DNA damage, *DHX9*

## Abstract

Alternative splicing plays a key role in the DNA damage response and in cancer. Ewing Sarcomas (ES) are aggressive tumors caused by different chromosomal translocations that yield in-frame fusion proteins driving transformation. RNA profiling reveals genes differentially regulated by UV light irradiation in two ES cell lines exhibiting different sensitivity to genotoxic stress. In particular, irradiation induces a new isoform of the RNA helicase *DHX9* in the more sensitive SK-N-MC cells, which is targeted to nonsense-mediated decay (NMD), causing its downregulation. DHX9 protein forms a complex with RNA polymerase II (RNAPII) and EWS-FLI1 to enhance transcription. Silencing of *DHX9* in ES cells sensitizes them to UV treatment and impairs recruitment of EWS-FLI1 to target genes, whereas DHX9 overexpression protects ES cells from genotoxic stress. Mechanistically, we found that UV light irradiation leads to enhanced phosphorylation and decreased processivity of RNAPII in SK-N-MC cells, which in turn causes inclusion of *DHX9* exon 6A. A similar effect on *DHX9* splicing was also elicited by treatment with the chemotherapeutic drug etoposide, indicating a more general mechanism of regulation in response to DNA damage. Our data identify a new NMD-linked splicing event in *DHX9* with impact on EWS-FLI1 oncogenic activity and ES cell viability.

## INTRODUCTION

Ewing Sarcomas (ES) are aggressive tumors of bone and soft tissues. They are caused by different chromosomal translocations that yield in-frame fusion proteins comprising the amino terminus of the EWS protein fused to the carboxyl terminus of various ETS transcription factors [1]. These chimeric proteins activate a specific oncogenic program to direct neoplastic transformation [2, 3]. Among them, EWS-FLI1, generated by chromosomal translocation between chromosome 22 and 11 [3], is the most common product, representing a landmark of ES.

Both EWS and EWS-FLI1 modulate gene expression [4, 5]. Besides transcription, recent evidence unveiled a role for both proteins in the modulation of alternative splicing (AS) of target genes [6, 9]. EWS modulates AS of genes involved in the DNA damage response, including key regulators of genotoxic stress like *CHEK2,* A*BL1* and *MDM2* [8, 9]. Accordingly, EWS deficiency enhances sensitivity to ionizing radiation (IR) [10] and UV light irradiation [8]. Furthermore, two high-throughput screens identified the gene encoding EWS (*EWSR1*) as required for resistance to ionizing radiation (IR) [11] and camptothecin [12]. Importantly, in ES cells the intact *EWSR1* gene is present only on one allele, while the other allele is affected by the translocation. Thus, *EWSR1* haploinsufficiency might contribute, at least in part, to ES cells sensitivity to genotoxic stress.

DNA damage triggers the activation of signaling cascades that profoundly influence chromatin structure, thus modulating gene expression. Genotoxic stress imposed by irradiation or chemotherapeutic agents modulates AS events [7, 13], in part through reduced transcription elongation rates as a consequence of RNA Polymerase II (RNAPII) phosphorylation [14]. In this regard, mounting evidence points to aberrant AS regulation as a key step in oncogenesis [15] and indicates that splicing regulation represents a suitable target for therapeutic intervention [16].

Despite the reported links between EWS and the DNA damage response [7, 8, 10–12], whether or not changes in gene expression in response to genotoxic stress can affect the sensitivity of ES cells to irradiation has not been extensively investigated yet. In this work we identified changes in the transcriptome that are induced by low UV light irradiation in two ES cell lines (SK-N-MC and LAP-35 cells) displaying different sensitivity to UV light treatment. Among other targets, we found that UV light irradiation induced down-regulation of *DHX9* in SK-N-MC cells, partially through the generation of a new isoform that is targeted to non-mediated decay (NMD). DHX9 enhances EWS-FLI1-mediated transcription and favours anchorage-independent growth in ES cells [17]. We found that knockdown of *DHX9* in ES cells rendered them more susceptible to UV treatment, whereas its overexpression protected ES cells from irradiation. Thus, our results strongly suggest a role for DHX9 as a transcriptional co-activator of EWS-FLI1 involved in the resistance to genotoxic stress of ES cells.

## RESULTS

### SK-N-MC and LAP-35 Ewing Sarcoma cells display different resistance to UV light irradiation

**Figure 1:**
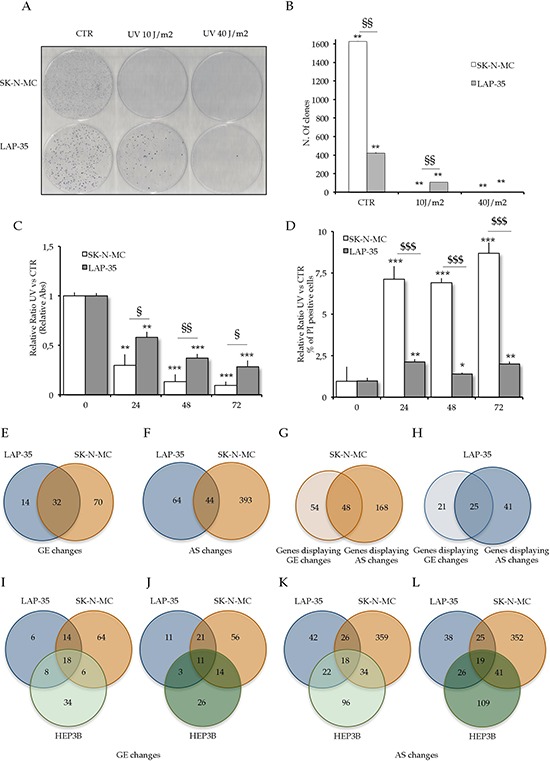
UV light irradiation triggers cytotoxic effect in Ewing Sarcoma cells **A.** Representative images of clonogenic assays of SK-N-MC and LAP-35 cells upon UV light irradiation. **B.** Histograms represent colony numbers (n = 3; mean ± s.d.) carried out on SK-N-MC (white bars) and LAP-35 cells (gray). **C.** Cell survival rates detected by MTS cell proliferation assay after 10 J/m^2^ UV light treatment in SK-N-MC (white) and LAP-35 cells (gray). **D.** Propidium Iodide (PI) viability assay; the decrease in viability was expressed as relative percentage of dead cells in treated versus control cells after 10 J/m^2^ UV light treatment in SK-N-MC (white) and LAP-35 cells (gray). In all panels statistical analysis was performed by Student's *t*-test: *p < 0.05, **p < 0.01, ***p < 0.001 for CTR vs UV treatment; $*p* < 0.05, $$*p* < 0.01, $$$*p* < 0.001 for SK-N-MC vs LAP-35 cells. **E.** Venn diagram shows the overlap of gene expression signatures at gene level induced by 10 J/m^2^ UV light irradiation in SK-N-MC and LAP-35 cells, as indicated. **F.** Venn diagram shows the overlap of gene expression signatures at AS level induced by 10 J/m^2^ UV light irradiation in SK-N-MC and LAP-35 cells, as indicated. **G.** and **H.** Venn diagrams represent the overlap of gene affected both at gene expression and AS level upon UV light irradiation in the SK-N-MC (G) and in the LAP-35 (H) cells. **I.** to **L.** Venn diagram shows the overlap of gene expression signatures at gene and AS levels induced by 10 J/m^2^ (I and K respectively) and 40 J/m^2^ (J and L respectively) UV light irradiation in ES cells (SK-N-MC and LAP-35) and HEP3B cells, as indicated.

To ascertain the efficacy of UV irradiation in suppressing the growth of ES cells, we used two ES cell lines characterized by similar chromosomal translocation [t(11;22)(q24;q12)] generating the oncogenic fusion protein EWS/FLI-1 type 1 and 2 (Figure S1A). LAP-35 [18] and SK-N-MC [19] cells were exposed to either 10 or 40 J/m^2^ UV light and clonogenic survival assays were performed by monitoring colony formation 12 days after irradiation. In the absence of irradiation, SK-N-MC cells formed 3- to 4-fold more clones than LAP-35 cells (Figure 1A, 1B), although SK-N-MC colonies displayed smaller size. When cells were exposed to 10 J/m^2^ UV light irradiation, SK-N-MC cells formed only few clones, while LAP-35 cells were still able to proliferate, albeit displaying a 8-fold reduction in clone formation with respect to untreated cells (Figure 1A, 1B). Upon treatment with 40 J/m^2^, survival of both cell lines was dramatically compromised (Figure 1A, 1B).

To corroborate the results of the colony formation assay, we performed cell proliferation assays at different time points after UV light treatment with 10 J/m^2^. SK-N-MC cell proliferation was strongly reduced by UV treatment, while the effect on LAP-35 was milder (Figure 1C). Moreover, propidium iodide (PI) staining confirmed that viability was affected by UV light irradiation more dramatically in SK-N-MC cells than in LAP-35 cells (Figure 1D).

These results indicate that SK-N-MC cells are more sensitive than LAP-35 to UV light irradiation.

### Gene expression changes induced by UV light irradiation in ES cells

To investigate whether different changes in gene expression (GE) could account for the different sensitivity of ES cells to UV light, we performed high-throughput analyses. RNA obtained from three biological replicas of SK-N-MC and LAP-35 cells treated with low doses of UV light (10 J/m^2^) was hybridized to splicing sensitive microarrays featuring 1804 AS events in 482 genes encoding proteins with functions related to RNA processing and cancer [8, 14, 20]. Hybridization analysis revealed that 102 genes in SK-N-MC cells and 46 genes in LAP-35 cells change their expression levels upon UV light treatment (fold change > |1,3|; *p* value < 0,01; Figure 1E, Supplementary Table S1). Of these, 41 in SK-N-MC and 14 genes in LAP-35 cells displayed a fold change > |2| (Figure S1B). Most genes were downregulated after treatment, with a more pronounced effect in SK-N-MC than in LAP-35 cells and with 32 genes common in the two cell lines (Figure 1E, S1C). Nevertheless, 4 genes in SK-N-MC cells (*CD82, TRIB3, YWHAH, AFF2*) and 3 genes in LAP-35 cells (*CDKN1A, FOS, GADD45A)* were upregulated (Figure S1D).

Array analysis indicated that UV irradiation exerted a more pronounced effect on AS than transcription. 437 AS events in 216 genes were affected in SK-N-MC cells, and 108 AS events in 66 genes were affected in LAP-35 cells considering fold change >|1,4|, *z* score >3; *p* value <0,01 (Figure 1F; Supplementary Table S1), or 211 in SK-N-MC and 37 in LAP-35 cells considering fold change >|2| (Figure S1F). UV light irradiation impacted all types of AS events present in the array (Figure S1E). Among these, 44 events in 41 genes were conserved in the two cell lines, indicating a certain degree of overlap in the splicing response of ES cells to UV irradiation. The proportion of genes that display regulation at both AS and expression levels is substantially higher in LAP-35 (37.9%) than in the SK-N-MC cells (22.2%) (Figure 1G, 1H).

RT-quantitative PCR (RT-qPCR), using oligonucleotides covering both exons and exon-exon junctions, of all 8 randomly selected AS changes (in the *C1QBP, SIP1, U2AF35, TNFRSF10B*, *AURKB, BAT1, CCNA2* and *CROP* genes) identified by microarrays were validated (Figure S2A–S2H), thus confirming the fidelity of the microarray analysis. In order to evaluate the specificity of UV light response in different cancer cell lines, we performed microarray analysis of HEP3B (hepatocellular carcinoma) cells irradiated with 10J/m^2^ UV light (Figure 1I, 1K) and also compared our datasets with a previous dataset obtained from treatment of HEP3B with 40J/m^2^ UV light (14; Figure 1J, 1L). As expected, a more consistent overlap at GE level was observed between cells treated with the same UV intensity (Figure 1I and 1J). Interestingly, 18 genes affected at the expression level were conserved in the three cell lines (Figure 1I), and 10 of them were also affected upon 40 J/m^2^ irradiation (not shown). Furthermore, we identified 18 AS events affected by UV light irradiation independently from the cellular context (Figure 1K), 11 of them were also conserved upon 40J/m^2^ irradiation (not shown). Notably, SK-N-MC cells are more responsive to UV light irradiation than other cell lines, at both GE level (102 genes affected versus 46 in LAP-35 and 66 in HEP3B) and AS level (437 AS events modulated versus 108 in LAP-35 and 170 in HEP3B; Figure 1I–1L).

### *DHX9* is selectively regulated by UV light irradiation in SK-N-MC cells

**Figure 2:**
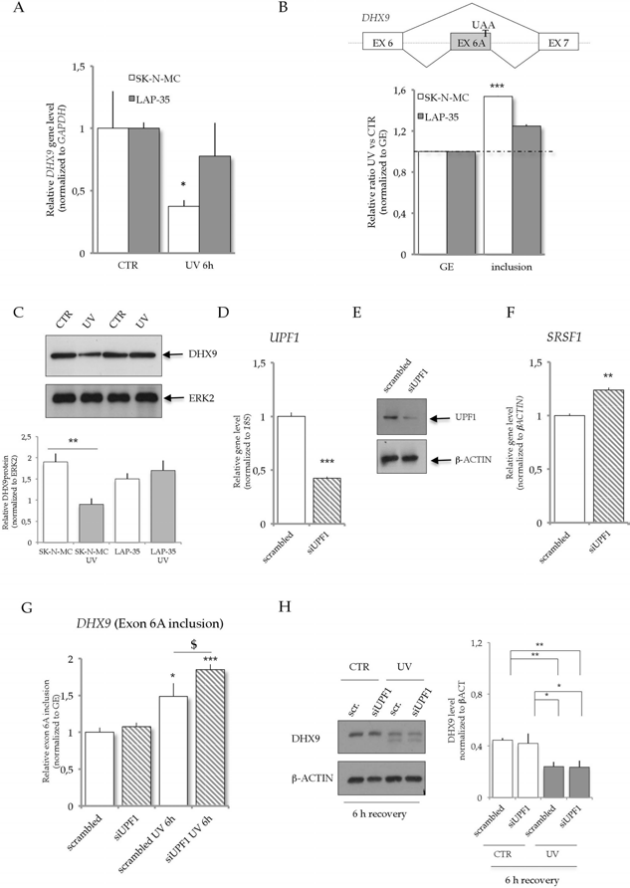
UV light irradiation affects *DHX9* mRNA expression and alternative splicing in SK-N-MC cells, but not in LAP-35 **A.** RT-qPCR validation of microarray-predicted GE changes in *DHX9* mRNA (exon 4), normalized to *GAPDH*. **B.** Scheme of *DHX9* AS event (top panel). The alternative exon 6A is upregulated in SK-N-MC cells upon UV light treatment. UAA indicates the stop codon within exon 6A. Histograms represent levels of expression of exon 6A as relative ratio of cells treated with 10 J/m^2^ UV light versus untreated normalized to a constitutive exon (GE). White bars indicate SK-N-MC cells while gray bars indicate LAP-35 cells. (*n* = 3; mean ± s.d.). **C.** Western blot analysis of DHX9 and ERK2 expression in LAP-35 and SK-N-MC cells upon UV light irradiation. 10 μg of proteins from SK-N-MC and LAP-35 cell extracts after UV light (10 J/m^2^) treatment were loaded. Histograms represent the quantification of DHX9 protein normalized to ERK2. **D.** RT-qPCR analysis of *UPF1* expression in SK-N-MC cells transfected with either scrambled (white) or *siUPF1* (filled) oligonucleotides. Histograms represent *UPF1* mRNA levels normalized to *18S* expression (*n* = 3; mean ± s.d). **E.** Western blot of UPF1 and β-actin expression in SK-N-MC cells transfected with either scrambled (white) or *siUPF1* (filled) oligonucleotides. 10 μg of proteins were loaded. **F.** RT-qPCR analysis to detect the expression of *SRSF1*, a known NMD target [23], in SK-N-MC cells transfected with either scrambled (white) or *siUPF1* (filled) oligonucleotides. Histograms represent *SRSF1* mRNA levels normalized to *β-ACTIN* expression (*n* = 3; mean ± s.d). **G.** Relative *DHX9* exon 6A inclusion (normalized to GE) in SK-N-MC cells, transfected either with scrambled or *siUPF1* oligonucleotides, with or without UV treatment. **H.** Western blot analysis of DHX9 and β-ACTIN expression in SK-N-MC cells at 6 hours of recovery after UV light irradiation. 10 μg of proteins from SK-N-MC cell extracts were loaded transfected either with scrambled or *siUPF1* oligonucleotides. Upon UV light treatment a slower band of DHX9 protein is also induced due to caspase cleavage of the first 95 aminoacids of the protein and correlating with early stage of apoptosis, cell apoptosis as previously described [24]. On the right, histograms represent the quantification of DHX9 protein normalized to β-ACTIN from three independent experiments (*n* = 3; mean ± s.d.). In all panels statistical analysis was performed by Student's *t*-test: *p < 0.05, **p < 0.01, ***p < 0.001; $*p* < 0.05, $$*p* < 0.01, $$$*p* < 0.001.

Among the genes that were differentially regulated in the two ES cell lines, we focused on *DHX9* because of its reported functional interaction with EWS-FLI1 [17]. DHX9, also known as RNA helicase A (RHA) or nuclear DNA helicase II (NDHII), displays helicase activity and modulates transcription through its interaction with RNAPII [21]. RT-qPCR analysis confirmed that UV light irradiation significantly repressed *DHX9* mRNA expression in SK-N-MC, but not in LAP-35 cells (Figure 2A). Notably, the microarray analysis indicated that UV light treatment also affected AS of the *DHX9* gene in SK-N-MC cells, leading to inclusion of exon 6A and resulting in expression of a previously uncharacterized *DHX9* isoform (Figure 2B). This predicted AS event was validated by RT-qPCR using different sets of primers in SK-N-MC cells, whereas it was not significantly modulated in LAP-35 cells (Figure 2B, S3A–S3E). Moreover, western blot analysis confirmed that UV treatment caused down-regulation of the DHX9 protein in SK-N-MC but not in LAP-35 cells (Figure 2C). *DHX9* expression levels and exon 6A inclusion were not affected in HEP3B cell upon UV light irradiation (Figure S3G, Supplementary Table S1), suggesting that regulation of *DHX9* pre-mRNA processing represents a specific response of SK-N-MC ES cells to low doses of UV light.

Exon 6A contains a premature stop codon (PTC), which should lead to NMD of this new transcript (NR_033302), thus possibly contributing to the decrease in the pool of *DHX9* mRNA upon UV light treatment. Indeed, UV light-induced inclusion of exon 6A in SK-N-MC cells was significantly stabilized after knockdown of *UPF1,* (Figure 2D–2G), an essential component of the NMD pathway [22], similarly to the effect on *SRSF1* (Figure 2F), a known target of NMD [23]. These results suggest that this new *DHX9* transcript is targeted by NMD.

Moreover, actinomycin D treatment at 5 hours after UV light irradiation completely abolished the accumulation of the exon 6A-containing *DHX9* transcript, which returned to basal levels within 30 minutes from the block of transcription (Figure S4A). In the absence of actinomycin D, this variant accumulates up to 10 hours after UV irradiation, and returned to basal levels by 24 hours (Figure S4A). This result indicates that expression of the NMD-targeted *DHX9* variant is a transient response to UV irradiation. As expected, western blot analysis indicated that knockdown of *UPF1* had no effect on the UV light-induced reduction of full length DHX9 protein in SK-N-MC cells 6 hours after UV light irradiation (Figure 2H).

Collectively these results indicate that UV light irradiation causes down-regulation of *DHX9* mRNA in SK-N-MC by changes in gene expression and AS-mediated NMD.

To investigate the relative stability of DHX9 protein in normal conditions and upon UV light irradiation, we treated SK-N-MC cells with cycloheximide and harvested them at different time points (2, 6 and 24 hours) after treatment. Western blot analyses revealed that full length DHX9 protein is stable in the first 6 hours of cycloheximide treatment, whereas its expression levels are halved by 24 hours of treatment (Figure S4B and S4C). However, UV light irradiation dramatically reduced the half-life of DHX9 protein, causing a 30% reduction after 6 hours and about 80% reduction after 24 hours (Figure S4B and S4C). Notably, treatment with CHX did not further decrease the levels of DHX9, suggesting that de novo protein synthesis does not compensate the effect of UV irradiation on DHX9 protein levels (Figure S4B and S4C). Moreover, inhibition of the proteasome by treatment with MG132 prevented UV-induced DHX9 degradation and it also prevented the degradation of the caspase 3-cleaved DHX9 [24], which is induced upon UV light treatment and rapidly degraded in the absence of MG132 (Figure S4D and S4E). Collectively these experiments show that UV light irradiation induces DHX9 downregulation partly through its degradation by the proteasome and partly through induction of a new alternative isoform that is targeted by NMD, thus limiting de novo DHX9 protein synthesis.

### Reduced RNAPII processivity enhances *DHX9* exon 6A inclusion in SK-N-MC cells exposed to UV light irradiation

**Figure 3:**
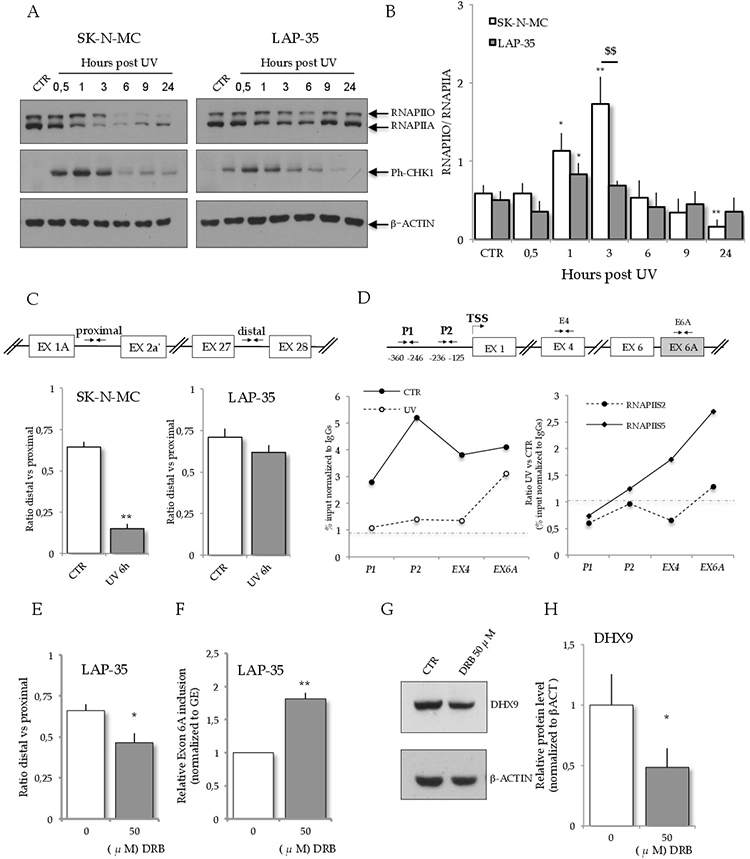
RNAPII dynamics in ES cells upon UV light treatment **A.** Western blot analysis of RNAPII, Phospho-CHK1 (in order to control the activation of a protein kinase-signaling cascade initiated by ATM and ATR protein kinases upon DNA damage) and β-ACTIN expression in SK-N-MC and LAP-35 cells after UV light (10 J/m^2^) treatment. **B.** Histograms represent the ratio between the hyper-phosphorylated (RNAPIIO) and the hypo-phosphorylated (RNAPIIA) RNAPII from three independent experiments in SK-N-MC (white bars) and LAP-35 (grey bars) cells (mean ± s.d.). Statistical analysis was performed by Student's *t*-test: *p < 0.05, **p < 0.01, for treated vs untreated; $$*p* < 0.01 for SK-N-MC vs LAP-35 cells. **C.** In the upper part, scheme of *DHX9* transcription unit showing the primers (arrows) designed to amplify proximal and distal amplicons in RT-qPCR analysis. Histograms represent RNAPII processivity, determined as a ratio between the distal and proximal amplicons in *DHX9* pre-mRNA in control (white) and UV (10 J/m^2^) treated (grey) SK-N-MC (on the left) and LAP-35 cells (on the right). **D.** ChIP analysis of RNAPII occupancy on *DHX9* transcription unit in normal condition and upon UV light treatment. In the upper part, scheme of *DHX9* transcription unit showing the primers (arrows) designed to amplify the promoter and amplicons in the constitutive exon 4 and in the alternative exon 6A. On the left, chart represents total RNAPII binding as input percentage normalized to IgGs in normal conditions (continuous line) and at 6 hours upon UV light treatment (dashed line). On the right, chart represents PhosphoSer2-RNAPII (continuous line) and PhosphoSer5-RNAPII (dashed line) binding to DHX9 transcription unit. Binding is expressed as input percentage of UV versus CTR. **E–F.** RNAPII processivity and relative exon 6A inclusion after 12-hours 50 μM-DRB treatment in LAP-35 cells. Exon 6A inclusion rises and RNAPII processivity decreases in DRB-treated LAP-35. Histograms represent mean ± s.d. from three independent biological replicas (treated vs untreated: *p < 0.05, **p < 0.01). **G.** Western blot analysis of DHX9 and β-ACTIN expression in extracts (10 μg) from LAP-35 treated with 50 μM DRB (12 hours). **H.** Histograms represent quantification of DHX9 protein normalized to β-ACTIN (*n* = 3; mean ± s.d.; **p < 0.01).

UV light irradiation is known to widely affect gene expression, including regulation of genes involved in the DNA damage response [25]. UV irradiation induces hyper-phosphorylation of the carboxy terminal domain (CTD) of RNAPII, followed by its ubiquitylation and degradation [14, 26]. Importantly, the phosphorylation status of the RNAPII CTD correlates with distinct functions of the polymerase, as the hypo-phosphorylated form (RNAPIIA) is associated with transcriptional initiation whereas the hyper-phosphorylated form (RNAPIIO) correlates with transcriptional elongation [27]. UV light irradiation can also influence splicing decisions through modulation of the RNAPII elongation rate [14, 28–29], representing an example of kinetic coupling between transcription and splicing. Upon genotoxic stress, phosphorylation of RNAPII CTD slows down the polymerase, offering a time window opportunity for weak exons to be efficiently recognized by the splicing machinery [14]. *DHX9* exon 6A displays a low score for the 5′splice site (5.9; AAG | GTCAGT) if compared with the constitutive downstream exon (12.4; CAG | GTAAGT; see Material and Methods for calculation details), rendering it relatively weak. Thus, we asked whether the differential regulation of *DHX9* AS and expression by UV light in ES cells correlated with differential effects on RNAPII phosphorylation. Western blot analysis using an antibody against total RNAPII, which recognizes both RNAPIIO and RNAPIIA, showed that RNAPII is strongly downregulated in SK-N-MC cells after 3–6 hours from UV light treatment, whereas this effect was weaker and more transient in LAP-35 cells (Figure 3A; Figure S5A, S5B). Moreover, the phosphorylation status of the CTD (RNAPIIO) was different in the two cell lines. RNAPIIO peaked at 1–3 hours after UV light treatment in SK-N-MC cells, and then it was strongly reduced (Figure 3A, 3B); on the contrary, CTD phosphorylation was only slightly increased in LAP-35 cells and it was maintained almost constant in the 24 hours following the UV light treatment (Figure 3A, 3B).

The different dynamics of RNAPII phosphorylation in the two ES cell lines exposed to UV irradiation raises the possibility that *DHX9* expression and exon 6A splicing are affected by modulation of RNAPII elongation rate in SK-N-MC. To test this hypothesis, we first analyzed the accumulation of distal and proximal *DHX9* pre-mRNA and calculated RNAPII processivity as ratio between them (Figure 3C) [30]. Remarkably, UV light irradiation significantly reduced RNAPII processivity within the *DHX9* transcription unit in SK-N-MC cells but not in LAP-35 cells (Figure 3C).

To further test our hypothesis, we analyzed RNAPII occupancy in the *DHX9* transcription unit upon UV light irradiation by chromatin immunoprecipitation experiments (ChIP) in SK-N-MC cells. Although RNAPII binding to *DHX9* gene was generally decreased by UV treatment, we observed a peak in occupancy near the alternative exon 6A after 6 hours of treatment (Figure 3D, left panel). Moreover, by using phospho-specific antibodies we found that UV treatment caused a progressive increase in the pausing (Ser5 phosphorylated) RNAPII, with maximal enrichment in the variant exon 6A (Figure 3D, right panel). By contrast, RNAPII phosphorylated in Ser2, which correlates with the elongating phase of the polymerase [31], was only marginally affected by the treatment (Figure 3D, right panel). This result indicates that UV treatment causes a local pausing of RNAPII near exon 6A.

Next, we asked whether *DHX9* exon 6A inclusion was favoured by the lower processivity of RNAPII induced by UV-light irradiation in SK-N-MC cells. To this end, we pharmacologically inhibited RNAPII elongation in LAP-35 cells by treatment with 5, 6-Dichlorobenzimidazole1-b–D-ribofuranoside (DRB) [32, 33] in the absence of genotoxic stress. RT-qPCR analysis confirmed that DRB treatment reduced RNAPII processivity in LAP-35 cells (Figure 3E). Strikingly, this was sufficient to induce exon 6A inclusion (Figure 3F) and downregulation of *DHX9* mRNA and protein in these cells (Figure 3G, 3H).

These experiments argue that modulation of RNAPII processivity upon UV light irradiation in SK-N-MC cells promotes inclusion of exon 6A in the *DHX9* gene, contributing to down-regulation of its expression.

### UV light irradiation affects EWS-FLI1 recruitment on target genes

**Figure 4:**
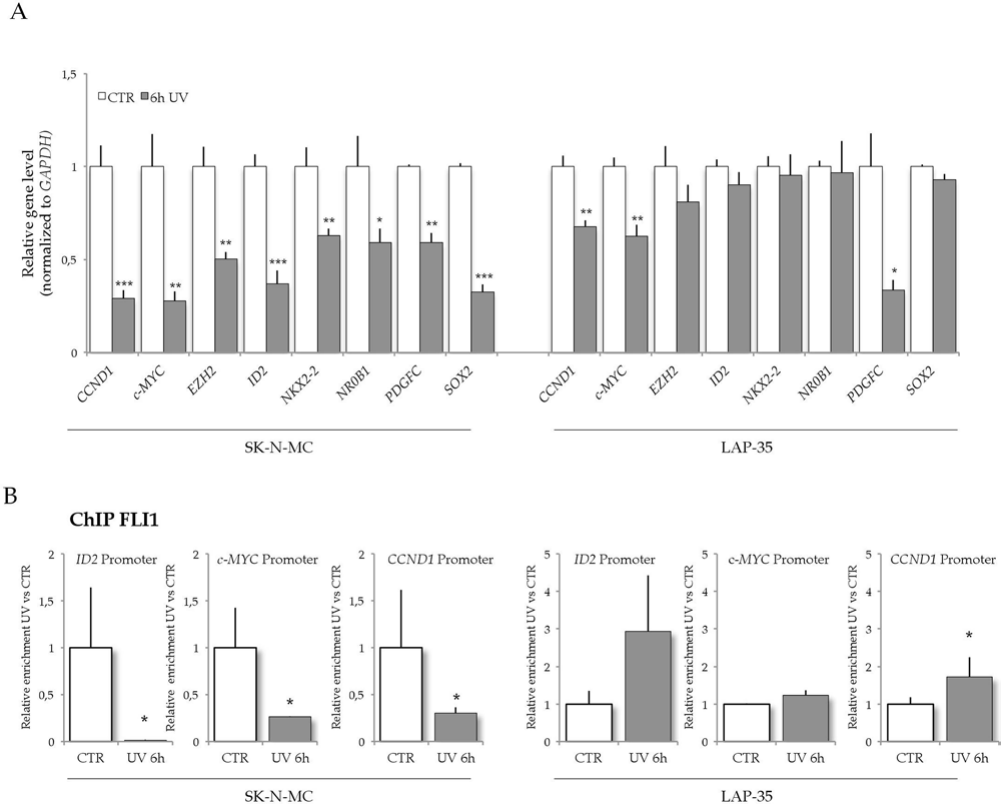
UV light treatment down-regulates EWS-FLI1 target genes in SK-N-MC cells **A.** Histograms represent RT-qPCR analysis of *ID2*, *c-MYC*, *CCND1 EZH2, NKX2–2, NR0B1, PDGFC, SOX2* expression in control (white bar) or UV-treated (gray bar) LAP-35 and SK-N-MC cells. GE values of are normalized to *GAPDH* expression (*n* = 3; mean ± s.d.; treated vs untreated *p < 0.05, **p < 0.01, ***p < 0.001). **B.** Association of EWS-FLI1 to the promoters of *ID2*, *CCND1* and *c-MYC* genes. qPCR analysis of EWS-FLI1 ChIP signals for SK-N-MC and LAP-35 cells with or without UV treatment. Histograms represent relative fold enrichment of EWS-FLI1 normalized versus the IgGs content (*n* = 3; mean ± s.d.; *p < 0.05, **p < 0.01, ***p < 0.001).

DHX9 protein directly interacts with EWS-FLI1 and enhances EWS-FLI1-mediated transcription, cooperating with EWS-FLI1-induced oncogenic transformation [17]. Thus, we asked if UV light irradiation affected transcription of EWS-FLI1 target genes through *DHX9* downregulation. *Cyclin D1* (*CCND1)*, *c-MYC*, and DNA-binding protein inhibitor *ID-2* (*ID2*) genes encode proteins strictly involved in cell proliferation and cancer development [34]. In particular, *ID2* expression is upregulated in ES cells as a result of both direct EWS-FLI1 binding to its promoter and increased *c-MYC* expression [34]. RT-qPCR analysis revealed a strong downregulation of *ID2* expression (60% relative to untreated) upon UV irradiation in SK-N-MC cells, whereas the gene was unaffected in LAP-35 cells. Furthermore, *c-MYC* and *CCND1* genes were downregulated at higher extent in SK-N-MC (72% and 71%, respectively) than in LAP-35 cells (38% and 32%, respectively; Figure 4A). To corroborate our results we analyzed five more genes known to be direct target of EWS-FLI1. Notably, the expression of *EZH2, NKX2–2, NR0B1,* and *SOX2* was specifically decreased in SK-N-MC cells by UV light treatment, whereas *PDGFC* expression was downregulated in both cell lines (Figure 4A). These results show that UV light irradiation induces widespread down-regulation of EWS-FLI1 target genes in SK-N-MC cells.

Since DHX9 enhances EWS-FLI1 transcriptional activity, its downregulation upon UV light irradiation may directly affect EWS-FLI1 function. To test if UV light irradiation affected the recruitment of EWS-FLI1 to target genes, we performed chromatin immunoprecipitation (ChIP) experiments in both SK-N-MC and LAP-35 cells. Recruitment of EWS-FLI1 to *ID2*, *CCND1*, and *c-MYC* promoters was strongly impaired by UV light treatment in SK-N-MC, but not in LAP-35 cells where we actually observed the opposite trend (Figure 4B), even though this enrichment did not result in increased expression of the target genes (Figure 4A). Accordingly, the interaction between DHX9 and EWS-FLI1 was not affected by UV light treatment in LAP-35 cells (Figure S5C, S5D).

These results suggest that downregulation of DHX9 expression induced by UV light irradiation in SK-N-MC cells impairs recruitment of EWS-FLI1 on the promoters of its target genes.

### DHX9 is involved in modulation of EWS-FLI1 target genes

**Figure 5:**
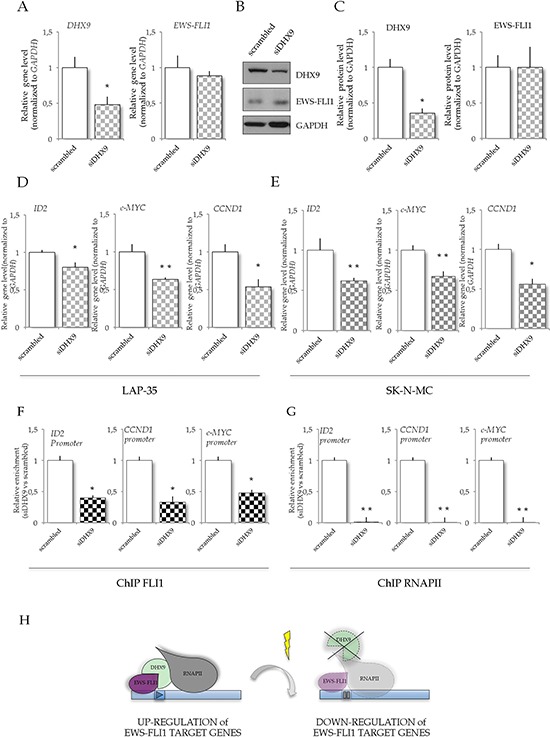
DHX9 knockdown in LAP-35 cells affects EWS-FLI1 target genes **A.** RT-qPCR analysis to monitor *DHX9* and *EWS-FLI1* mRNA expression in LAP-35 cells knockdown for *DHX9*. **B.** Western blot analysis of DHX9, FLI1 and GAPDH expression in extracts (10 μg) from cells transfected with either scrambled or *siDHX9* oligonucleotides, prepared 72 hr after transfection. **C.** Histograms represent quantification of western blot signals shown in (B) from three independent experiments (mean ± s.d.). **D.** RT-qPCR analysis of EWS-FLI1 target genes upon *DHX9* knockdown in LAP-35 cells. Histograms represent expression (GE) of *ID2*, *c-MYC*, and *CCND1* normalized to *GAPDH* expression in cells transfected with either scrambled (white bars) or *siDHX9* oligonucleotides (checked filled bars) (*n* = 3; mean ± s.d.; treated vs untreated: *p < 0.05). **E.** RT-qPCR analysis of EWS-FLI1 target genes upon *DHX9* knockdown in SK-N-MC cells. Histograms represent expression (GE) of *ID2*, *c-MYC*, *CCND1* normalized to *GAPDH* expression in cells transfected with either scrambled (white bars) or *siDHX9* oligonucleotides (checked filled bars) (*n* = 3; mean ± s.d.). In all panels statistical analysis was performed by Student's *t*-test: *p < 0.05, **p < 0.01, ***p < 0.001). **F.** and **G.** Association of EWS-FLI1 (F) and RNAPII (G) to the promoters of *ID2*, *CCND1* and *c-MYC* genes. qPCR analysis of EWS-FLI1 and RNAPII ChIP signals for LAP-35 knocked down for *DHX9*. Histograms represent relative fold enrichment of EWS-FLI1 and RNAPII binding normalized versus the IgGs content (*n* = 3; mean ± s.d.; *p < 0.05, **p < 0.01, ***p < 0.001). **H.** Hypothetical model of DHX9 regulation of gene expression upon UV light treatment. In ES cells DHX9, acting as hinge between EWS-FLI1 and RNAPII, is involved in EWS-FLI1 target gene expression; upon UV irradiation, the *DHX9* expression is down-regulated in SK-N-MC cells, thus decreasing DHX9 availability and, in turn, interfering with EWS-FLI1 target gene expression. In LAP-35 cells, UV light treatment does not affect *DHX9* expression and does not impair the recruitment of RNAPII on the promoters of EWS-FLI1 target genes.

To endorse the hypothesis that expression of EWS-FLI1 target genes in response to UV irradiation was linked to *DHX9* expression in ES cells, we knocked down *DHX*9 in LAP-35 cells. Reduction of *DHX9* expression was confirmed both at the RNA (52% reduction versus scrambled; Figure 5A) and protein level (64% reduction versus scrambled; Figure 5B, 5C). RT-qPCR analysis revealed that *DHX9* knockdown affected expression of EWS-FLI1 target genes in LAP-35 cells, leading to down-regulation of *ID2* (20%), *CCND1* (46%) and *c-MYC* (40%) mRNA levels (Figure 5D). Importantly, RT-qPCR and western blot analysis revealed that knockdown of *DHX9* expression did not affect EWS-FLI1 expression in LAP-35 cells (Figure 5B, 5C), ruling out that the effect on EWS-FLI1 target genes was indirectly due to modulation of its expression. Furthermore, knockdown of *DHX9* in SK-N-MC cells recapitulated the effect of UV treatment on the expression of EWS-FLI1 target genes (Figure 5E). No major changes in RNAPII phosphorylation were detected upon DHX9 knockdown in SK-N-MC cells (Figure S4E, S4F), suggesting that DHX9 acts directly on EWS-FLI1.

To further corroborate our hypothesis, we analyzed the recruitment of EWS-FLI1 and RNAPII on EWS-FLI1 target genes by ChIP analysis in LAP-35 cells. *DHX9* knockdown strongly impaired recruitment of both EWS-FLI1 and RNAPII on the promoters of EWS-FLI1 target genes (Figure 5F, 5G).

These data suggest that reduced expression of DHX9 induced by low doses of UV light irradiation contributes to downregulation of EWS-FLI1 target genes in ES cells (Figure 5H).

### DHX9 confers UV light resistance to ES cells

**Figure 6:**
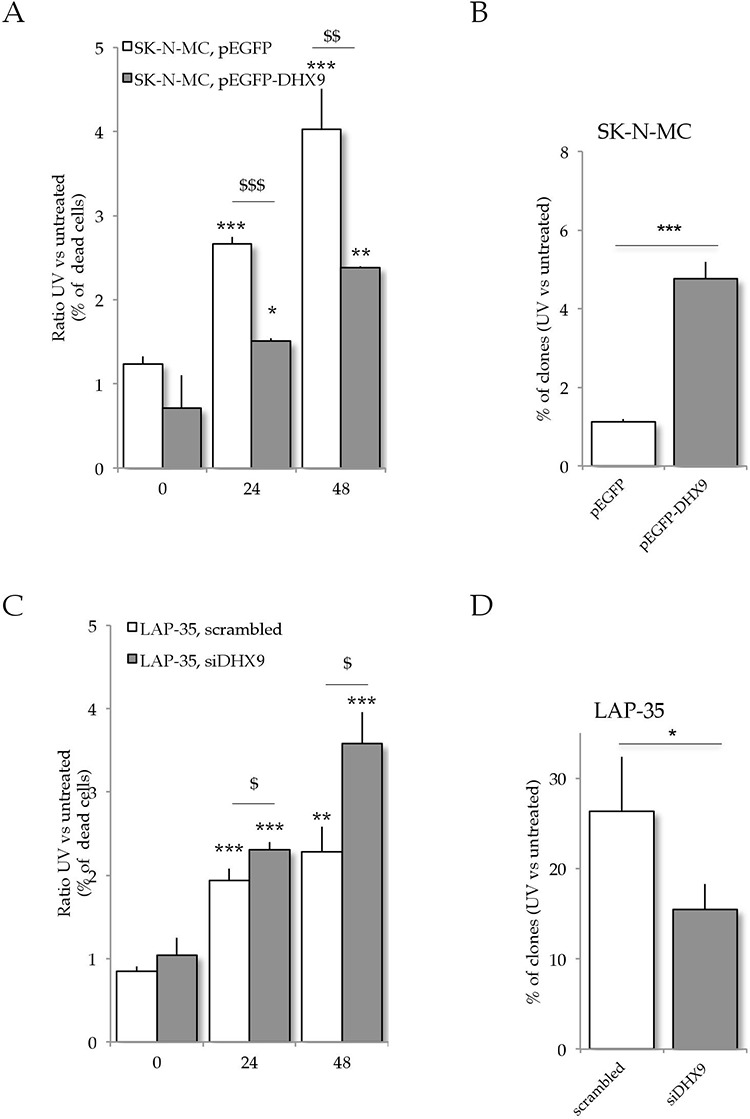
DHX9 expression affects ES resistance to UV light irradiation **A.** SK-N-MC cells were transfected with either pEGFP or pEGFP-DHX9 constructs and exposed to 10 J/m^2^ UV light 48 hours after transfection. Cell death was detected at 0, 24 and 48 hours after the UV treatment by PI staining and flow cytometry analysis; histograms represent the ratio of UV treated vs untreated PI positive cells in the GFP population (*n* = 3; mean ± s.d.; *p < 0.05, **p < 0.01, ***p < 0.001 for treated vs untreated; and: $*p* < 0.05, $$*p* < 0.01, $$$*p* < 0.001 for pEGFP vs pEGFP-DHX9). **B.** GFP-positive SK-N-MC cells transfected as in (A) were sorted and plated (2000 cells per 10-mm plate) in IMDM complete medium. 12 days after 10 J/m^2^ UV-light treatment cells were scored for clonogenic activity. Histograms represent the percentage of colonies formed after UV treatment versus untreated carried out on pEGFP (white) or pEGFP-DHX9 (gray) (*n* = 3; mean ± s.d.; pEGFP vs pEGFP-DHX9: ***p < 0.001). **C.** LAP-35 cells were transfected with either scrambled or *siDHX9* oligonucleotides and exposed to 10 J/m^2^ UV light 48 hours after transfection. Cell death was detected at 0, 24 and 48 hours after treatment by PI staining and flow cytometry analysis; histograms represent the ratio of UV treated vs untreated PI positive cells (*n* = 3, mean ± s.d.; treated vs untreated: *p < 0.05, **p < 0.01, ***p < 0.001, for treated vs untreated; and: $*p* < 0.05, $$*p* < 0.01, $$$*p* < 0.001 for scrambled vs *siDHX9*. **D.** LAP-35 cells were transfected with either scrambled or *siDHX9* oligonucleotides together with pEGFP plasmid; GFP positive cells were sorted and plated as above. 12 days after 10 J/m^2^ UV light treatment, cells were scored for clonogenic activity. Histograms represent the percentage of colonies after UV treatment versus untreated cells carried out on scrambled (white bars) or *siDHX9* (gray) LAP-35 cells (*n* = 3, mean ± s.d.; scrambled vs siDHX9: *p < 0.05).

DHX9 expression is necessary for ES cells to proliferate [17, 35]. To test whether its expression is also relevant for ES cell survival in response to UV light irradiation, we overexpressed recombinant GFP or GFP-DHX9 in SK-N-MC cells. PI staining at 0, 24 and 48 hours after UV light treatment revealed that DHX9-overexpressing SK-N-MC cells are significantly more resistant to UV light treatment (Figure 6A). This result was also supported by clonogenic assays. SK-N-MC cells were transfected with either GFP or GFP-DHX9 and GFP positive cells were isolated by cell sorting before the treatment with low doses UV light. Up-regulation of DHX9 in SK-N-MC cells significantly enhanced clonogenic activity in response to UV light treatment (Figure 6B).

To further prove the involvement of DHX9 in the resistance of ES cells to UV irradiation, we knocked down its expression in LAP-35 cells. In line with our hypothesis, knockdown of *DHX9* in LAP-35 cells reduced resistance to UV light treatment, as indicated by the higher number of PI-positive cells at 24–48 hours after irradiation (Figure 6C) and the reduced number of colonies observed in clonogenic assays (Figure 6D). Interestingly, transfection of recombinant GFP-DHX9 in HeLa cells, which do not express the EWS-FLI1 oncogene, did not improve resistance to UV light irradiation (Figure S6A, S6B), indicating that DHX9 function is mediated by an ETS transcription factor (i.e. EWS-FLI1).

These results strongly indicate that DHX9 expression confers resistance to UV light irradiation to ES cells and suggest that downregulation of DHX9 expression or activity could be instrumental to sensitize ES cells to genotoxic stress.

### Genotoxic stress affects *DHX9* alternative splicing

**Figure 7:**
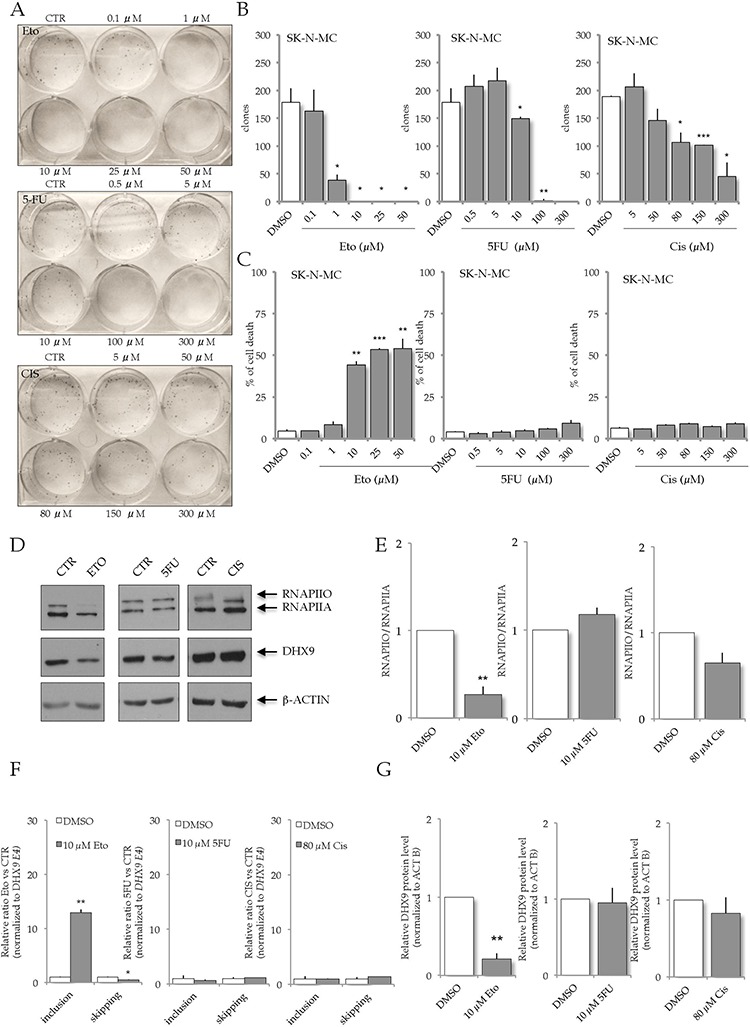
Etoposide treatment affects DHX9 expression and ES sensitivity **A.** Representative images of clonogenic assays of SK-N-MC cells upon treatment with different concentration of etoposide (Eto; from 0,1 to 50 μM), 5-fluorouracile (5-FU; from 0,5 to 300 μM), and cisplatin (CIS; from 5 to 300 μM). **B.** Histograms represent colony numbers (*n* = 3; mean ± s.d.) carried out on SK-N-MC treated with Eto, 5-FU and CIS (grey bars) versus DMSO treatment (white bars). **C.** Propidium Iodide (PI) viability assay; the decrease in viability was expressed as relative percentage of dead cells in treated (grey bars) versus control (white bars, DMSO) cells after 16 hours of Eto, 5FU and CIS treatment. In all panels statistical analysis was performed by Student's *t*-test: *p < 0.05, **p < 0.01, ***p < 0.001. **D.** Western blot analysis of RNAPII, DHX9 and β-ACTIN expression in SK-N-MC after 16 hours treatment with DMSO, Eto (10 μM), 5-FU (10 μM), and CIS (80 μM). 10 μg of extracts were loaded in each lane of a 6% SDS PAGE. **E.** Histograms represent the ratio between the hyper-phosphorylated (RNAPIIO) and the hypo-phosphorylated (RNAPIIA) RNAPII from three independent experiments in SK-N-MC after 16 hours treatment with DMSO (white bars) or Eto (10 μM), 5FU (10 μM), and CIS (80 μM) (grey bars), as in (D) (mean ± s.d.). Statistical analysis was performed by Student's *t*-test: *p < 0.05, **p < 0.01, ***p < 0.001 **F.** RT-qPCR analysis to monitor *DHX9* AS upon Eto (10 μM), 5-FU (10 μM), and CIS (80 μM) treatment. Histograms represent levels of expression of *DHX9* exon 6A normalized to a constitutive exon (E4) in SK-N-MC cells. **G.** Histograms represent DHX9 expression from three independent experiments in SK-N-MC treated for 16 hours with DMSO (white bars) or Eto (10 μM), 5-FU (10 μM), and CIS (80 μM) (grey bars), as in (D) Statistical analysis was performed by Student's *t*-test: *p < 0.05, **p < 0.01, ***p < 0.001.

In order to test whether other genotoxic agents affected SK-N-MC cells viability, we performed clonogenic and cell viability assays using increasing concentrations of etoposide (Eto), 5-fluorouracile (5FU) and cisplatin (CIS). These drugs are widely used in chemotherapy for their antineoplastic properties [36]. Etoposide is a topoisomerase II inhibitor, 5-fluorouracile is a pyrimidine analog, and cisplatin is an alkylating agent; all of them induce DNA damage and cell death [36]. We found that etoposide treatment specifically impaired SK-N-MC cells proliferation and clonogenicity, while 5-fluorouracile and cisplatin had milder effect on cell viability (Figure 7A–7C). Importantly, western blot analysis documented that etoposide was also the only chemotherapeutic drug affecting RNAPII phosphorylation (Figure 7D, 7E), in a manner similar to what observed at later time points after UV irradiation (Figure 3A). Furthermore, etoposide treatment also enhanced *DHX9* exon 6A inclusion (Figure 7F), thus decreasing DHX9 protein expression (Figure 7D–7G) similarly to UV light irradiation.

These results document that etoposide treatment parallels the effects of UV light irradiation on *DHX9* alternative splicing, RNAPII phosphorylation and SK-N-MC viability. Accordingly, etoposide treatment displayed a milder effect on LAP35 cells (Figure S6C). On the other hand, treatment of both LAP-35 and SK-N-MC cells with the YK-4–279 inhibitor, which inhibits DHX9-EWS-FLI1 interaction, affects viability and proliferation of both ES cell lines (Figure S6D, S6E).

## DISCUSSION

The study presented here identifies DHX9 as a novel regulator of ES cells sensitivity to UV light irradiation. We provide evidence that DNA damage regulates *DHX9* expression by AS through the inclusion of a new PTC-containing exon and consequent targeting to NMD. UV light regulates this event in a CTD-dependent manner by slowing down the elongation rate of RNAPII and promoting the inclusion of the alternative exon 6A in *DHX9* pre-mRNA. Furthermore, we show that changes in expression of *DHX9* modify the sensitivity to UV irradiation and that DHX9 is a novel pro-survival factor in ES cells. Notably, among other chemotherapeutic agents tested, etoposide, the only one that efficiently suppressed ES cell growth, was also the only agent affecting RNAPII phosphorylation and exon 6A splicing. These results identify modulation of *DHX9* splicing as a new potential tool to enhance ES cell sensitivity to genotoxic stresses.

Cancer cells are characterized by alteration of gene expression both at the level of transcription and mRNA processing, giving rise to many splice variants specifically associated with cancer [15]. In particular, AS plays a key role in the cellular response to stress and DNA damage, influencing the response to chemotherapeutic agents [7, 8]. Notably, splicing factors and components of the transcription machinery, including RNAPII itself, can be targets of intracellular cascades that link changes in splicing patterns to DNA damage signals [14]. For instance, UV light treatment triggers the inhibition of transcription initiation by RNAPII [23]. Furthermore, the BRCA-1/BARD-1 complex ubiquitinates the phosphorylated form of RNAPII, inducing its degradation by the proteasome [37]. Thus, the timing and dynamics of RNAPII phosphorylation and degradation take part to the fine-tuned regulation of AS upon UV light irradiation ([14]; present study). In this regard, we found that regulation of RNAPII correlates with the sensitivity of ES cells to UV irradiation. RNAPII is strongly downregulated in SK-N-MC cells 3–6 hours after UV light treatment, whereas it was only slightly reduced in LAP-35 cells (Figure 3A, 3B). Moreover, the phosphorylation status of the CTD (RNAPIIO) was regulated differently in the two cell lines; RNAPIIO peaked at three hours upon UV light treatment in SK-N-MC cells, and then it was strongly reduced. On the contrary, the phosphorylation status of the CTD was only slightly affected in LAP-35 cells under these conditions (Figure 3A, 3B). In line with the effects on RNAPII dynamics, larger changes in gene expression and AS were observed in SK-N-MC than in LAP-35 cells, suggesting that the two events triggered by UV irradiation were mechanistically linked.

To investigate the mechanism underlying the different sensitivity of ES cells to UV irradiation, we searched for genes that were differentially regulated in SK-N-MC and LAP-35 cells. Among them, *DHX9* was specifically affected both at gene expression and AS upon UV light irradiation only in SK-N-MC. Interestingly, UV light induced the inclusion of a PTC-containing exon in the *DHX9* transcript (the alternative exon 6A), thus targeting the corresponding transcript to NMD and contributing to *DHX9* down-regulation. As a consequence, in response to UV light treatment, the DHX9 protein is diminished in SK-N-MC but not in LAP-35 cells (Figure 2). This observation is in line with other studies showing that exons sensitive to RNAPII modulation often introduce PTCs that elicit NMD of the spliced mRNAs [14, 33, 38].

Mechanistically, inclusion of exon 6A in *DHX9* pre-mRNA is linked with modulation of RNAPII elongation rate by UV treatment. We found that RNAPII processivity was significantly reduced in SK-N-MC cells but not in LAP-35 cells (Figure 3D), possibly due to the stronger effect of UV light on RNAPII phosphorylation in the former cells. Indeed, strong phosphorylation of RNAPII was previously shown to reduce its elongation rate in cells exposed to genotoxic stress [14]. In line with this concept, we found that treatment of LAP-35 cells with the pTEFB inhibitor DRB artificially affected RNAPII processivity and recapitulated inclusion of the alternative exon 6A of *DHX9* (Figure 3F), reducing DHX9 protein expression (Figure 3G, 3H). Thus, our results prove that factors affecting RNAPII elongation rate (UV irradiation and DRB) regulate *DHX9* AS and expression by controlling the inclusion of an alternative PTC-containing exon in ES cells. Nevertheless, we cannot exclude that UV light irradiation, or etopside treatment, may also induce the expression or activity of some splicing factors, thus contributing to exon 6A inclusion. Given the importance of DHX9 in cancer cells resistance and proliferation, future work will investigate this issue.

DHX9 is a member of the DEXH family of RNA helicases [39], which play important roles in several aspects of RNA metabolism [40]. Homozygous *Dhx9* mutation in mice determines early embryonic lethality [41], suggesting that the *DHX9* gene is essential for viability. Moreover, through its helicase activity DHX9 binds to and resolves mutagenic intra-molecular triplex structures [42, 43], preventing genomic instability and assisting the maintenance of DNA integrity in the replication, recombination, and repair processes. In ES cells, DHX9 forms a complex with EWS-FLI1 and modulates EWS-FLI1-dependent transcription [17]. Thus, DHX9 could be a functional partner for EWS-FLI1 by enhancing engagement of the transcriptional machinery at responsive promoters and inducing local changes in chromatin structure and DNA unwinding. We found that the reduction of DHX9 expression in SK-N-MC cells elicited by treatment with UV light correlates with downregulation of EWS-FLI1 target genes, suggesting that it is physiologically relevant. Moreover, EWS-FLI1 recruitment to the promoter of its target genes is repressed upon UV treatment only in SK-N-MC cells, where *DHX9* expression is downregulated, but not in LAP-35 cells, where DHX9 protein remains high. Consistent with our model is also the observation that *DHX9* knockdown in LAP-35 and in SK-N-MC cells, to similar levels of those obtained by UV irradiation in SK-N-MC cells, is sufficient to repress the expression of *ID2*, *c-MYC* and *CCND1* (Figure 5), regardless of the UV treatment. These results strongly suggest that downregulation of EWS-FLI1 target genes induced by UV light irradiation in SK-N-MC cells is due to reduced expression of DHX9.

The functional interaction between EWS-FLI1 and DHX9 was previously shown to support anchorage-independent growth [17]. In this study, we found that DHX9 expression confers resistance to UV irradiation in ES cells. Indeed, overexpression of recombinant DHX9 makes SK-N-MC cells significantly more resistant to UV light treatment. Conversely, knockdown of endogenous DHX9 renders LAP-35 cells more sensitive to UV light treatment. Since overexpression of DHX9 in HeLa cells did not improve cell survival to UV irradiation, this phenomenon appears to be specific for ES cells. Collectively, these results suggest that DHX9 downregulation could be instrumental to sensitize ES cells to genotoxic stress. In line with this concept, an RNAi screen recently uncovered *DHX9* as a key target gene to sensitize lymphomas to chemotherapeutic treatment [44]. Although DHX9 might play a direct role in DNA repair and genome maintenance through its interaction with the Werner syndrome helicase WRN [45] and BRCA1 [46], our results suggest that it may also participate to this process by enhancing the expression of oncogenic EWS-FLI1 target genes. On the other hand, it is also possible that, in addition to *DHX9,* other genes differentially regulated in the two ES cell lines are involved in the different sensitivity to genotoxic stress. For example, our microarray analysis revealed that *FOS* gene is specifically upregulated in LAP-35 cells but not in SK-N-MC cells. Notably, cells deficient in *c-Fos* are hypersensitive to ultraviolet (UV-C) light and mouse embryonic fibroblasts *fos−/−* are defective in the repair of UV-C induced DNA lesions [47].

ES tumors initially respond well to chemotherapy, but 40% of patients later develop recurrent disease and the majority of them die within 5 years, despite high-dose chemotherapy [48]. Down-regulation of EWS-FLI1 by siRNA approaches resulted in prolonged survival of ES xenograft-bearing mice [49], but this approach currently lacks clinical translation [50]. Recently, a small-molecule targeting EWS-FLI1-DHX9 interaction has been proposed as a strategy to inhibit EWS-FLI1-mediated transcription [35]. Here we provide evidence that genotoxic stresses causing downregulation of DHX9 expression efficiently reduce ES cell growth. Moreover, we have identified a *DHX9* splicing isoform induced by UV light treatment that leads to DHX9 down-regulation in ES cells. Since DHX9-EWS-FLI1 functional interaction represents a good opportunity for clinical intervention, our data indicate a novel strategy for targeting EWS-FLI1 oncogene activity through the modulation of DHX9 expression or activity. In this scenario, the development of antisense oligonucleotides recruiting the spliceosomal complex to the alternative exon 6A in *DHX9* could be instrumental to drive DHX9 downregulation in ES cells and might provide a valuable additional therapy for the treatment of ES.

## MATERIALS AND METHODS

### Cell culture and treatments

SK-N-MC and LAP-35 Ewing Sarcoma cells were a generous gift from Drs F. Moretti and K. Scotlandi, respectively. Both cell lines were cultured in IMDM medium supplemented with 10% fetal bovine serum, penicillin and streptomycin (Gibco), and maintained at 37°C in humidified 5% CO_2_ atmosphere. For treatment, cells were plated at 50%–60% confluence 16 h before UV light irradiation (either 10 or 40 J/m^2^). Fresh medium was immediately added after the treatment and the cells were harvested at different times during the recovery, as indicated. For drug treatment, ES cells were treated 16 hours with DMSO or etoposide, 5-fluorouracile, and cisplatin at different concentration, as indicated.

### Splicing-sensitive microarrays

RNA from three biological replicates of control or UV irradiated Ewing Sarcoma cells (either SK-N-MC or LAP-35) and HEP3B cells collected 6 hours after the treatment was purified using RNeasy Mini kit (Qiagen) and digested with DNase RNase free (Qiagen). cDNA and Cy5-Cy3 labelled cRNA were generated from the total RNA using the Agilent Low RNA Input Fluorescent Linear Amplification kit. 8 μg of each cRNA were used for the hybridization with the arrays (Agilent *In situ* hybridization kit plus). After hybridization, arrays were washed, and scanned images analyzed as previously described [14]. Three biological replicates were hybridized, with both direct and dye-reversal hybridizations. General gene expression values represent the average of log2 ratios for all the probes of a locus. Statistical analyses were carried out with Linear Models for Microarray Data (Limma; Bioconductor Project) [51]. The background correction method used in the analysis was Normexp [52]. Locally weighted linear regression (LOWESS) analysis was used as a normalization method [53].

The cutoff considered was fold change >|1,3| for gene expression changes and fold change >|1,4|; (Z-SCORE >3; *p*-value <0,01) for alternative splicing changes. The data were deposited in GEO database with the accession number GSE59889.

### Transfection experiments

For transfections, cells were plated in 35-mm dishes and transfected with siRNAs (Sigma-Aldrich) at final concentration of 100 nM using Lipofectamine RNAiMax reagent (Invitrogen) according to the manufacturer's instructions. The *DHX9* and *UPF1* siRNA and scrambled sequences are listed in Supplementary Table 2. For over-expression experiments, transfections were performed with 1 μg of appropriate constructs (pEGFP or pEGFP-DHX9) using lipofectamine 2000 (Invitrogen). 48 h after transfections, cells were treated and collected at the indicated time points for RNA or protein analyses.

### Isolation of total RNA and RT-qPCR

Total RNA was extracted by using TriPure Isolation Reagent (Roche) according to the manufacturer's instructions and subjected to DNase digestion (Roche). First-strand cDNA was obtained from 1 μg of RNA using random hexamer and M-MLV-Reverse Transcriptase (Promega, Italy). Synthesized cDNA corresponding to 25 ng total RNA was used for conventional-(GoTaq DNA Polymerase, Promega) or quantitative-PCR (SYBR Green Master Mix for Light-Cycler 480, Roche), according to manufacturer's instructions. Primers used for RT-qPCR are listed in the Supplementary Table 2.

### Protein extraction and western blotting analyses

Total protein extracts were prepared using lysis buffer (100 mM NaCl, 10 mM MgCl_2_, 30 mM Tris-HCl, pH 7.5, 10% glycerol, 1 mM dithiothreitol, 10 mM β-glycerophosphate, 0.5 mM Na_3_VO_4_, and protease inhibitor cocktail (Sigma-Aldrich) supplemented with 0.5% Triton X-100. The extracts were sonicated (1 sec at 30%), incubated on ice for 10 min. and then centrifuged for 10 min at 12,000 g at 4°C. Protein quantification was performed by Quick Start Bradford Protein Assay (Bio-Rad). Cell extracts were diluted in SDS sample buffer and boiled for 5 min. Proteins (10–50 μg) were separated on 6% or 10% SDS-PAGE gels and transferred to Hybond-P membranes (GE Healthcare). Membranes were saturated with 5% non-fat dry milk in phosphate-buffered saline (PBS) containing 0.1% Tween-20 for 1 h at room temperature, and incubated with the following antibodies and dilutions overnight at 4°C: anti-DHX9 1:200 (H300, Santa Cruz Biotechnology), anti-FLI-1 1:200 (sc-356 Santa Cruz Biotechnology), anti-GAPDH 1:1000 (sc-32233 Santa Cruz Biotechnology), anti-RNAPII 1:250 (N20, Santa Cruz Biotechnology), anti-β–ACTIN 1:1000 (MAB1501, Merk Millipore), anti-ERK2 1:1000 (Santa Cruz Biotechnology), and phospho-CHK1 1:1000 (Cell Signaling), anti-UPF1 1:500 (Millipore). Secondary anti-mouse or anti-rabbit IgGs conjugated to horseradish peroxidase (Amersham) were incubated with the membranes for 1 h at room temperature at a 1:10000 dilution in PBS containing 0.1% Tween-20. Immunostained bands were detected by a chemiluminescent method (Santa Cruz Biotechnology). Densitometric analysis was performed by ImageJ software.

### MTS proliferation assay

Cell proliferation was determined using the Cell Titer A96 3-(4,5-dimethylthiazol-2-yl)-5-(3-carboxymethoxyphenyl)-2-(4-sulfophenyl)-2H-tetrazolium, inner salt (MTS) method according to the manufacturer's instructions (Promega) by plating 5 × 10^4^ cells/well in 96-well culture plates.

### Chromatin immunoprecipitation

2 × 10^8^ SK-N-MC and LAP-35 cells were cross-linked with 1% (w/v) formaldehyde (Sigma Aldrich) for 15 min at room temperature, and then formaldehyde was inactivated by the addition of 125 mM glycine (Sigma Aldrich). Cells were washed in cold PBS and lysed to isolate nuclei in a hypotonic buffer containing 5 mM PIPES (pH 8.0), 85 mM KCl, NP40 0.5%, 1 mM dithiothreitol, 10 mM β-glycerophosphate, 0.5 mM Na_3_VO_4_, and protease inhibitor cocktail (Sigma-Aldrich). Isolated nuclei were lysed in a buffer containing 1% SDS, 10 mM EDTA, and 50 mM Tris-HCl (pH 8.0), 1 mM dithiothreitol, 10 mM β-glycerophosphate, 0.5 mM Na_3_VO_4_, and protease inhibitor cocktail (Sigma-Aldrich) and sonicated with Bioruptor (Dyagenode) 2 fold for 6 min (30 sec sonication and 30 sec pause). Chromatin extracts containing DNA fragments (100 μg/sample) with an average size of 200 bp were pre-cleared overnight and then immunoprecipitated for 3 hours using 2 μg of anti-FLI1 (SAB2100822, Sigma Aldrich) or anti-RNAPII (N20, Santa Cruz Biotechnology) or anti RNAPII CTD repeat H14 and H5 (ab24759 and ab24758, Abcam) antibodies and Protein A/G Agarose/Salmon Sperm DNA (Merck Millipore). Precipitated DNA was extracted and analyzed by qPCR using primers listed in Supplementary Table 2.

### Flow cytometry analysis

Percentage of dead cells was determined by staining with propidium iodide (PI 1 μg/ml eBioscience) and acquisition was performed on flow cytometry (Canto, BD Biosciences). Analysis was performed using FlowJo software. In some experiments GFP positive population of SK-N-MC cells transfected with pEGFP and pEGFP-DHX9 constructs, was isolated using a MoFlo high speed cell sorter (Beckman Coulter).

### Colony formation assay

Cell suspensions were plated in 100-mm plates or 35-mm plates at a density of 2000 or 1000 cells/plate, respectively. After one day, cells were treated (UV 10 J/m^2^) and incubated at 37°C in a humidified atmosphere containing 5% CO2 for 12 day, replacing medium every two days. At the end of the incubation period, cells were washed with PBS, fixed in methanol for 10 min at RT and stained 30 min at room temperature with 0.05% Crystal Violet in distilled water on a rotating shaker. After staining, cells were washed twice with tap water and air-dried overnight. The next day clones were counted.

### 5′ splice site score calculation

In order to evaluate the strength of the alternative exon we compared the scores of the 5′ splice sites of the alternative exon 6A versus the downstream constitutive exon 7 of *DHX9* gene. The score expresses how similar the splice sites fit the consensus sequence. A perfect 5′ splice site AAG|gtaagt would have a score of 12.6. According to these parameters provided by Dr. Michael Zhang, CSHL and statistical data calculated by Dr. Tetsushi Yada using the sequence compilation for the GENIE program, we run the Splice Site Score Calculation (http://rulai.cshl.edu/new_alt_exon_db2/HTML/score.html) and we found that the 5′ Splice-Site Score For *DHX9* exon 6A (AAG | GTCAGT) is: 5.9 while the score for the downstream constitutive exon (CAG | GTAAGT) is 12.4. Similar results were obtained using the MaxEntScan::score5ss matrix from the laboratory of Chris Burge (http://genes.mit.edu/burgelab/maxent/Xmaxentscan_scoreseq.html).

## SUPPLEMENTARY MATERIAL FIGURES AND TABLES




