# Supplementary material for: Genotoxic stress inhibits Ewing sarcoma cell growth by modulating alternative pre-mRNA processing of the RNA helicase DHX9
Source: Oncotarget. 2015 Oct 2;6(31):31740–57. doi: 10.18632/oncotarget.5033 (PMC4741636; doi:10.18632/oncotarget.5033)
Supplement: Supplementary file 1 [file oncotarget-06-31740-s001.pdf]

SUPPLEMENTARY FIGURES AND TABLES

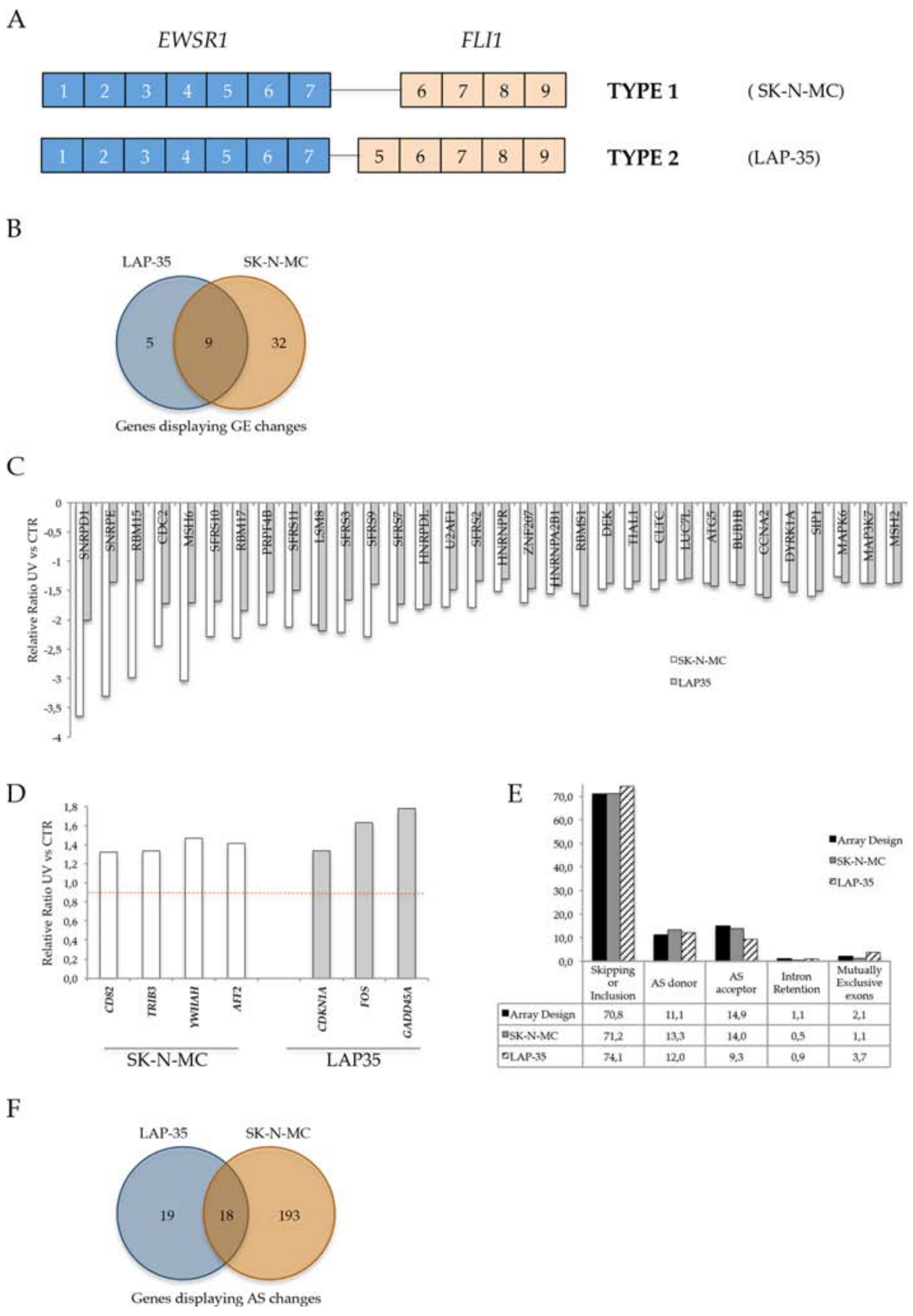

	Skipping or Inclusion	AS donor	AS acceptor	Intron Retention	Mutually Exclusive exons
Array Design	70,8	11,1	14,9	1,1	2,1
SK-N-MC	71,2	13,3	14,0	0,5	1,1
LAP-35	74,1	12,0	9,3	0,9	3,7

Supplementary Figure S1: Splicing sensitive arrays identified gene expression changes upon 10J/m² UV light irradiation. **A.** Scheme of the chromosomal translocation affecting SK-N-MC and LAP-35 ES cells. In the type 1 translocation, reported in SK-N-MC cells, *FLI1* exon 5 is not included in the oncogene, while it is present in the type 2 translocation reported in LAP-35 cells. **B.** Venn diagram shows the overlap of gene expression signatures at gene level induced by 10 J/m² UV light irradiation in SK-N-MC and LAP-35 cells, as indicated, with a threshold of fold change > |2|. **C.** Genes down-regulated at the expression level both in the SK-N-MC and in the LAP-35 cells upon UV light irradiation. **D.** Genes up-regulated in the SK-N-MC and in the LAP-35 cells upon UV light irradiation. **E.** AS categories affected by UV light irradiation either in SK-N-MC or in LAP-35 cells. The fraction of AS events in each of five different AS classes is indicated for all the events monitored in the array (black bars) and for those affected by UV light irradiation in SK-N-MC (gray) and in LAP-35 (hatched). Percentages of each category are shown in the table below the histogram. **F.** Venn diagram shows the overlap of gene expression signatures at AS level induced by 10 J/m² UV light irradiation in SK-N-MC and LAP-35 cells, as indicated, considering a threshold of fold change >|2|.

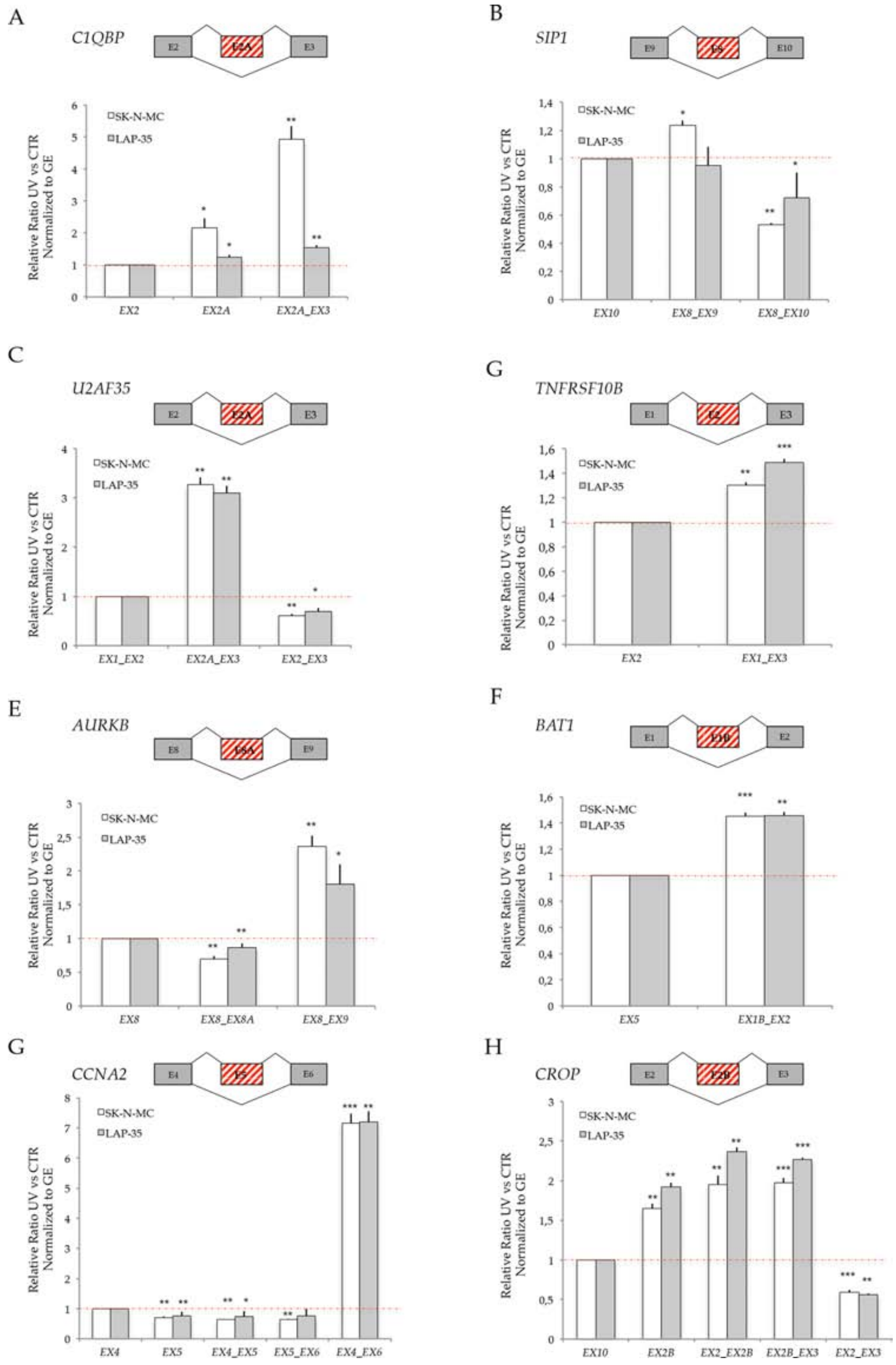

Supplementary Figure S2: RT-qPCR validation of microarray-predicted AS changes in the indicated genes. A. to **H.** AS patterns are schematized in the upper part of each panel. Exon and exon-junction probes used to monitor AS events and to validate the microarray results are indicated. Histograms represent the ratio between qPCR amplification signals characteristic of each isoform, obtained from RNAs isolated from SK-N-MC (white) or LAP-35 (gray bars) cells treated with 10 J/m^2 UV light versus untreated. Ratios of AS amplicons were then normalized to the ratios corresponding to a constitutive exon (GE). Average and standard deviation from three independent biological replicas are shown. *p* values for gene/isoform expression changes are indicated (*$p < 0.05$, **$p < 0.01$, ***$p < 0.001$, statistical analysis was performed by Student's *t*-test).

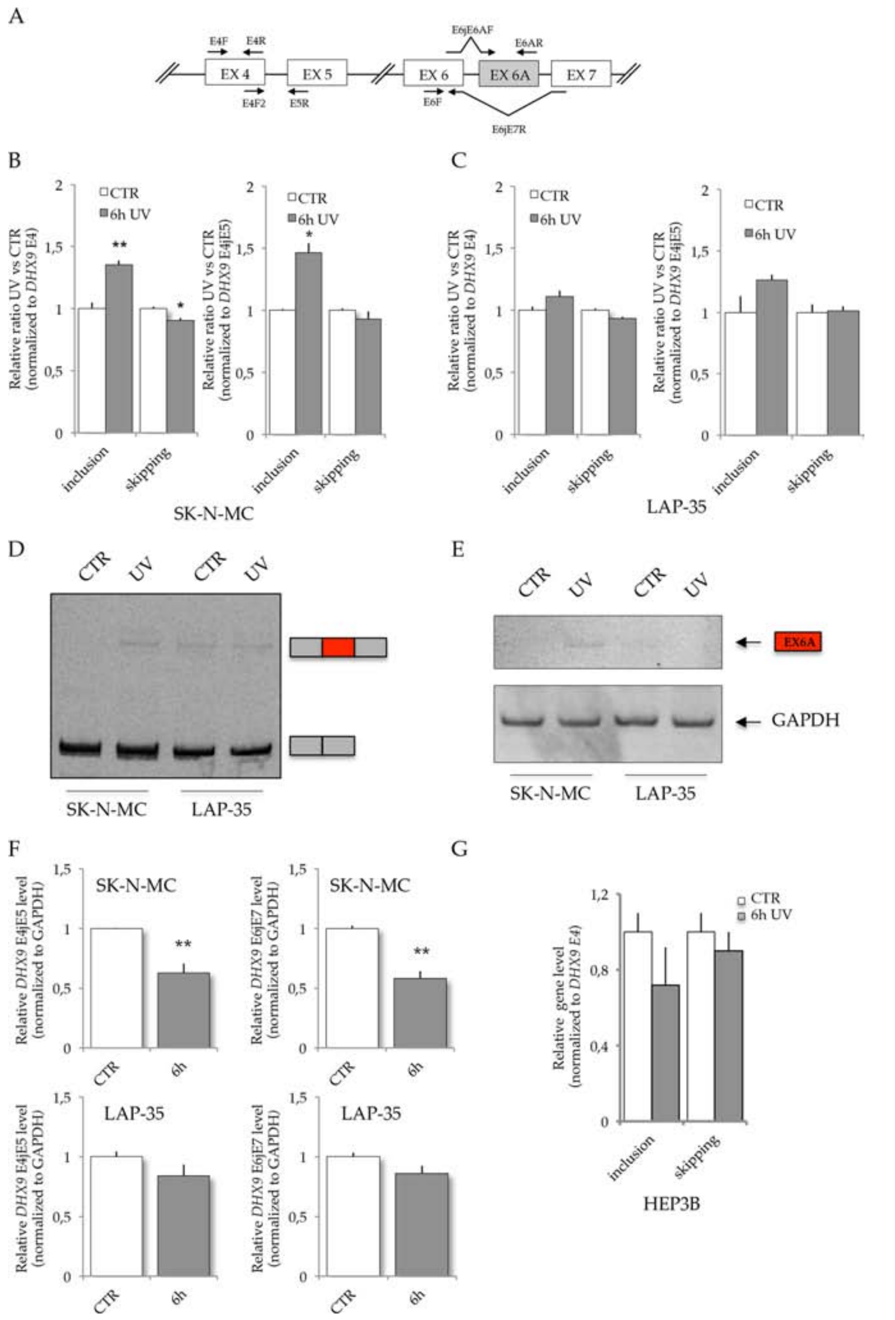

Supplementary Figure S3: UV light irradiation affects *DHX9* mRNA expression and alternative splicing in SK-N-MC cells, but not in LAP-35. A. Scheme of *DHX9* alternative splicing event with the primers used to monitor the event. **B.** and **C.** RT-qPCR analysis of microarray-predicted AS changes in *DHX9* mRNA, normalized to a constitutive exon (exon 4, left panel) or to two consecutive constitutive exons (exon 4 and 5; right panel). Inclusion and skipping of exon 6A are reported both for SK-N-MC (B) and LAP-35 (C) cells. **D.** Standard PCR using primers spanning *DHX9* AS event. Bands corresponding to either inclusion or skipping of exon 6A are indicated both in SK-N-MC and in LAP-35 cells. **E.** Standard PCR using primer pairs to amplify exon 6A (upper panel) and GAPDH (lower panel) both in SK-N-MC and in LAP-35 cells, as indicated. **F.** RT-qPCR analysis of microarray-predicted AS changes in *DHX9* mRNA, using exon junction primers spanning the consecutive exons 4–5 and 6–7. The values are normalized to *GAPDH* gene expression levels for SK-N-MC (upper panels) and LAP-35 cells (lower panels), as indicated. For all the panels, average and standard deviation from three independent biological replicas are shown. *p* values for gene/isoform expression changes are indicated (*$p < 0.05$, **$p < 0.01$, ***$p < 0.001$, statistical analysis was performed by Student's *t*-test). **G.** RT-qPCR analysis of inclusion and skipping of *DHX9* exon 6A upon UV light irradiation in HEP3B cells. The values are normalized to a constitutive exon (exon 4).

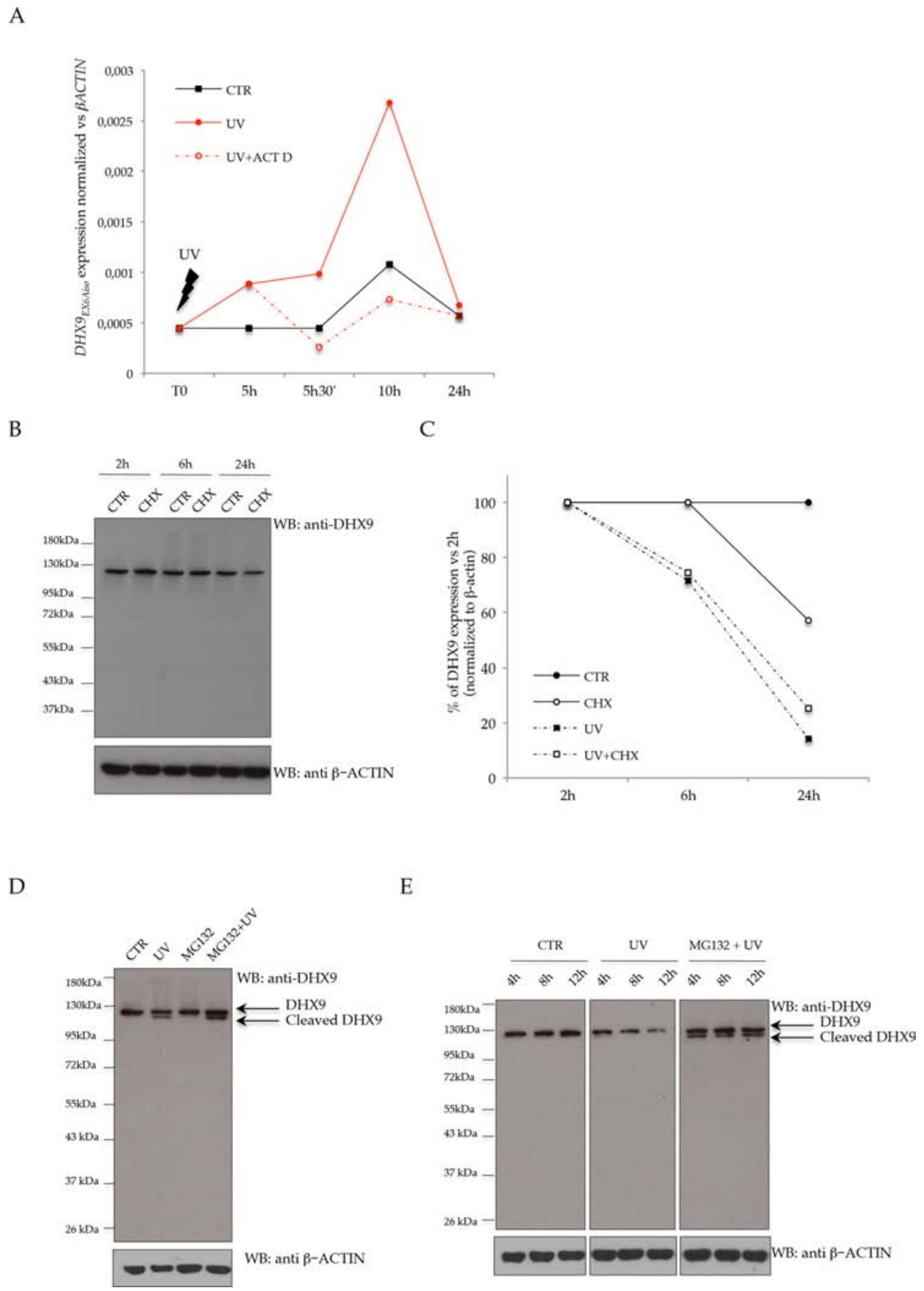

Supplementary Figure S4: DHX9 protein stability and degradation. A. RT-qPCR analysis of SK-N-MC cells irradiated with UV light (10J/m^2) and treated with actinomycin D (10 µg/ml) 5 hours after UV treatment. Cells were harvested at different time points (5 h, 5 h+30′, 10 h, and 24 h) after treatment. Plots represent exon 6A-containing *DHX9* transcript normalized to *β-ACTIN*. **B.** Western blot analysis of total extracts from SK-N-MC harvested at 2, 6 and 24 h of treatment with 10 µg/ml cycloheximide, using antibodies against DHX9 (Santa Cruz Biotechnology) and β-ACTIN (Merk Millipore). 15 µg of total extracts were loaded in each lane. **C.** Quantification of western blot signals from SK-N-MC harvested at 2, 6 and 24 h of treatment with 10 µg/ml cycloheximide, with or without UV light irradiation. The values are calculated as a percentage of DHX9 expression versus the time point at 2 hours. **D.** Western blot analysis of total extracts from SK-N-MC harvested at 4 hours after treatment with 10 µM MG132, with or without UV light irradiation. The PVDF membranes were hybridized with antibodies against DHX9 (Santa Cruz Biotechnology) and β-ACTIN (Merk Millipore). 15 µg of total extracts were loaded in each lane. **E.** Western blot analysis of total extracts from SK-N-MC harvested at 4, 8 and 12 hr of treatment with 10 µM MG132, with or without UV light irradiation. The PVDF membranes were hybridized with antibodies against DHX9 (Santa Cruz Biotechnology) and β-ACTIN (Merk Millipore). 15 µg of total extracts were loaded in each lane.

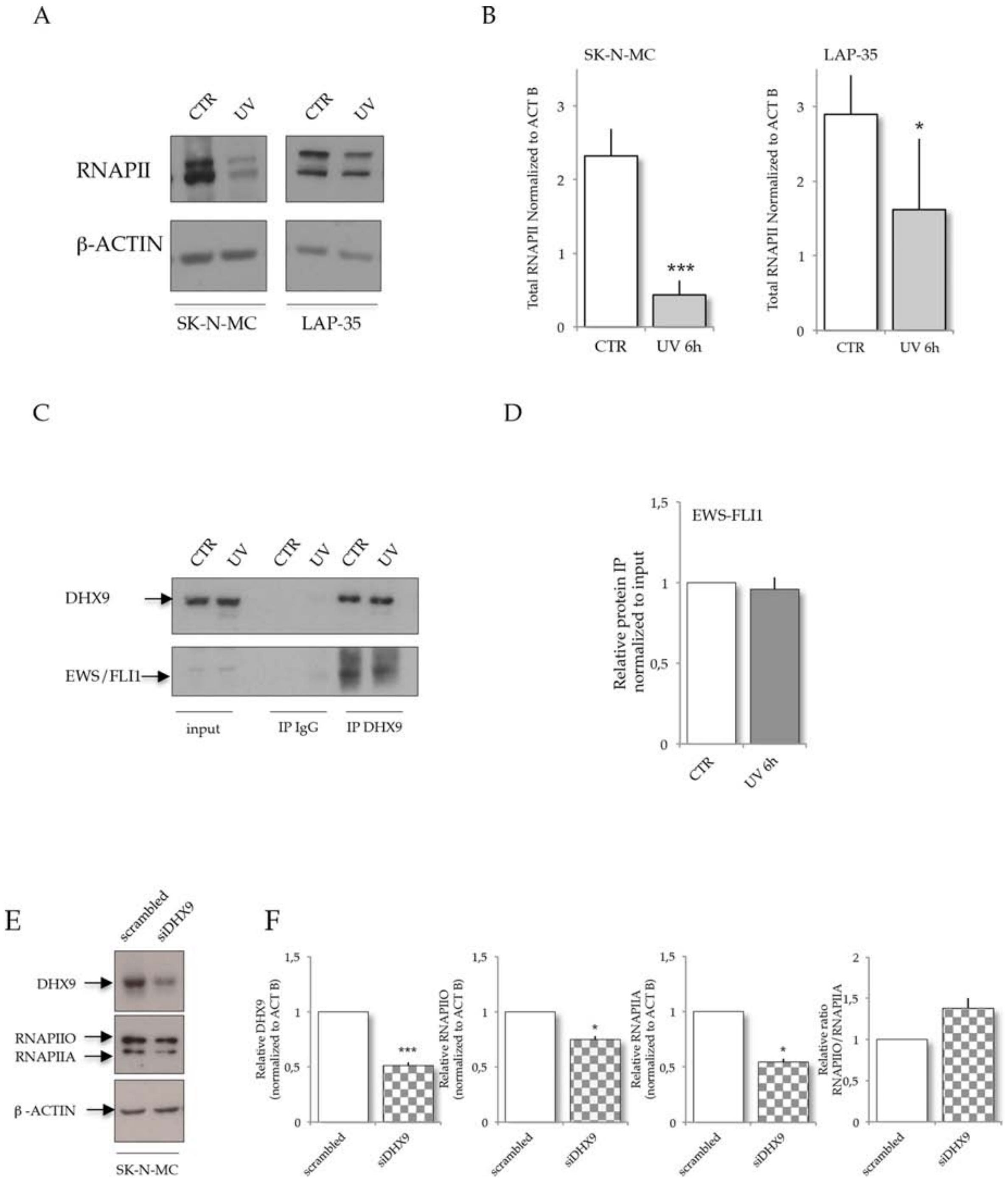

Supplementary Figure S5: RNAPII dynamics in ES cells upon UV light treatment. A. Western blot analysis of total extracts from SK-N-MC and LAP-35 cells irradiated with 10 J/m^2 and harvested 6 hr after treatment, using antibodies against RNAPII (N20, Santa Cruz Biotechnology) and β-ACTIN (Merk Millipore). 20 μg of total ES extracts were loaded in each lane. **B.** Quantification of western blot signals; error bars represent standard deviation of the quantification of three independent experiments. *$p < 0.05$, **$p < 0.01$, ***$p < 0.001$. **C.** DHX9 interaction with EWS-FLI1 was monitored by immunoprecipitation experiments in LAP-35 cells, in normal condition and upon UV light treatment. **D.** Quantification of protein retained on DHX9 containing beads after extensive washes. Relative Protein immunoprecipitated was normalized to input content. **E.** Western blot analysis of DHX9, RNAPII and β—ACTIN expression in extracts (10 μg) from SK-N-MC cells transfected with either scrambled or *siDHX9* oligonucleotides, prepared 72 hr after transfection. **F.** Histograms represent quantification of western blot signals shown in (F) from three independent experiments (mean ± s.d.). In all panels statistical analysis was performed by Student's *t*-test: *$p < 0.05$, **$p < 0.01$, ***$p < 0.001$).

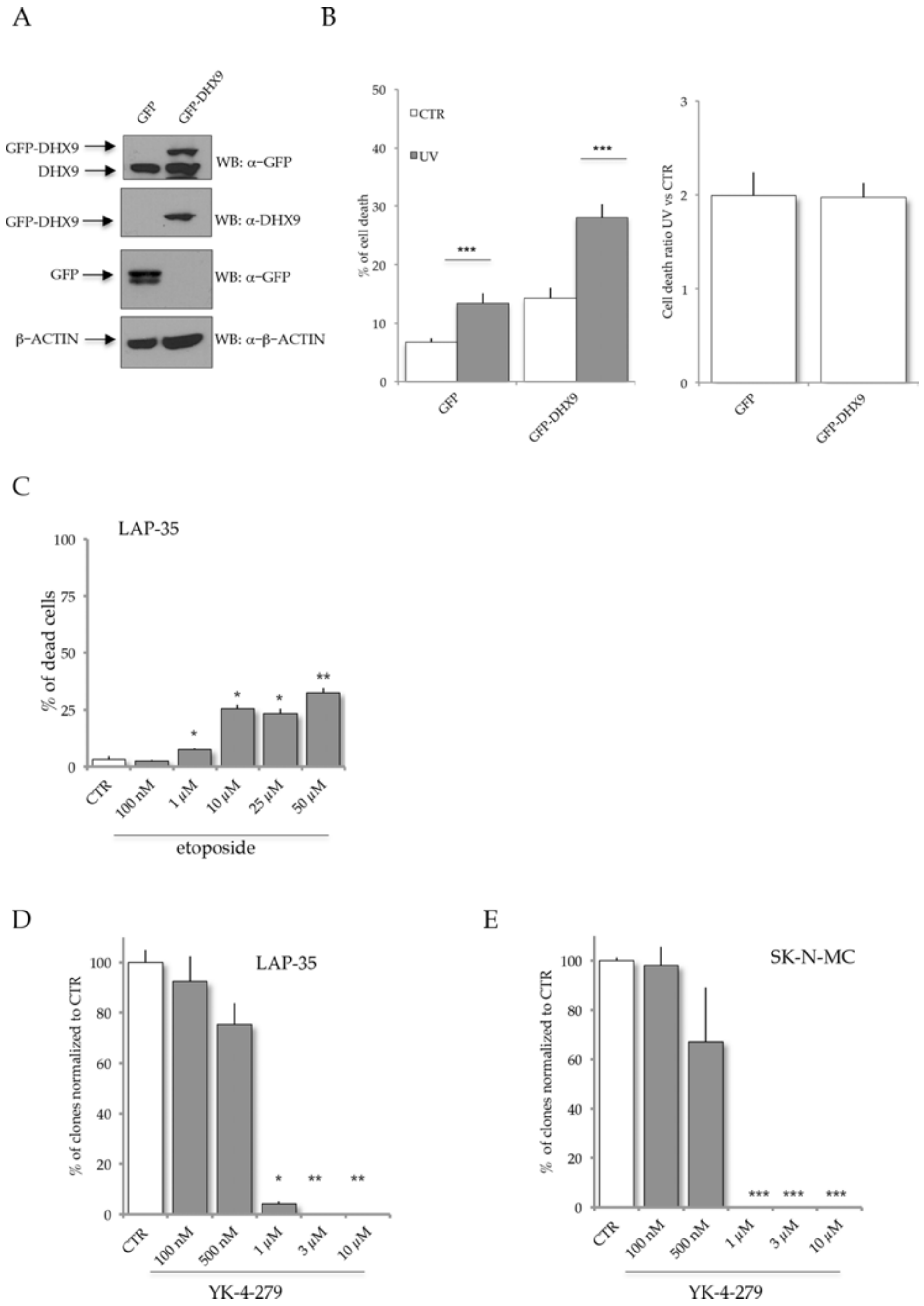

Supplementary Figure S6: DHX9 expression does not affect HeLa resistance to UV light irradiation. Hela cells were transfected with either GFP or GFP-DHX9 cDNAs. 48 hours after transfections cells were treated with 10 J/m^2 UV light and harvested 24 hours after treatment for FACS analysis. **A.** Western blot analysis of GFP, GFP-DHX9 and β—ACTIN expression in HeLa cells at 48 hours after transfection. 10 µg of extracts were loaded in each lane of a 6 or 10% SDS PAGE. **B.** Cell death was detected at 24 hours after the UV treatment by PI staining and flow cytometry analysis; histograms represent the percentage of cell death in GFP-positive CTR (white bars) and UV treated (grey bars) cells. ($n = 3$; mean ± s.d.; *$p < 0.05$, **$p < 0.01$, ***$p < 0.001$ for CTR vs UV). On the right, cell death ratio of CTR and UV in GFP and GFP-DHX9 is shown. **C.** Propidium Iodide (PI) viability assay of LAP-35 cells treated with different concentrations of etoposide for 16 hours. The decrease in viability was expressed as relative percentage of dead LAP-35 cells treated with etoposide (grey bars) versus control (white bars, DMSO). In all panels statistical analysis was performed by Student's *t*-test: *$p < 0.05$, **$p < 0.01$, ***$p < 0.001$. **D.** and **E.** Histograms represent the percentage of colony numbers ($n = 3$; mean ± s.d.) of SK-N-MC (D) and LAP-35 (E) cells treated with different concentration of the inhibitor of EWS-FLI1-DHX9 interaction YK-4–279 (grey bars) versus DMSO treatment (white bars). Statistical analysis was performed by Student's *t*-test: *$p < 0.05$, **$p < 0.01$, ***$p < 0.001$.

Supplementary Table S1: List of gene expression and alternative splicing changes upon 10 J/m^2 UV light irradiation in SK-N-MC and LAP-35 ES cells, and in HEP3B cells.

Supplementary Table S2: List of oligonucleotides used for RT-qPCR microarray validations, ChIP and knockdown experiments

Experiment		Primer name	Sequence
ChIP Experiment		CCND1_ChIP_RT F	**AGGTGTGTTTCTCCCGGTTA**
		CCND1_ChIP_RT R	**CTGCCTTCCTACCTTGACCA**
		TGFBR2_ChIP_RT F	**CAGCGCTGAGTTGAAGTTGA**
		TGFBR2_ChIP_RT R	**AGGGAAGCTGCACAGGAGT**
		ACTB_ChIP_RT F	**TCTGCAGGAGCGTACAGAAC**
		ACTB_ChIP_RT R	**GTGGACATCTCTTGGGCACT**
		Id2_ChIP_RT F	**TGATAGACGTGCCACCTTCC**
		Id2_ChIP_RT R	**GGCTCGGCTCAGAATGAA**
		c-myc_ChIP_RT2 F	**GCAGAGAAAGGGAGAGGGTT**
		c-myc_ChIP_RT2 R	**AACAGAGTAAGAGAGCCGCA**
qPCR	*DHX9*	DHX9_E6AF	**GGAGGAGAATGAGATTGAGTGC**
		DHX9_E6AR	**GCTTTCAGGGGAACAACATC**
		DHX9_E6jE6AF	**GTCCTGATCACAACAGGTGG**
			**with DHX9_E6AR**
		DHX9_E6F	**TTTCAGAAAGAAAAGATCCAAGG**
		DHX9_E6FjE7R	**CATTTCTGCAATAAAGCTCCTG**
		DHX9_E4F	**ATCTCCGCCCCCACTTACT**
		DHX9_E4R	**GAGAGCCAGATGTGGAGGAA**
	UPF1	UPF1_E4F	**AAGAGGTGACCCTGCACAAG**
		UPF1_E4R	**CACCACTGAGTCAGCTTTGG**
	EWS-FLI1	EWS_EX7F	**ATCCTACAGCCAAGCTCCAA**
		FLI1_EX7R	**GGCCGTTGCTCTGTATTCTT**
	c-MYC	c-MYC F	**CTCCACCTCCAGCTTGTACC**
		c-MYC R	**GCTGTCGTTGAGAGGGTAGG**
	CCND1	CCND1 F	**GTGCAAGGCCTGAACCTG**
		CCND1 R	**CGGGTCACACTTGATCACTC**
	TGFBR2	TGFBR2 F	**GAAGATGCTGCTTCTCCAAAG**
		TGFBR2 R	**TGCACTCATCAGAGCTACAGG**
	ID2	Id2 F	**CCCAGAACAAGAAGGTGAGC**
		Id2 R	**ATAGTGGGATGCGAGTCCAG**
	GAPDH	GAPDH F	**ATCACTGCCACCCAGAAGAC**
		GAPDH R	**GGATGCAGGGATGATGTTCT**
	C1QBP	C1QBP_e2A_F	**GAGAGCAGTGGCGTGATCT**
		C1QBP_e2A_R	**AGGCAGGAGAATTGCTTGAA**
		C1QBP_e2A_e3_F	**CTGAGTAGCTGGGATTACAGAATC**

(*Continued*)

Experiment		Primer name	Sequence
		C1QBP_e3_R	**TTGGTGGGATGCTGTTGTTA**
		C1QBP_e2c_F	**CAGAAGCATAAAACCCTCCCTA**
		C1QBP_e2_e3_R	**GAAAGTGACCGTGATTTTTTCC**
		C1QBP_e2c_F	**CAGAAGCATAAAACCCTCCCTA**
		C1QBP_e2c_R	**ATTTCGCTTCTGTCCCATTC**
	CCNA2	CCNA2_e5_B_F	**CCCCCAGAAGTAGCAGAGTTT**
		CCNA2_e5_B_R	**AGGCTGCTGATGCAGAAAGT**
		CCNA2_e4_e5_C_F	**TGCTATGCTGTTAGCCTCAAA**
		CCNA2_e5_B_R	**AGGCTGCTGATGCAGAAAGT**
		CCNA2_e5_e6_D_F	**AAGTTGAAAGTTTAGCAATGTTTTTG**
		CCNA2_e6_R	**CCCGTGACTGTGTAGAGTGC**
		CCNA2_e4_e6_F	**TGCTATGCTGTTAGCCTCTTTTT**
		CCNA2_e6_R	**CCCGTGACTGTGTAGAGTGC**
		CCNA2_e4c_F	**GAATGAGACCCTGCATTTGG**
		CCNA2_e4c_R	**CCTCTCAGCACTGACATGGA**
	SIP1	SIP_e8_F	**ATTCACTGATTCGGCAGCTT**
		SIP_e8_e9_R	**TCTCATCATCTTTGCTATCCACT**
		SIP1_e9_e10_F	**TTAATCTGCTTGGTTAGCAGGTA**
		SIP1_e10C_R	**TTGGCATTGTTTTCCTCAGA**
		SIP1_e8_e10_F	**TGAAGTGAGGCTCTTAGTGGTATTT**
		SIP1_e10C_R	**TTGGCATTGTTTTCCTCAGA**
		SIP1_e10C_F	**TTTAGCTGATGAGCCATCTTGA**
		SIP1_e10C_R	**TTGGCATTGTTTTCCTCAGA**
	U2AF35	140–2A F	**CGTAATCCCCAAAACAGTGC**
		140–3 R	**cgtcagcagactgggaaga**
		140. exon1 Fge	**ATCTTCGGCACCGAGAAAG**
		140 E1.1/C4.1/E4.2 R	**gcaatggtctggctaaacgtc**
		140. exon1 Fge	**ATCTTCGGCACCGAGAAAG**
		140. exon2 Rge	**tggctaaacgtcggtttattg**
	CROP	CROP_e2B_F	**AGAAAAGAGGATTCTTCTCTAGAGAAAG**
		CROP_e2B_R	**CTGCAGAGATCACTTCTCAGG**
		CROP_e2_e2B_F	**CACAAATACACGTTCTGATCTTGA**
		CROP_e2B_R	**CTGCAGAGATCACTTCTCAGG**
		CROP_e2B_F	**AGAAAAGAGGATTCTTCTCTAGAGAAAG**
		CROP_e2B_e3_R	**TTTTTCACACGGACCTGC**
		CROP_e2_F	**TTTTGTCCTGCGGAATTGTT**
		CROP_e2_e3_R	**TTTTTCACACGGACCAAGA**

(*Continued*)

Experiment		Primer name	Sequence
		CROP_ex10C_f	**TCTTGGACTTTGAAAACAGGA**
		CROP_ex10C_b	**TCCAAAGGCTAAGTAGAGCAGA**
	AURKB	AURKB_e8_e8A′_F	**CATGCACAATGAGAAGGTGG**
		AURKB_e8A′_R	**GGCGATAGGTCTCGTTGTGT**
		AURKB_e8_e9_F	**CATGCACAATGAGAAGGTGG**
		AURKB_e9_R	**GACAGATTGAAGGGCAGAGG**
		AURKB_e8_F	**GAGGAAGACAATGTGTGGCA**
		AURKB_e8_R	**ATGCGCCCCTCAATCAT**
	TNFRSF10B	TNFRSF10B_e1_e2_for	**CGGTCCTGCTGTTGGTCT**
		TNFRSF10B_e2_rev	**AGGTGGACACAATCCCTCTG**
		TNFRSF10B_e2_e3c_for	**TCCACCTGGACACCATATCTC**
		TNFRSF10B_e3c_rev	**GCAAGCAGAAAAGGAGGTCA**
		TNFRSF10B_e1_e3c_for	**CGGTCCTGCTGTTGGACA**
		TNFRSF10B_e3c_rev	**GCAAGCAGAAAAGGAGGTCA**
		TNFRSF10B_e2_for	**CTGCTCTGATCACCCAACAA**
		TNFRSF10B_e2_rev	**AGGTGGACACAATCCCTCTG**
	BAT1	BAT1_EX1BF	**tttggagtgccttccaagtg**
		BAT1_EX1B_EX2R	**CATCGTTCTCTGCCATAAcaag**
		BAT1_EX5F	**GAAGGATGAAGAGGTGCTGAA**
		BAT1_EX5R	**TGTGTTTGAGGTTGAGGCTCT**
	siRNA	Scrambled	**5′ GGC AGC AGA GUU CAC UGC U-dCdG**
		siDHX9	**5′-AAG AAG UGC AAG CGA CUC UAG-dCdG**
