# Supplementary material for: Genotoxic stress inhibits Ewing sarcoma cell growth by modulating alternative pre-mRNA processing of the RNA helicase DHX9
Source: Oncotarget. 2015 Oct 2;6(31):31740–57. doi: 10.18632/oncotarget.5033 (PMC4741636; doi:10.18632/oncotarget.5033)
Supplement: Supplementary file 2 [file oncotarget-06-31740-s002.docx]

| **SK-N-MC GE changes** |  |  |  |  |  |  |
| --- | --- | --- | --- | --- | --- | --- |
| **gene name** | **GeneID** | **GE average** | **GE median** | **FC>\|1,3\|** | **Reference RefSeq** |  |
| SmD1_/_SNRPD1 | 146 | -1,89 | -1,87 | **valid GE** | NM_006938 |  |
| SmE1_/_SNRPE | 43 | -1,75 | -1,72 | **valid GE** | NM_003094 |  |
| P14_(CGI110) | 187 | -1,70 | -1,68 | **valid GE** | NM_016047 |  |
| RBM15 | 632_I | -1,67 | -1,58 | **valid GE** | NM_022768 |  |
| CDC2 | G00061 | -1,60 | -1,29 | **valid GE** | NM_001786 |  |
| MSH6 | G00288 | -1,56 | -1,60 | **valid GE** | NM_000179 |  |
| UAP56_(BAT1) | 243 | -1,41 | -1,42 | **valid GE** | NM_080598 |  |
| hnRNP_E2_/_PCBP2 | 232 | -1,36 | -1,32 | **valid GE** | NM_031989 |  |
| tra2_beta_/_SFRS10 | 79 | -1,25 | -1,19 | **valid GE** | NM_004593 |  |
| RNF7 | G00235 | -1,22 | -1,12 | **valid GE** | NM_014245 |  |
| SPF45 | 238 | -1,21 | -1,20 | **valid GE** | NM_032905 |  |
| PABP1 | 30 | -1,18 | -1,24 | **valid GE** | NM_002568 |  |
| PRP4_Kinase_PRPF4B | 67 | -1,14 | -1,06 | **valid GE** | NM_003913 |  |
| SRp54(SFRS11) | 88 | -1,10 | -1,09 | **valid GE** | NM_004768 |  |
| LSM8 | 410_I | -1,07 | -1,06 | **valid GE** | NM_016200 |  |
| SRp20_/_SFRS3 | 39 | -1,05 | -1,15 | **valid GE** | NM_003017 |  |
| NOL5A | 127 | -1,05 | -0,94 | **valid GE** | NM_006392 |  |
| SFRS9 | 700_I | -1,03 | -1,20 | **valid GE** | NM_003769 |  |
| NUDT21 | 536_I | -1,03 | -1,02 | **valid GE** | NM_007006 |  |
| LSM1 | 174 | -1,02 | -1,02 | **valid GE** | NM_014462 |  |
| 9G8_/_SFRS7 | 123 | -0,98 | -1,03 | **valid GE** | NM_006276 |  |
| C1QBP | 73_I | -0,98 | -0,99 | **valid GE** | NM_001212 |  |
| ITGB1 | G00331 | -0,97 | -0,81 | **valid GE** | NM_002211 |  |
| NME1 | G00190 | -0,89 | -0,85 | **valid GE** | NM_000269 |  |
| DFFA | G00089 | -0,89 | -0,89 | **valid GE** | NM_004401 |  |
| SF3A3 | 141 | -0,88 | -0,99 | **valid GE** | NM_006802 |  |
| hnRNP_DL__/_HNRPDL | 231 | -0,88 | -0,86 | **valid GE** | NM_031372 |  |
| AATF | G00002 | -0,86 | -0,73 | **valid GE** | NM_012138 |  |
| SmD2_/_SNRPD2 | 81 | -0,85 | -0,83 | **valid GE** | NM_004597 |  |
| U2AF35_/_U2AF1 | 140 | -0,83 | -0,83 | **valid GE** | NM_006758 |  |
| 15.5_tri-snRNP_/_NHP2L1 | 96 | -0,82 | -0,72 | **valid GE** | NM_005008 |  |
| hnRNP_K | 228 | -0,81 | -0,80 | **valid GE** | NM_031263 |  |
| POLB | G00347 | -0,81 | -0,77 | **valid GE** | NM_002690 |  |
| PABP_4_/_PABPC4 | 62 | -0,79 | -0,77 | **valid GE** | NM_003819 |  |
| DHX9 | 202_I | -0,79 | -0,77 | **valid GE** | NM_001357 |  |
| MAGOH | 25 | -0,79 | -0,78 | **valid GE** | NM_002370 |  |
| SFRS2 | 38 | -0,77 | -0,84 | **valid GE** | NM_003016 |  |
| Sam_68_(KHDRBS1)_ | 131 | -0,75 | -0,75 | **valid GE** | NM_006559 |  |
| PDCD2 | G00205 | -0,74 | -0,80 | **valid GE** | NM_002598 |  |
| hnRNP_R_/_HNRPR | 108 | -0,73 | -0,60 | **valid GE** | NM_001102398 |  |
| STAU | 198 | -0,69 | -0,66 | **valid GE** | NM_017452 |  |
| BUB3 | 87 | -0,69 | -0,66 | **valid GE** | NM_004725 |  |
| CASP6 | G00046 | -0,68 | -0,66 | **valid GE** | NM_001226 |  |
| ZNF207 | 50 | -0,66 | -0,77 | **valid GE** | NM_003457 |  |
| ELAV1 | 237_I | -0,65 | -0,65 | **valid GE** | NM_001419 |  |
| SR140 | 3 | -0,64 | -0,56 | **valid GE** | BQ434974 |  |
| hnRNP_A2/B1 | 227 | -0,64 | -0,64 | **valid GE** | NM_031243 |  |
| NINJ1 | G00189 | -0,64 | -0,63 | **valid GE** | NM_004148 |  |
| PDCD8 | G00209 | -0,63 | -0,59 | **valid GE** | NM_004208 |  |
| RBMS1 | 648_I | -0,63 | -0,63 | **valid GE** | NM_002897 |  |
| hnRNP_I_(PTB) | 33 | -0,62 | -0,61 | **valid GE** | NM_002819 |  |
| FVT1 | G00330 | -0,59 | -0,55 | **valid GE** | NM_002035 |  |
| LMNB1 | G00407 | -0,59 | -0,48 | **valid GE** | NM_005573 |  |
| TFDP2 | G00294 | -0,58 | -0,50 | **valid GE** | NM_000362 |  |
| G10 | 66 | -0,58 | -0,57 | **valid GE** | NM_003910 |  |
| LSM7 | 189 | -0,57 | -0,56 | **valid GE** | NM_016199 |  |
| SIVA | G00057 | -0,56 | -0,55 | **valid GE** | NM_006427 |  |
| DEK | 193_I | -0,56 | -0,56 | **valid GE** | NM_003472 |  |
| hnRNP_M | 114 | -0,56 | -0,37 | **valid GE** | NM_005968 |  |
| TIAL1 | 789_I | -0,56 | -0,55 | **valid GE** | NM_001033925 |  |
| U5-116KD | 73 | -0,55 | -0,58 | **valid GE** | NM_004247 |  |
| CLK1 | 71 | -0,55 | -0,49 | **valid GE** | NM_004071 |  |
| CD164 | G00055 | -0,54 | -0,51 | **valid GE** | NM_006016 |  |
| SNW1 | 737_I | -0,54 | -0,40 | **valid GE** | NM_01224 |  |
| CIDEA | G00077 | -0,53 | -0,56 | **valid GE** | NM_004859 |  |
| PARP1 | G00003 | -0,52 | -0,56 | **valid GE** | NM_001618 |  |
| PAK2 | G00342 | -0,52 | -0,50 | **valid GE** | NM_002577 |  |
| MBNL1 | 419_I | -0,52 | -0,54 | **valid GE** | NM_021038 |  |
| SR89(LUC7L) | 203 | -0,50 | -0,40 | **valid GE** | NM_018032 |  |
| AP5GL | G00008 | -0,50 | -0,46 | **valid GE** | NM_004849 |  |
| RB1CC1 | G00231 | -0,50 | -0,44 | **valid GE** | NM_014781 |  |
| PRP5 | 178 | -0,48 | -0,48 | **valid GE** | NM_014829 |  |
| SFRS14 | 690_I | -0,48 | -0,47 | **valid GE** | NM_014884 |  |
| BAG1 | G00383 | -0,47 | -0,48 | **valid GE** | NM_004323 |  |
| PTK2 | G00225 | -0,46 | -0,43 | **valid GE** | NM_005607 |  |
| BUB1B | G00312 | -0,46 | -0,43 | **valid GE** | NM_001211 |  |
| CCNA2 | G00313 | -0,45 | -0,65 | **valid GE** | NM_001237 |  |
| SSRP1 | 48 | -0,44 | -0,46 | **valid GE** | NM_003146 |  |
| SmB/B'_/_SNRPB | 259 | -0,44 | -0,54 | **valid GE** | NM_198216 |  |
| RBP_7_/_RBM7 | 188 | -0,44 | -0,40 | **valid GE** | NM_016090 |  |
| ASCC3L1 | 37_I | -0,44 | -0,49 | **valid GE** | NM_014014 |  |
| PLP2 | G00346 | -0,44 | -0,37 | **valid GE** | NM_002668 |  |
| DYRK1A | 11 | -0,44 | -0,43 | **valid GE** | NM_001396 |  |
| CHEK2 | G00073 | -0,43 | -0,29 | **valid GE** | NM_001005735 |  |
| RNPS1 | 242 | -0,43 | -0,41 | **valid GE** | NM_080594 |  |
| SIP1 | 54 | -0,43 | -0,68 | **valid GE** | NM_003616 |  |
| BUB1 | G00386 | -0,41 | -0,50 | **valid GE** | NM_004336 |  |
| MAPK6 | G00157 | -0,41 | -0,34 | **valid GE** | NM_002748 |  |
| hnRNP_H3_/_HNRPH3 | 158 | -0,40 | -0,42 | **valid GE** | NM_012207 |  |
| MYBL2 | G00334 | -0,40 | -0,44 | **valid GE** | NM_002466 |  |
| MAP3K7 | G00149 | -0,38 | -0,46 | **valid GE** | NM_003188 |  |
| FMR1 | 273_I | -0,38 | -0,42 | **valid GE** | NM_002024 |  |
| MAPKAPK5 | G00163 | -0,36 | -0,49 | **valid GE** | NM_003668 |  |
| ARMET | G00012 | -0,36 | -0,43 | **valid GE** | NM_006010 |  |
| MSH2 | G00290 | -0,35 | -0,47 | **valid GE** | NM_000251 |  |
| CCNC | G00398 | -0,32 | -0,48 | **valid GE** | NM_005190 |  |
| TSG101 | G00425 | -0,30 | -0,43 | **valid GE** | NM_006292 |  |
| FAF1 | G00434 | -0,30 | -0,48 | **valid GE** | NM_007051 |  |
| KAI1 | G00139 | 0,33 | 0,40 | **valid GE** | NM_002231 |  |
| TRIB3 | G00037 | 0,33 | 0,41 | **valid GE** | NM_021158 |  |
| YWHAH | G00367 | 0,56 | 0,55 | **valid GE** | NM_003405 |  |
| FMR2 | 20 | 0,57 | 0,50 | **valid GE** | NM_002025 |  |
|  |  |  |  |  |  |  |

| **LAP-35 GE changes** |  |  |  |  |  |  |
| --- | --- | --- | --- | --- | --- | --- |
| **gene name** | **GeneID** | **GE average** | **GE median** | **FC>\|1,3\|** | **Reference RefSeq** |  |
| LSM8 | 410_I | -1,14 | -1,13 | **valid GE** | NM_016200 |  |
| SmD1_/_SNRPD1 | 146 | -1,00 | -1,00 | **valid GE** | NM_006938 |  |
| SPF45 | 238 | -0,87 | -0,88 | **valid GE** | NM_032905 |  |
| CDC2 | G00061 | -0,87 | -0,78 | **valid GE** | NM_001786 |  |
| tra2_beta_/_SFRS10 | 79 | -0,83 | -0,74 | **valid GE** | NM_004593 |  |
| RBMS1 | 648_I | -0,83 | -0,81 | **valid GE** | NM_002897 |  |
| SRp20_/_SFRS3 | 39 | -0,81 | -0,73 | **valid GE** | NM_003017 |  |
| hnRNP_DL__/_HNRPDL | 231 | -0,81 | -0,80 | **valid GE** | NM_031372 |  |
| 9G8_/_SFRS7 | 123 | -0,79 | -0,79 | **valid GE** | NM_006276 |  |
| MSH6 | G00288 | -0,77 | -0,77 | **valid GE** | NM_000179 |  |
| PDCD10 | G00203 | -0,75 | -0,66 | **valid GE** | NM_007217 |  |
| CCNA2 | G00313 | -0,73 | -0,70 | **valid GE** | NM_001237 |  |
| Hcc1_(RNPC2) | 258 | -0,69 | -0,68 | **valid GE** | NM_004902 |  |
| AP5GL | G00008 | -0,68 | -0,51 | **valid GE** | NM_004849 |  |
| PRP4_Kinase_PRPF4B | 67 | -0,66 | -0,61 | **valid GE** | NM_003913 |  |
| DYRK1A | 11 | -0,63 | -0,61 | **valid GE** | NM_001396 |  |
| SIP1 | 54 | -0,58 | -0,59 | **valid GE** | NM_003616 |  |
| SRp54(SFRS11) | 88 | -0,58 | -0,58 | **valid GE** | NM_004768 |  |
| U2AF35_/_U2AF1 | 140 | -0,57 | -0,57 | **valid GE** | NM_006758 |  |
| BUB1B | G00312 | -0,52 | -0,49 | **valid GE** | NM_001211 |  |
| SRm160_(SRRM1) | 110 | -0,51 | -0,51 | **valid GE** | NM_005839 |  |
| hnRNP_A2/B1 | 227 | -0,51 | -0,50 | **valid GE** | NM_031243 |  |
| ZNF207 | 50 | -0,50 | -0,55 | **valid GE** | NM_003457 |  |
| U2-A'_/_SNRPA1 | 41 | -0,48 | -0,45 | **valid GE** | NM_003090 |  |
| MAPK6 | G00157 | -0,48 | -0,45 | **valid GE** | NM_002748 |  |
| CDC40 | 116_I | -0,47 | -0,44 | **valid GE** | NM_015891 |  |
| hnRNP_R_/_HNRPR | 108 | -0,47 | -0,38 | **valid GE** | NM_001102398 |  |
| DEK | 193_I | -0,46 | -0,46 | **valid GE** | NM_003472 |  |
| **EWS** | 1009 | -0,45 | -0,45 | **valid GE** | NM_005243 |  |
| SR89(LUC7L) | 203 | -0,45 | -0,37 | **valid GE** | NM_018032 |  |
| SFRS9 | 700_I | -0,44 | -0,48 | **valid GE** | NM_003769 |  |
| RBM15 | 632_I | -0,43 | -0,40 | **valid GE** | NM_022768 |  |
| CHEK1 | G00072 | -0,43 | -0,44 | **valid GE** | NM_001274 |  |
| p68_/_DDX5 | 74 | -0,43 | -0,40 | **valid GE** | NM_004396 |  |
| SWAP_/_SFRS8 | 249 | -0,42 | -0,41 | **valid GE** | NM_152235 |  |
| TIAL1 | 789_I | -0,42 | -0,43 | **valid GE** | NM_001033925 |  |
| MAP3K7 | G00149 | -0,42 | -0,46 | **valid GE** | NM_003188 |  |
| CIDEA | G00077 | -0,41 | -0,40 | **valid GE** | NM_004859 |  |
| SFRS2 | 38 | -0,41 | -0,41 | **valid GE** | NM_003016 |  |
| SmE1_/_SNRPE | 43 | -0,41 | -0,44 | **valid GE** | NM_003094 |  |
| TNFRSF10B | G00263 | -0,40 | -0,38 | **valid GE** | NM_003842 |  |
| DDX26 | G00439 | -0,38 | -0,43 | **valid GE** | NM_012141 |  |
| MSH2 | G00290 | -0,36 | -0,45 | **valid GE** | NM_000251 |  |
| CDKN1A | G00457 | 0,47 | 0,42 | **valid GE** | NM_078467 |  |
| FOS | G00111 | 0,71 | 0,71 | **valid GE** | NM_005252 |  |
| GADD45A | G00114 | 0,83 | 0,83 | **valid GE** | NM_001924 |  |
|  |  |  |  |  |  |  |

| **HEP3B GE changes** |  |  |  |  |  |  |
| --- | --- | --- | --- | --- | --- | --- |
|  |  | **GE** | |  |  |  |
| **gene name** | **GeneID** | **GE average** | **GE median** | **FC>\|1,3\|** | **Reference RefSeq** |  |
| RBM15 | 632_I | -1,5102 | -1,3759 | **valid GE** | NM_022768 |  |
| MCL1 | G00166 | -1,2846 | -1,2846 | **valid GE** | NM_021960 |  |
| PRP4_Kinase_PRPF4B | 67 | -1,2839 | -1,2696 | **valid GE** | NM_003913 |  |
| RBP_7_/_RBM7 | 188 | -1,2407 | -1,1687 | **valid GE** | NM_016090 |  |
| TRAF6 | G00276 | -1,2151 | -1,1358 | **valid GE** | NM_004620 |  |
| PDCD10 | G00203 | -1,2023 | -1,1324 | **valid GE** | NM_007217 |  |
| AP5GL | G00008 | -1,0818 | -0,8767 | **valid GE** | NM_004849 |  |
| CDC2 | G00061 | -1,0178 | -0,9851 | **valid GE** | NM_001786 |  |
| PLK2 | G00427 | -0,9962 | -0,8309 | **valid GE** | NM_006622 |  |
| NFKBIA | G00188 | -0,9937 | -0,9673 | **valid GE** | NM_020529 |  |
| PRP43_/_DDX15 | 10 | -0,9529 | -0,937 | **valid GE** | NM_001358 |  |
| LSM8 | 410_I | -0,9408 | -0,9374 | **valid GE** | NM_016200 |  |
| DYRK1A | 11 | -0,9343 | -0,9314 | **valid GE** | NM_001396 |  |
| CLK4 | 208 | -0,8667 | -0,7779 | **valid GE** | NM_020666 |  |
| DUSP11 | 53 | -0,7999 | -0,7943 | **valid GE** | NM_003584 |  |
| TTK | G00365 | -0,7861 | -0,7617 | **valid GE** | NM_003318 |  |
| MYC | G00335 | -0,777 | -0,7676 | **valid GE** | NM_002467 |  |
| PTPN12 | G00356 | -0,7551 | -0,7456 | **valid GE** | NM_002835 |  |
| MAP2K4 | G00144 | -0,7358 | -0,7012 | **valid GE** | NM_003010 |  |
| MAP4K3 | G00368 | -0,7152 | -0,7116 | **valid GE** | NM_003618 |  |
| p68_/_DDX5 | 74 | -0,699 | -0,6305 | **valid GE** | NM_004396 |  |
| LUCA15_(RBM5) | 105 | -0,6676 | -0,5701 | **valid GE** | NM_005778 |  |
| TGFBR2 | G00259 | -0,6637 | -0,7607 | **valid GE** | NM_003242 |  |
| NFKB1 | G00186 | -0,661 | -0,7054 | **valid GE** | NM_003998 |  |
| SR89(LUC7L) | 203 | -0,6449 | -0,5841 | **valid GE** | NM_018032 |  |
| PIK3CA | G00423 | -0,6435 | -0,6324 | **valid GE** | NM_006218 |  |
| CROP | 165_I | -0,6428 | -0,6236 | **valid GE** | NM_006107 |  |
| CREB1 | G00081 | -0,6416 | -0,6075 | **valid GE** | NM_004379 |  |
| PPM1D | G00220 | -0,6274 | -0,6779 | **valid GE** | NM_003620 |  |
| PTEN | G00292 | -0,623 | -0,5553 | **valid GE** | NM_000314 |  |
| ZNF207 | 50 | -0,6208 | -0,6387 | **valid GE** | NM_003457 |  |
| TIAL1 | 789_I | -0,6094 | -0,601 | **valid GE** | NM_001033925 |  |
| HRAS | G00123 | -0,6076 | -0,4944 | **valid GE** | NM_005343 |  |
| BUB1B | G00312 | -0,5927 | -0,5566 | **valid GE** | NM_001211 |  |
| PPP1R8 | 32 | -0,5921 | -0,5079 | **valid GE** | NM_002713 |  |
| MBNL2 | 420_I | -0,5839 | -0,5519 | **valid GE** | NM_144778 |  |
| RBMS1 | 648_I | -0,5627 | -0,5313 | **valid GE** | NM_002897 |  |
| SWAP_/_SFRS8 | 249 | -0,5587 | -0,5468 | **valid GE** | NM_152235 |  |
| CLK1 | 71 | -0,554 | -0,5285 | **valid GE** | NM_004071 |  |
| SFRS8 | 699_I | -0,5401 | -0,5517 | **valid GE** | NM_004592 |  |
| BCL2L1 | G00024 | -0,5297 | -0,5416 | **valid GE** | NM_001191 |  |
| hnRNP_DL__/_HNRPDL | 231 | -0,5263 | -0,5183 | **valid GE** | NM_031372 |  |
| MSH6 | G00288 | -0,5149 | -0,5352 | **valid GE** | NM_000179 |  |
| TNFAIP3 | G00424 | -0,5076 | -0,4806 | **valid GE** | NM_006290 |  |
| SRp20_/_SFRS3 | 39 | -0,4893 | -0,4945 | **valid GE** | NM_003017 |  |
| MBNL1 | 419_I | -0,486 | -0,4845 | **valid GE** | NM_021038 |  |
| CCNC | G00398 | -0,4841 | -0,5074 | **valid GE** | NM_005190 |  |
| TRIB3 | G00037 | -0,4801 | -0,4779 | **valid GE** | NM_021158 |  |
| SPF45 | 238 | -0,4762 | -0,4703 | **valid GE** | NM_032905 |  |
| 9G8_/_SFRS7 | 123 | -0,4695 | -0,4729 | **valid GE** | NM_006276 |  |
| CCNE1 | G00052 | -0,4694 | -0,488 | **valid GE** | NM_001238 |  |
| SIP1 | 54 | -0,465 | -0,412 | **valid GE** | NM_003616 |  |
| DUSP1 | G00388 | -0,4623 | -0,4553 | **valid GE** | NM_004417 |  |
| MAP4K5 | G00151 | -0,4622 | -0,4084 | **valid GE** | NM_006575 |  |
| SRp54(SFRS11) | 88 | -0,4559 | -0,4538 | **valid GE** | NM_004768 |  |
| Hcc1_(RNPC2) | 258 | -0,4542 | -0,4649 | **valid GE** | NM_004902 |  |
| MALT1 | G00430 | -0,4478 | -0,4266 | **valid GE** | NM_006785 |  |
| KRAS | G00396 | -0,4367 | -0,3826 | **valid GE** | NM_004985 |  |
| CHEK1 | G00072 | -0,4335 | -0,3838 | **valid GE** | NM_001274 |  |
| DDX26 | G00439 | -0,4323 | -0,4458 | **valid GE** | NM_012141 |  |
| CDC40 | 116_I | -0,4244 | -0,4047 | **valid GE** | NM_015891 |  |
| THOC1 | 784_I | -0,4196 | -0,3379 | **valid GE** | NM_005131 |  |
| BRIP1 | G00454 | -0,4051 | -0,3652 | **valid GE** | NM_032043 |  |
| LSM1 | 174 | -0,3984 | -0,4026 | **valid GE** | NM_014462 |  |
| FN1 | 274_I | 0,4459 | 0,449 | **valid GE** | NM_212482 |  |
| GADD45A | G00114 | 0,5347 | 0,5342 | **valid GE** | NM_001924 |  |
|  |  |  |  |  |  |  |

| **SK-N-MC AS changes** | |  |  |  |  |  |
| --- | --- | --- | --- | --- | --- | --- |
| **gene name** | **event** | **GeneID** | **GE average** | **GE median** | **Reference RefSeq** |  |
| AP5GL | alt_splice_acceptor | G00008 | -0,50 | -0,46 | NM_004849 |  |
| AP5GL | novel_exons | G00008 | -0,50 | -0,46 | NM_004849 |  |
| RB1CC1 | novel_exons | G00231 | -0,50 | -0,44 | NM_014781 |  |
| RB1CC1 | alt_splice_acceptor | G00231 | -0,50 | -0,44 | NM_014781 |  |
| RB1CC1 | exon(s)skipped | G00231 | -0,50 | -0,44 | NM_014781 |  |
| PRP5 | novel_exons | 178 | -0,48 | -0,48 | NM_014829 |  |
| SFRS14 | novel_exons | 690_I | -0,48 | -0,47 | NM_014884 |  |
| SFRS14 | novel_exons | 690_I | -0,48 | -0,47 | NM_014884 |  |
| SFRS14 | novel_exons | 690_I | -0,48 | -0,47 | NM_014884 |  |
| BAG1 | novel_exons | G00383 | -0,47 | -0,48 | NM_004323 |  |
| BAG1 | alt_splice_donor | G00383 | -0,47 | -0,48 | NM_004323 |  |
| PTK2 | exon(s)skipped | G00225 | -0,46 | -0,43 | NM_005607 |  |
| PTK2 | exon(s)skipped | G00225 | -0,46 | -0,43 | NM_005607 |  |
| PTK2 | exon(s)skipped | G00225 | -0,46 | -0,43 | NM_005607 |  |
| PTK2 | exon(s)skipped | G00225 | -0,46 | -0,43 | NM_005607 |  |
| PTK2 | alt_splice_donor | G00225 | -0,46 | -0,43 | NM_005607 |  |
| PTK2 | novel_exons | G00225 | -0,46 | -0,43 | NM_005607 |  |
| BUB1B | exon(s)skipped | G00312 | -0,46 | -0,43 | NM_001211 |  |
| BUB1B | exon(s)skipped | G00312 | -0,46 | -0,43 | NM_001211 |  |
| BUB1B | alt_splice_acceptor | G00312 | -0,46 | -0,43 | NM_001211 |  |
| BUB1B | novel_exons | G00312 | -0,46 | -0,43 | NM_001211 |  |
| BUB1B | novel_exons | G00312 | -0,46 | -0,43 | NM_001211 |  |
| SSRP1 | exon(s)skipped | 48 | -0,44 | -0,46 | NM_003146 |  |
| SSRP1 | exon(s)skipped | 48 | -0,44 | -0,46 | NM_003146 |  |
| ASCC3L1 | alt_splice_donor | 37_I | -0,44 | -0,49 | NM_014014 |  |
| PLP2 | alt_splice_donor | G00346 | -0,44 | -0,37 | NM_002668 |  |
| DYRK1A | alt_splice_donor | 11 | -0,44 | -0,43 | NM_001396 |  |
| DYRK1A | alt_splice_donor | 11 | -0,44 | -0,43 | NM_001396 |  |
| DYRK1A | novel_exons | 11 | -0,44 | -0,43 | NM_001396 |  |
| DYRK1A | exon(s)skipped | 11 | -0,44 | -0,43 | NM_001396 |  |
| DYRK1A | exon(s)skipped | 11 | -0,44 | -0,43 | NM_001396 |  |
| **CHEK2** | exon(s)skipped | G00073 | -0,43 | -0,29 | NM_001005735 |  |
| **CHEK2** | novel_exons | G00073 | -0,43 | -0,29 | NM_001005735 |  |
| **CHEK2** | exon(s)skipped | G00073 | -0,43 | -0,29 | NM_001005735 |  |
| **CHEK2** | exon(s)skipped | G00073 | -0,43 | -0,29 | NM_001005735 |  |
| **CHEK2** | alt_splice_donor | G00073 | -0,43 | -0,29 | NM_001005735 |  |
| **CHEK2** | alt_splice_acceptor | G00073 | -0,43 | -0,29 | NM_001005735 |  |
| **CHEK2** | exon(s)skipped | G00073 | -0,43 | -0,29 | NM_001005735 |  |
| **CHEK2** | novel_exons | G00073 | -0,43 | -0,29 | NM_001005735 |  |
| **CHEK2** | exon(s)skipped | G00073 | -0,43 | -0,29 | NM_001005735 |  |
| RNPS1 | exon(s)skipped | 242 | -0,43 | -0,41 | NM_080594 |  |
| RNPS1 | exon(s)skipped | 242 | -0,43 | -0,41 | NM_080594 |  |
| RNPS1 | exon(s)skipped | 242 | -0,43 | -0,41 | NM_080594 |  |
| RNPS1 | novel_exons | 242 | -0,43 | -0,41 | NM_080594 |  |
| RNPS1 | novel_exons | 242 | -0,43 | -0,41 | NM_080594 |  |
| RNPS1 | novel_exons | 242 | -0,43 | -0,41 | NM_080594 |  |
| RNPS1 | novel_exons | 242 | -0,43 | -0,41 | NM_080594 |  |
| BUB1 | alt_splice_acceptor | G00386 | -0,41 | -0,50 | NM_004336 |  |
| BUB1 | alt_splice_acceptor | G00386 | -0,41 | -0,50 | NM_004336 |  |
| MAPK6 | novel_exons | G00157 | -0,41 | -0,34 | NM_002748 |  |
| hnRNP_H3_/_HNRPH3 | exon(s)skipped | 158 | -0,40 | -0,42 | NM_012207 |  |
| hnRNP_H3_/_HNRPH3 | exon(s)skipped | 158 | -0,40 | -0,42 | NM_012207 |  |
| hnRNP_H3_/_HNRPH3 | intron_retained | 158 | -0,40 | -0,42 | NM_012207 |  |
| hnRNP_H3_/_HNRPH3 | exon(s)skipped | 158 | -0,40 | -0,42 | NM_012207 |  |
| hnRNP_H3_/_HNRPH3 | exon(s)skipped | 158 | -0,40 | -0,42 | NM_012207 |  |
| hnRNP_H3_/_HNRPH3 | exon(s)skipped | 158 | -0,40 | -0,42 | NM_012207 |  |
| hnRNP_H3_/_HNRPH3 | exon(s)skipped | 158 | -0,40 | -0,42 | NM_012207 |  |
| hnRNP_H3_/_HNRPH3 | alt_splice_donor | 158 | -0,40 | -0,42 | NM_012207 |  |
| hnRNP_H3_/_HNRPH3 | alt_splice_donor | 158 | -0,40 | -0,42 | NM_012207 |  |
| hnRNP_H3_/_HNRPH3 | alt_splice_donor | 158 | -0,40 | -0,42 | NM_012207 |  |
| MYBL2 | exon(s)skipped | G00334 | -0,40 | -0,44 | NM_002466 |  |
| MYBL2 | alt_splice_donor | G00334 | -0,40 | -0,44 | NM_002466 |  |
| MYBL2 | alt_splice_donor | G00334 | -0,40 | -0,44 | NM_002466 |  |
| MYBL2 | alt_splice_acceptor | G00334 | -0,40 | -0,44 | NM_002466 |  |
| MYBL2 | exon(s)skipped | G00334 | -0,40 | -0,44 | NM_002466 |  |
| MYBL2 | exon(s)skipped | G00334 | -0,40 | -0,44 | NM_002466 |  |
| MYBL2 | alt_splice_acceptor | G00334 | -0,40 | -0,44 | NM_002466 |  |
| HPRP3 | novel_exons | 85 | -0,39 | -0,39 | NM_004698 |  |
| MAP3K7 | novel_exons | G00149 | -0,38 | -0,46 | NM_003188 |  |
| DAXX | exon(s)skipped | G00087 | -0,38 | -0,39 | NM_001350 |  |
| DAXX | alt_splice_acceptor | G00087 | -0,38 | -0,39 | NM_001350 |  |
| DAXX | alt_splice_acceptor | G00087 | -0,38 | -0,39 | NM_001350 |  |
| DAXX | alt_splice_acceptor | G00087 | -0,38 | -0,39 | NM_001350 |  |
| GRIM19 | alt_splice_acceptor | G00117 | -0,37 | -0,36 | NM_015965 |  |
| BCL6 | alt_splice_acceptor | G00027 | -0,37 | -0,26 | NM_001706 |  |
| MAPKAPK5 | exon(s)skipped | G00163 | -0,36 | -0,49 | NM_003668 |  |
| MAPKAPK5 | novel_exons | G00163 | -0,36 | -0,49 | NM_003668 |  |
| ARMET | novel_exons | G00012 | -0,36 | -0,43 | NM_006010 |  |
| WDR57 | Mutually-Exclusive-Exons | 858_I | -0,36 | -0,39 | NM_004814 |  |
| PPP2R1B | exon(s)skipped | G00221 | -0,35 | -0,25 | NM_002716 |  |
| PPP2R1B | exon(s)skipped | G00221 | -0,35 | -0,25 | NM_002716 |  |
| PSIP1 | novel_exons | 240 | -0,35 | -0,27 | NM_033222 |  |
| PSIP1 | intron_retained | 240 | -0,35 | -0,27 | NM_033222 |  |
| SUPT16H | alt_splice_acceptor | 762_I | -0,35 | -0,35 | NM_007192 |  |
| SUPT16H | novel_exons | 762_I | -0,35 | -0,35 | NM_007192 |  |
| PTPN11 | alt_splice_donor | G00355 | -0,35 | -0,32 | NM_002834 |  |
| MSH2 | alt_splice_donor | G00290 | -0,35 | -0,47 | NM_000251 |  |
| CHEK1 | exon(s)skipped | G00072 | -0,35 | -0,30 | NM_001274 |  |
| CHEK1 | alt_splice_donor | G00072 | -0,35 | -0,30 | NM_001274 |  |
| MAPK14 | novel_exons | G00155 | -0,35 | -0,25 | NM_001315 |  |
| MAPK14 | novel_exons | G00155 | -0,35 | -0,25 | NM_001315 |  |
| SFRS12 | alt_splice_acceptor | 246 | -0,33 | -0,23 | NM_139168 |  |
| TRAF6 | exon(s)skipped | G00276 | -0,33 | -0,31 | NM_004620 |  |
| CCNC | alt_splice_donor | G00398 | -0,32 | -0,48 | NM_005190 |  |
| ACVR1B | exon(s)skipped | G00380 | -0,32 | -0,21 | NM_004302 |  |
| MKI67 | exon(s)skipped | G00333 | -0,32 | -0,27 | NM_002417 |  |
| SWAP_/_SFRS8 | exon(s)skipped | 249 | -0,32 | -0,27 | NM_152235 |  |
| LUCA15_(RBM5) | exon(s)skipped | 105 | -0,32 | -0,32 | NM_005778 |  |
| LUCA15_(RBM5) | exon(s)skipped | 105 | -0,32 | -0,32 | NM_005778 |  |
| MCL1 | exon(s)skipped | G00166 | -0,31 | -0,31 | NM_021960 |  |
| PPP1R8 | alt_splice_donor | 32 | -0,31 | -0,26 | NM_002713 |  |
| PUF60 | alt_splice_acceptor | 241 | -0,31 | -0,34 | NM_078480 |  |
| PUF60 | novel_exons | 241 | -0,31 | -0,34 | NM_078480 |  |
| PUF60 | novel_exons | 241 | -0,31 | -0,34 | NM_078480 |  |
| PUF60 | novel_exons | 241 | -0,31 | -0,34 | NM_078480 |  |
| FXR1 | exon(s)skipped | 97 | -0,31 | -0,30 | NM_005087 |  |
| FXR1 | novel_exons | 97 | -0,31 | -0,30 | NM_005087 |  |
| RAMP | exon(s)skipped | G00230 | -0,30 | -0,27 | NM_016448 |  |
| RAMP | exon(s)skipped | G00230 | -0,30 | -0,27 | NM_016448 |  |
| RAMP | exon(s)skipped | G00230 | -0,30 | -0,27 | NM_016448 |  |
| RAMP | exon(s)skipped | G00230 | -0,30 | -0,27 | NM_016448 |  |
| MAPK7 | alt_splice_donor | G00158 | -0,30 | -0,29 | NM_002749 |  |
| MAPK7 | alt_splice_donor | G00158 | -0,30 | -0,29 | NM_002749 |  |
| TSG101 | exon(s)skipped | G00425 | -0,30 | -0,43 | NM_006292 |  |
| TSG101 | novel_exons | G00425 | -0,30 | -0,43 | NM_006292 |  |
| SF3B3 | exon(s)skipped | 681_I | -0,30 | -0,34 | NM_012426 |  |
| SF3B3 | exon(s)skipped | 681_I | -0,30 | -0,34 | NM_012426 |  |
| FAF1 | exon(s)skipped | G00434 | -0,30 | -0,48 | NM_007051 |  |
| PICALM | exon(s)skipped | G00212 | -0,28 | -0,26 | NM_001008660 |  |
| CLK4 | novel_exons | 208 | -0,27 | -0,18 | NM_020666 |  |
| CLK4 | novel_exons | 208 | -0,27 | -0,18 | NM_020666 |  |
| CLK4 | novel_exons | 208 | -0,27 | -0,18 | NM_020666 |  |
| DDX54 | exon(s)skipped | G00088 | -0,26 | -0,27 | NM_024072 |  |
| DDX54 | alt_splice_donor | G00088 | -0,26 | -0,27 | NM_024072 |  |
| ATIC | exon(s)skipped | G00014 | -0,26 | -0,33 | NM_004044 |  |
| ATIC | alt_splice_donor | G00014 | -0,26 | -0,33 | NM_004044 |  |
| ATIC | exon(s)skipped | G00014 | -0,26 | -0,33 | NM_004044 |  |
| ATIC | alt_splice_donor | G00014 | -0,26 | -0,33 | NM_004044 |  |
| ATIC | exon(s)skipped | G00014 | -0,26 | -0,33 | NM_004044 |  |
| CUG-BP | alt_splice_acceptor | 132 | -0,26 | -0,16 | NM_006560 |  |
| CUG-BP | novel_exons | 132 | -0,26 | -0,16 | NM_006560 |  |
| CUG-BP | novel_exons | 132 | -0,26 | -0,16 | NM_006560 |  |
| CUG-BP | novel_exons | 132 | -0,26 | -0,16 | NM_006560 |  |
| ELAC2 | exon(s)skipped | G00097 | -0,25 | -0,21 | NM_018127 |  |
| ELAC2 | exon(s)skipped | G00097 | -0,25 | -0,21 | NM_018127 |  |
| ELAC2 | exon(s)skipped | G00097 | -0,25 | -0,21 | NM_018127 |  |
| ELAC2 | exon(s)skipped | G00097 | -0,25 | -0,21 | NM_018127 |  |
| ELAC2 | exon(s)skipped | G00097 | -0,25 | -0,21 | NM_018127 |  |
| ELAC2 | exon(s)skipped | G00097 | -0,25 | -0,21 | NM_018127 |  |
| ELAC2 | alt_splice_donor | G00097 | -0,25 | -0,21 | NM_018127 |  |
| SRp75_/_SFRS4 | exon(s)skipped | 104 | -0,25 | -0,29 | NM_005626 |  |
| SRp75_/_SFRS4 | exon(s)skipped | 104 | -0,25 | -0,29 | NM_005626 |  |
| SRp75_/_SFRS4 | novel_exons | 104 | -0,25 | -0,29 | NM_005626 |  |
| MAP2K4 | exon(s)skipped | G00144 | -0,25 | -0,25 | NM_003010 |  |
| RAD51 | exon(s)skipped | G00227 | -0,25 | -0,22 | NM_002875 |  |
| RAD51 | alt_splice_acceptor | G00227 | -0,25 | -0,22 | NM_002875 |  |
| MAP4K4 | exon(s)skipped | G00464 | -0,25 | -0,28 | NM_145686 |  |
| MAP4K4 | exon(s)skipped | G00464 | -0,25 | -0,28 | NM_145686 |  |
| MAP4K4 | exon(s)skipped | G00464 | -0,25 | -0,28 | NM_145686 |  |
| MAP4K4 | novel_exons | G00464 | -0,25 | -0,28 | NM_145686 |  |
| MAP4K4 | exon(s)skipped | G00464 | -0,25 | -0,28 | NM_145686 |  |
| MAP4K4 | exon(s)skipped | G00464 | -0,25 | -0,28 | NM_145686 |  |
| PPIL3b_/_PPIL3 | novel_exons | 236 | -0,24 | -0,24 | NM_032472 |  |
| PPIL3b_/_PPIL3 | novel_exons | 236 | -0,24 | -0,24 | NM_032472 |  |
| PPIL3b_/_PPIL3 | novel_exons | 236 | -0,24 | -0,24 | NM_032472 |  |
| PPIL3b_/_PPIL3 | novel_exons | 236 | -0,24 | -0,24 | NM_032472 |  |
| PPIL3b_/_PPIL3 | novel_exons | 236 | -0,24 | -0,24 | NM_032472 |  |
| PPIL3b_/_PPIL3 | novel_exons | 236 | -0,24 | -0,24 | NM_032472 |  |
| PPIL3b_/_PPIL3 | exon(s)skipped | 236 | -0,24 | -0,24 | NM_032472 |  |
| MAP3K5 | exon(s)skipped | G00413 | -0,24 | -0,26 | NM_005923 |  |
| SF3A1 | alt_splice_acceptor | 113 | -0,23 | -0,21 | NM_005877 |  |
| CDK9 | novel_exons | 1015 | -0,23 | -0,25 | NM_001261 |  |
| CFLAR | exon(s)skipped | G00071 | -0,21 | -0,29 | NM_003879 |  |
| CFLAR | exon(s)skipped | G00071 | -0,21 | -0,29 | NM_003879 |  |
| CROP | Mutually-Exclusive-Exons | 165_I | -0,21 | -0,19 | NM_006107 |  |
| CROP | novel_exons | 165_I | -0,21 | -0,19 | NM_006107 |  |
| E2F4 | alt_splice_donor | G00096 | -0,21 | -0,26 | NM_001950 |  |
| E2F4 | alt_splice_donor | G00096 | -0,21 | -0,26 | NM_001950 |  |
| E2F4 | alt_splice_acceptor | G00096 | -0,21 | -0,26 | NM_001950 |  |
| E2F4 | exon(s)skipped | G00096 | -0,21 | -0,26 | NM_001950 |  |
| PRPF31 | alt_splice_donor | 601_I | -0,21 | -0,21 | NM_01562 |  |
| SF1 | alt_splice_donor | 82 | -0,20 | -0,17 | NM_004630 |  |
| FGFR1 | exon(s)skipped | G00106 | -0,20 | -0,29 | NM_000604 |  |
| SLM2_/KHDRBS3 | exon(s)skipped | 130 | -0,20 | -0,07 | NM_006558 |  |
| RAF1 | novel_exons | G00359 | -0,20 | -0,21 | NM_002880 |  |
| RAF1 | exon(s)skipped | G00359 | -0,20 | -0,21 | NM_002880 |  |
| LMNA | exon(s)skipped | G00141 | -0,19 | -0,22 | NM_005572 |  |
| tra2_alpha | novel_exons | 167 | -0,19 | -0,19 | NM_013293 |  |
| tra2_alpha | novel_exons | 167 | -0,19 | -0,19 | NM_013293 |  |
| tra2_alpha | novel_exons | 167 | -0,19 | -0,19 | NM_013293 |  |
| tra2_alpha | novel_exons | 167 | -0,19 | -0,19 | NM_013293 |  |
| TNFRSF10B | exon(s)skipped | G00263 | -0,19 | -0,32 | NM_003842 |  |
| TNPO2 | alt_splice_acceptor | G00443 | -0,19 | -0,13 | NM_013433 |  |
| TNPO2 | novel_exons | G00443 | -0,19 | -0,13 | NM_013433 |  |
| TNPO2 | novel_exons | G00443 | -0,19 | -0,13 | NM_013433 |  |
| SRP54 | Mutually-Exclusive-Exons | 751_I | -0,19 | -0,18 | NM_003136 |  |
| FXR2 | novel_exons | 92 | -0,18 | -0,20 | NM_004860 |  |
| CDKN2A | alt_splice_acceptor | G00070 | -0,18 | -0,16 | NM_000077 |  |
| CDKN2A | alt_splice_donor | G00070 | -0,18 | -0,16 | NM_000077 |  |
| EP300 | exon(s)skipped | G00318 | -0,17 | -0,07 | NM_001429 |  |
| ZNF198 | exon(s)skipped | G00279 | -0,17 | -0,20 | NM_003453 |  |
| ZNF198 | exon(s)skipped | G00279 | -0,17 | -0,20 | NM_003453 |  |
| DNCH1 | alt_splice_donor | G00093 | -0,17 | -0,15 | NM_001376 |  |
| DNCH1 | alt_splice_acceptor | G00093 | -0,17 | -0,15 | NM_001376 |  |
| DNCH1 | alt_splice_acceptor | G00093 | -0,17 | -0,15 | NM_001376 |  |
| DNCH1 | exon(s)skipped | G00093 | -0,17 | -0,15 | NM_001376 |  |
| CDC7 | alt_splice_acceptor | G00066 | -0,17 | -0,29 | NM_003503 |  |
| STAT3 | exon(s)skipped | G00249 | -0,17 | -0,17 | NM_003150 |  |
| STAT3 | alt_splice_acceptor | G00249 | -0,17 | -0,17 | NM_003150 |  |
| MAP3K4 | novel_exons | G00429 | -0,17 | -0,10 | NM_006724 |  |
| EMD | alt_splice_acceptor | G00100 | -0,17 | -0,15 | NM_000117 |  |
| EMD | alt_splice_donor | G00100 | -0,17 | -0,15 | NM_000117 |  |
| CCNT2 | alt_splice_donor | 7 | -0,17 | -0,17 | NM_001241 |  |
| BCL7A | alt_splice_donor | G00450 | -0,16 | -0,14 | NM_020993 |  |
| SF4 | alt_splice_acceptor | 684_I | -0,16 | -0,21 | NM_182812 |  |
| SF4 | alt_splice_acceptor | 684_I | -0,16 | -0,21 | NM_182812 |  |
| RBM6 | exon(s)skipped | 644_I | -0,15 | -0,15 | NM_005777 |  |
| RBM6 | exon(s)skipped | 644_I | -0,15 | -0,15 | NM_005777 |  |
| RBM6 | Mutually-Exclusive-Exons | 644_I | -0,15 | -0,15 | NM_005777 |  |
| RBM6 | exon(s)skipped | 644_I | -0,15 | -0,15 | NM_005777 |  |
| MYH9 | alt_splice_acceptor | G00336 | -0,15 | -0,15 | NM_002473 |  |
| ABL1 | alt_splice_donor | G00436 | -0,14 | -0,14 | NM_007313 |  |
| BAX | exon(s)skipped | G00020 | -0,14 | -0,16 | NM_004324 |  |
| BAX | exon(s)skipped | G00020 | -0,14 | -0,16 | NM_004324 |  |
| ATR | exon(s)skipped | G00310 | -0,14 | -0,18 | NM_001184 |  |
| TFE3(CA150) | exon(s)skipped | 139 | -0,14 | -0,11 | NM_006706 |  |
| TFE3(CA150) | exon(s)skipped | 139 | -0,14 | -0,11 | NM_006706 |  |
| TFE3(CA150) | novel_exons | 139 | -0,14 | -0,11 | NM_006706 |  |
| RELA | alt_splice_acceptor | G00234 | -0,13 | -0,14 | NM_021975 |  |
| MKNK2 | alt_splice_donor | G00172 | -0,13 | -0,17 | NM_017572 |  |
| MKNK2 | novel_exons | G00172 | -0,13 | -0,17 | NM_017572 |  |
| MKNK2 | alt_splice_acceptor | G00172 | -0,13 | -0,17 | NM_017572 |  |
| RAD54L | alt_splice_donor | G00228 | -0,13 | -0,10 | NM_003579 |  |
| RAD54L | exon(s)skipped | G00228 | -0,13 | -0,10 | NM_003579 |  |
| RAD54L | alt_splice_donor | G00228 | -0,13 | -0,10 | NM_003579 |  |
| PDCD6IP | novel_exons | G00208 | -0,13 | -0,10 | NM_013374 |  |
| PDCD6IP | exon(s)skipped | G00208 | -0,13 | -0,10 | NM_013374 |  |
| PDCD6IP | novel_exons | G00208 | -0,13 | -0,10 | NM_013374 |  |
| CypE_/_PPIE | alt_splice_acceptor | 117 | -0,13 | -0,13 | NM_006112 |  |
| CypE_/_PPIE | alt_splice_acceptor | 117 | -0,13 | -0,13 | NM_006112 |  |
| CypE_/_PPIE | novel_exons | 117 | -0,13 | -0,13 | NM_006112 |  |
| NF1 | exon(s)skipped | G00291 | -0,13 | -0,07 | NM_000267 |  |
| LSM4 | novel_exons | 163 | -0,13 | -0,15 | NM_012321 |  |
| MLH1 | alt_splice_acceptor | G00289 | -0,12 | -0,20 | NM_000249 |  |
| MLH1 | novel_exons | G00289 | -0,12 | -0,20 | NM_000249 |  |
| MLH1 | exon(s)skipped | G00289 | -0,12 | -0,20 | NM_000249 |  |
| IKBKAP | exon(s)skipped | G00125 | -0,12 | -0,14 | NM_003640 |  |
| IKBKAP | alt_splice_acceptor | G00125 | -0,12 | -0,14 | NM_003640 |  |
| IKBKAP | alt_splice_donor | G00125 | -0,12 | -0,14 | NM_003640 |  |
| IKBKAP | exon(s)skipped | G00125 | -0,12 | -0,14 | NM_003640 |  |
| RAVER1 | novel_exons | 1010 | -0,12 | -0,10 | NM_133452 |  |
| RAVER1 | novel_exons | 1010 | -0,12 | -0,10 | NM_133452 |  |
| ACINUS | exon(s)skipped | 180 | -0,10 | -0,14 | NM_014977 |  |
| ACINUS | alt_splice_acceptor | 180 | -0,10 | -0,14 | NM_014977 |  |
| BRCA1 | exon(s)skipped | G00033 | -0,10 | -0,11 | NM_007294 |  |
| BRCA1 | exon(s)skipped | G00033 | -0,10 | -0,11 | NM_007294 |  |
| BRCA1 | exon(s)skipped | G00033 | -0,10 | -0,11 | NM_007294 |  |
| BRCA1 | novel_exons | G00033 | -0,10 | -0,11 | NM_007294 |  |
| GRB2 | exon(s)skipped | G00116 | -0,10 | -0,11 | NM_002086 |  |
| HDAC3 | exon(s)skipped | G00118 | -0,10 | -0,07 | NM_003883 |  |
| HDAC3 | exon(s)skipped | G00118 | -0,10 | -0,07 | NM_003883 |  |
| NFKBIA | exon(s)skipped | G00188 | -0,09 | -0,02 | NM_020529 |  |
| NFKBIA | alt_splice_acceptor | G00188 | -0,09 | -0,02 | NM_020529 |  |
| BRIP1 | exon(s)skipped | G00454 | -0,09 | -0,06 | NM_032043 |  |
| MKNK1 | exon(s)skipped | G00171 | -0,09 | -0,07 | NM_003684 |  |
| MKNK1 | novel_exons | G00171 | -0,09 | -0,07 | NM_003684 |  |
| CREBBP | exon(s)skipped | G00387 | -0,08 | -0,08 | NM_004380 |  |
| SPTAN1 | novel_exons | G00246 | -0,07 | -0,04 | NM_003127 |  |
| CLK2 | exon(s)skipped | 70 | -0,06 | -0,02 | NM_003993 |  |
| CLK2 | novel_exons | 70 | -0,06 | -0,02 | NM_003993 |  |
| ENAH | exon(s)skipped | G00101 | -0,05 | -0,05 | NM_001008493 |  |
| ENAH | alt_splice_donor | G00101 | -0,05 | -0,05 | NM_001008493 |  |
| CASP7 | novel_exons | G00047 | -0,04 | 0,00 | NM_001227 |  |
| CASP7 | novel_exons | G00047 | -0,04 | 0,00 | NM_001227 |  |
| MAP3K11 | alt_splice_acceptor | G00146 | -0,04 | -0,01 | NM_002419 |  |
| MAP3K11 | alt_splice_acceptor | G00146 | -0,04 | -0,01 | NM_002419 |  |
| MAP3K11 | novel_exons | G00146 | -0,04 | -0,01 | NM_002419 |  |
| CRADD | novel_exons | G00372 | -0,04 | 0,03 | NM_003805 |  |
| DDX11 | exon(s)skipped | 224 | -0,04 | -0,05 | NM_030653 |  |
| DDX11 | exon(s)skipped | 224 | -0,04 | -0,05 | NM_030653 |  |
| CREB1 | alt_splice_acceptor | G00081 | -0,04 | -0,02 | NM_004379 |  |
| SOS1 | exon(s)skipped | G00245 | -0,04 | -0,04 | NM_005633 |  |
| SOS1 | alt_splice_acceptor | G00245 | -0,04 | -0,04 | NM_005633 |  |
| ATF2 | exon(s)skipped | G00326 | -0,04 | -0,03 | NM_001880 |  |
| Hcc1_(RNPC2) | exon(s)skipped | 258 | -0,03 | -0,06 | NM_004902 |  |
| PDCD4 | alt_splice_acceptor | G00206 | -0,01 | -0,05 | NM_014456 |  |
| AURKB | exon(s)skipped | G00016 | -0,01 | -0,02 | NM_004217 |  |
| AURKB | alt_splice_donor | G00016 | -0,01 | -0,02 | NM_004217 |  |
| AURKB | alt_splice_acceptor | G00016 | -0,01 | -0,02 | NM_004217 |  |
| AURKB | alt_splice_donor | G00016 | -0,01 | -0,02 | NM_004217 |  |
| RB1 | exon(s)skipped | G00293 | -0,01 | -0,02 | NM_000321 |  |
| CASP3 | alt_splice_donor | G00044 | 0,00 | 0,01 | NM_004346 |  |
| MAPK9 | alt_splice_donor | G00160 | 0,00 | 0,03 | NM_002752 |  |
| MAPK9 | exon(s)skipped | G00160 | 0,00 | 0,03 | NM_002752 |  |
| UBE2V1 | exon(s)skipped | G00277 | 0,01 | -0,03 | NM_021988 |  |
| MAP2K5 | exon(s)skipped | G00462 | 0,02 | 0,00 | NM_145160 |  |
| MAP2K5 | exon(s)skipped | G00462 | 0,02 | 0,00 | NM_145160 |  |
| APBB1 | exon(s)skipped | G00007 | 0,02 | 0,04 | NM_001164 |  |
| APBB1 | exon(s)skipped | G00007 | 0,02 | 0,04 | NM_001164 |  |
| NOSIP | alt_splice_acceptor | 186 | 0,02 | -0,02 | NM_015953 |  |
| DFFB | novel_exons | G00090 | 0,02 | 0,04 | NM_001004285 |  |
| RPS6KA4 | exon(s)skipped | G00237 | 0,02 | 0,04 | NM_001006944 |  |
| RIPK1 | novel_exons | G00371 | 0,02 | 0,02 | NM_003804 |  |
| MAPK10 | exon(s)skipped | G00152 | 0,02 | 0,09 | NM_002753 |  |
| heln1_/_ELAVL2 | novel_exons | 76 | 0,03 | 0,04 | NM_004432 |  |
| SAFB | exon(s)skipped | 37 | 0,03 | 0,03 | NM_002967 |  |
| SAFB | exon(s)skipped | 37 | 0,03 | 0,03 | NM_002967 |  |
| BCL2L1 | exon(s)skipped | G00024 | 0,03 | 0,05 | NM_001191 |  |
| MLH3 | novel_exons | G00173 | 0,03 | 0,05 | NM_014381 |  |
| CASP1 | exon(s)skipped | G00041 | 0,03 | 0,04 | NM_001223 |  |
| CASP1 | alt_splice_acceptor | G00041 | 0,03 | 0,04 | NM_001223 |  |
| CASP9 | exon(s)skipped | G00049 | 0,03 | 0,13 | NM_001229 |  |
| FAP | exon(s)skipped | G00390 | 0,03 | 0,06 | NM_004460 |  |
| hnRNP_A1_/_HNRPA1 | exon(s)skipped | 226 | 0,04 | 0,03 | NM_031157 |  |
| SRp40_/_SFRS5 | alt_splice_donor | 144 | 0,04 | 0,06 | NM_006925 |  |
| SRp40_/_SFRS5 | alt_splice_donor | 144 | 0,04 | 0,06 | NM_006925 |  |
| SRp40_/_SFRS5 | alt_splice_donor | 144 | 0,04 | 0,06 | NM_006925 |  |
| SRp40_/_SFRS5 | alt_splice_donor | 144 | 0,04 | 0,06 | NM_006925 |  |
| SRp40_/_SFRS5 | alt_splice_donor | 144 | 0,04 | 0,06 | NM_006925 |  |
| TNFRSF8 | alt_splice_acceptor | G00465 | 0,04 | 0,00 | NM_152942 |  |
| ARHGDIB | alt_splice_donor | G00009 | 0,05 | 0,05 | NM_001175 |  |
| ARHGDIB | alt_splice_acceptor | G00009 | 0,05 | 0,05 | NM_001175 |  |
| PRPF19 | exon(s)skipped | 599_I | 0,05 | 0,04 | NM_014502 |  |
| PRPF19 | exon(s)skipped | 599_I | 0,05 | 0,04 | NM_014502 |  |
| CD44 | exon(s)skipped | G00058 | 0,05 | 0,07 | NM_000610 |  |
| FGFR4 | exon(s)skipped | G00108 | 0,05 | 0,07 | NM_002011 |  |
| UPF2 | exon(s)skipped | 183 | 0,05 | 0,04 | NM_015542 |  |
| SNRPN | novel_exons | 736_I | 0,06 | 0,05 | NM_022806 |  |
| SNRPN | novel_exons | 736_I | 0,06 | 0,05 | NM_022806 |  |
| SNRPN | novel_exons | 736_I | 0,06 | 0,05 | NM_022806 |  |
| ELK1 | novel_exons | G00099 | 0,06 | 0,09 | NM_005229 |  |
| U1-70KD | novel_exons | 40 | 0,06 | -0,04 | NM_003089 |  |
| U1-70KD | novel_exons | 40 | 0,06 | -0,04 | NM_003089 |  |
| AXIN2 | exon(s)skipped | G00018 | 0,06 | 0,06 | NM_004655 |  |
| CUGBP2(BRUNO-L3) | exon(s)skipped | 133 | 0,06 | 0,07 | NM_006561 |  |
| PAK1 | alt_splice_acceptor | G00341 | 0,06 | 0,08 | NM_002576 |  |
| NOVA1 | exon(s)skipped | 28 | 0,07 | 0,08 | NM_002515 |  |
| NOVA1 | alt_splice_acceptor | 28 | 0,07 | 0,08 | NM_002515 |  |
| DUSP1 | exon(s)skipped | G00388 | 0,07 | 0,11 | NM_004417 |  |
| MLLT10 | novel_exons | G00174 | 0,07 | 0,06 | NM_001009569 |  |
| PTPN12 | novel_exons | G00356 | 0,08 | 0,09 | NM_002835 |  |
| CASP8 | exon(s)skipped | G00048 | 0,08 | 0,07 | NM_001228 |  |
| CASP8 | alt_splice_donor | G00048 | 0,08 | 0,07 | NM_001228 |  |
| U2AF1L2 | Mutually-Exclusive-Exons | 832_I | 0,08 | 0,11 | NM_005089 |  |
| ERBB2 | exon(s)skipped | G00389 | 0,08 | 0,13 | NM_004448 |  |
| RPS6KA1 | novel_exons | G00236 | 0,08 | 0,14 | NM_001006665 |  |
| MAP3K9 | exon(s)skipped | G00455 | 0,09 | 0,12 | NM_033141 |  |
| MAP3K9 | exon(s)skipped | G00455 | 0,09 | 0,12 | NM_033141 |  |
| CHUK | exon(s)skipped | G00075 | 0,10 | 0,13 | NM_001279 |  |
| DLEC1 | alt_splice_donor | G00397 | 0,10 | 0,11 | NM_005106 |  |
| PCSK9 | alt_splice_acceptor | G00201 | 0,10 | 0,12 | NM_174936 |  |
| MAP4K2 | exon(s)skipped | G00150 | 0,11 | 0,12 | NM_004579 |  |
| MAP2K3 | novel_exons | G00461 | 0,11 | 0,16 | NM_145109 |  |
| MAP2K3 | alt_splice_acceptor | G00461 | 0,11 | 0,16 | NM_145109 |  |
| ERG | exon(s)skipped | 257 | 0,11 | 0,10 | NM_182918 |  |
| ERG | exon(s)skipped | 257 | 0,11 | 0,10 | NM_182918 |  |
| NFKB1 | exon(s)skipped | G00186 | 0,12 | 0,20 | NM_003998 |  |
| SRPK1 | exon(s)skipped | 753_I | 0,12 | 0,12 | NM_003137 |  |
| MHC2TA | novel_exons | G00170 | 0,12 | 0,17 | NM_000246 |  |
| CD19 | exon(s)skipped | G00056 | 0,13 | 0,18 | NM_001770 |  |
| CD19 | exon(s)skipped | G00056 | 0,13 | 0,18 | NM_001770 |  |
| CD19 | alt_splice_acceptor | G00056 | 0,13 | 0,18 | NM_001770 |  |
| SAD1 | exon(s)skipped | 134 | 0,16 | 0,13 | NM_006590 |  |
| AMID | alt_splice_donor | G00006 | 0,16 | 0,24 | NM_032797 |  |
| WT1 | exon(s)skipped | 222 | 0,18 | 0,22 | NM_024426 |  |
| WT1 | exon(s)skipped | 222 | 0,18 | 0,22 | NM_024426 |  |
| ESR1 | exon(s)skipped | G00103 | 0,19 | 0,20 | NM_000125 |  |
| ESR1 | exon(s)skipped | G00103 | 0,19 | 0,20 | NM_000125 |  |
| CLK3 | novel_exons | 69 | 0,19 | 0,02 | NM_003992 |  |
| CUGBP2 | exon(s)skipped | G00084 | 0,21 | 0,21 | NM_006561 |  |
| CUGBP2 | novel_exons | G00084 | 0,21 | 0,21 | NM_006561 |  |
| QKI | exon(s)skipped | 263 | 0,22 | 0,19 | NM_206853 |  |
| QKI | alt_splice_acceptor | 263 | 0,22 | 0,19 | NM_206853 |  |
| QKI | alt_splice_acceptor | 263 | 0,22 | 0,19 | NM_206853 |  |
| DNMT3B | exon(s)skipped | G00094 | 0,24 | 0,22 | NM_006892 |  |
| DNMT3B | exon(s)skipped | G00094 | 0,24 | 0,22 | NM_006892 |  |
| IL21R | novel_exons | G00130 | 0,25 | 0,28 | NM_021798 |  |
| MBNL2 | exon(s)skipped | 420_I | 0,26 | 0,18 | NM_144778 |  |
| MDM2 | exon(s)skipped | G00168 | 0,27 | 0,24 | NM_002392 |  |
| MDM2 | exon(s)skipped | G00168 | 0,27 | 0,24 | NM_002392 |  |
| MDM2 | exon(s)skipped | G00168 | 0,27 | 0,24 | NM_002392 |  |
| MDM2 | exon(s)skipped | G00168 | 0,27 | 0,24 | NM_002392 |  |
| MDM2 | exon(s)skipped | G00168 | 0,27 | 0,24 | NM_002392 |  |
| MDM2 | exon(s)skipped | G00168 | 0,27 | 0,24 | NM_002392 |  |
| MDM2 | exon(s)skipped | G00168 | 0,27 | 0,24 | NM_002392 |  |
| MDM2 | exon(s)skipped | G00168 | 0,27 | 0,24 | NM_002392 |  |
| MDM2 | exon(s)skipped | G00168 | 0,27 | 0,24 | NM_002392 |  |
| NRAS | exon(s)skipped | G00196 | 0,28 | 0,28 | NM_002524 |  |
| MGC13125 | alt_splice_donor | 237 | 0,28 | 0,28 | NM_032725 |  |
| NUMA | alt_splice_donor | 120 | 0,32 | 0,21 | NM_006185 |  |
| TCL1A | alt_splice_acceptor | G00253 | 0,33 | 0,35 | NM_021966 |  |
| TCL1A | exon(s)skipped | G00253 | 0,33 | 0,35 | NM_021966 |  |
| SmE1_/_SNRPE | alt_splice_donor | 43 | -1,75 | -1,72 | NM_003094 |  |
| U2-A'_/_SNRPA1 | exon(s)skipped | 41 | -1,53 | -1,28 | NM_003090 |  |
| UAP56_(BAT1) | exon(s)skipped | 243 | -1,41 | -1,42 | NM_080598 |  |
| hnRNP_E2_/_PCBP2 | exon(s)skipped | 232 | -1,36 | -1,32 | NM_031989 |  |
| hnRNP_E2_/_PCBP2 | exon(s)skipped | 232 | -1,36 | -1,32 | NM_031989 |  |
| hnRNP_E2_/_PCBP2 | exon(s)skipped | 232 | -1,36 | -1,32 | NM_031989 |  |
| hnRNP_E2_/_PCBP2 | exon(s)skipped | 232 | -1,36 | -1,32 | NM_031989 |  |
| hnRNP_E2_/_PCBP2 | exon(s)skipped | 232 | -1,36 | -1,32 | NM_031989 |  |
| hnRNP_E2_/_PCBP2 | exon(s)skipped | 232 | -1,36 | -1,32 | NM_031989 |  |
| hnRNP_E2_/_PCBP2 | exon(s)skipped | 232 | -1,36 | -1,32 | NM_031989 |  |
| hnRNP_E2_/_PCBP2 | exon(s)skipped | 232 | -1,36 | -1,32 | NM_031989 |  |
| tra2_beta_/_SFRS10 | exon(s)skipped | 79 | -1,25 | -1,19 | NM_004593 |  |
| SRp20_/_SFRS3 | novel_exons | 39 | -1,05 | -1,15 | NM_003017 |  |
| SRp20_/_SFRS3 | novel_exons | 39 | -1,05 | -1,15 | NM_003017 |  |
| NOL5A | novel_exons | 127 | -1,05 | -0,94 | NM_006392 |  |
| NOL5A | novel_exons | 127 | -1,05 | -0,94 | NM_006392 |  |
| NOL5A | novel_exons | 127 | -1,05 | -0,94 | NM_006392 |  |
| NOL5A | alt_splice_acceptor | 127 | -1,05 | -0,94 | NM_006392 |  |
| 9G8_/_SFRS7 | exon(s)skipped | 123 | -0,98 | -1,03 | NM_006276 |  |
| 9G8_/_SFRS7 | exon(s)skipped | 123 | -0,98 | -1,03 | NM_006276 |  |
| 9G8_/_SFRS7 | exon(s)skipped | 123 | -0,98 | -1,03 | NM_006276 |  |
| 9G8_/_SFRS7 | exon(s)skipped | 123 | -0,98 | -1,03 | NM_006276 |  |
| 9G8_/_SFRS7 | exon(s)skipped | 123 | -0,98 | -1,03 | NM_006276 |  |
| 9G8_/_SFRS7 | exon(s)skipped | 123 | -0,98 | -1,03 | NM_006276 |  |
| C1QBP | exon(s)skipped | 73_I | -0,98 | -0,99 | NM_001212 |  |
| ITGB1 | exon(s)skipped | G00331 | -0,97 | -0,81 | NM_002211 |  |
| ITGB1 | exon(s)skipped | G00331 | -0,97 | -0,81 | NM_002211 |  |
| ITGB1 | exon(s)skipped | G00331 | -0,97 | -0,81 | NM_002211 |  |
| NME1 | alt_splice_acceptor | G00190 | -0,89 | -0,85 | NM_000269 |  |
| DFFA | alt_splice_donor | G00089 | -0,89 | -0,89 | NM_004401 |  |
| AATF | alt_splice_donor | G00002 | -0,86 | -0,73 | NM_012138 |  |
| U2AF35_/_U2AF1 | novel_exons | 140 | -0,83 | -0,83 | NM_006758 |  |
| POLB | exon(s)skipped | G00347 | -0,81 | -0,77 | NM_002690 |  |
| POLB | exon(s)skipped | G00347 | -0,81 | -0,77 | NM_002690 |  |
| PABP_4_/_PABPC4 | exon(s)skipped | 62 | -0,79 | -0,77 | NM_003819 |  |
| SFRS2 | alt_splice_donor | 38 | -0,77 | -0,84 | NM_003016 |  |
| SFRS2 | alt_splice_donor | 38 | -0,77 | -0,84 | NM_003016 |  |
| SFRS2 | alt_splice_acceptor | 38 | -0,77 | -0,84 | NM_003016 |  |
| Sam_68_(KHDRBS1)_ | exon(s)skipped | 131 | -0,75 | -0,75 | NM_006559 |  |
| PDCD2 | novel_exons | G00205 | -0,74 | -0,80 | NM_002598 |  |
| PDCD2 | novel_exons | G00205 | -0,74 | -0,80 | NM_002598 |  |
| PDCD10 | exon(s)skipped | G00203 | -0,73 | -0,66 | NM_007217 |  |
| ZNF207 | exon(s)skipped | 50 | -0,66 | -0,77 | NM_003457 |  |
| ZNF207 | exon(s)skipped | 50 | -0,66 | -0,77 | NM_003457 |  |
| PDCD8 | alt_splice_acceptor | G00209 | -0,63 | -0,59 | NM_004208 |  |
| RBMS1 | novel_exons | 648_I | -0,63 | -0,63 | NM_002897 |  |
| PRKDC | exon(s)skipped | G00433 | -0,62 | -0,58 | NM_006904 |  |
| PRKDC | alt_splice_acceptor | G00433 | -0,62 | -0,58 | NM_006904 |  |
| PRKDC | exon(s)skipped | G00433 | -0,62 | -0,58 | NM_006904 |  |
| PRKDC | exon(s)skipped | G00433 | -0,62 | -0,58 | NM_006904 |  |
| PRKDC | exon(s)skipped | G00433 | -0,62 | -0,58 | NM_006904 |  |
| hnRNP_I_(PTB) | exon(s)skipped | 33 | -0,62 | -0,61 | NM_002819 |  |
| FVT1 | exon(s)skipped | G00330 | -0,59 | -0,55 | NM_002035 |  |
| PRPF8 | exon(s)skipped | 604_I | -0,54 | -0,49 | NM_006445 |  |
| SIP1 | exon(s)skipped | 54 | -0,43 | -0,68 | NM_003616 |  |
|  |  |  |  |  |  |  |

| **LAP-35 AS changes** |  |  |  |  |  |  |
| --- | --- | --- | --- | --- | --- | --- |
| **gene name** | **event** | **GeneID** | **GE average** | **GE median** | **Reference RefSeq** |  |
| CDC40 | novel_exons | 116_I | -0,47 | -0,44 | NM_015891 |  |
| hnRNP_R_/_HNRPR | exon(s)skipped | 108 | -0,47 | -0,38 | NM_001102398 |  |
| hnRNP_R_/_HNRPR | exon(s)skipped | 108 | -0,47 | -0,38 | NM_001102398 |  |
| hnRNP_R_/_HNRPR | exon(s)skipped | 108 | -0,47 | -0,38 | NM_001102398 |  |
| hnRNP_R_/_HNRPR | exon(s)skipped | 108 | -0,47 | -0,38 | NM_001102398 |  |
| DEK | exon(s)skipped | 193_I | -0,46 | -0,46 | NM_003472 |  |
| DEK | exon(s)skipped | 193_I | -0,46 | -0,46 | NM_003472 |  |
| DEK | novel_exons | 193_I | -0,46 | -0,46 | NM_003472 |  |
| **EWS** | exon(s)skipped | 1009 | -0,45 | -0,45 | NM_005243 |  |
| **EWS** | novel_exons | 1009 | -0,45 | -0,45 | NM_005243 |  |
| **EWS** | exon(s)skipped | 1009 | -0,45 | -0,45 | NM_005243 |  |
| **EWS** | alt_splice_acceptor | 1009 | -0,45 | -0,45 | NM_005243 |  |
| SR89(LUC7L) | exon(s)skipped | 203 | -0,45 | -0,37 | NM_018032 |  |
| SFRS9 | Mutually-Exclusive-Exons | 700_I | -0,44 | -0,48 | NM_003769 |  |
| RBM15 | exon(s)skipped | 632_I | -0,43 | -0,40 | NM_022768 |  |
| CHEK1 | alt_splice_donor | G00072 | -0,43 | -0,44 | NM_001274 |  |
| STK6 | exon(s)skipped | G00015 | -0,43 | -0,35 | NM_003600 |  |
| STK6 | alt_splice_donor | G00015 | -0,43 | -0,35 | NM_003600 |  |
| STK6 | alt_splice_donor | G00015 | -0,43 | -0,35 | NM_003600 |  |
| STK6 | alt_splice_donor | G00015 | -0,43 | -0,35 | NM_003600 |  |
| p68_/_DDX5 | novel_exons | 74 | -0,43 | -0,40 | NM_004396 |  |
| SWAP_/_SFRS8 | exon(s)skipped | 249 | -0,42 | -0,41 | NM_152235 |  |
| TIAL1 | novel_exons | 789_I | -0,42 | -0,43 | NM_001033925 |  |
| TIAL1 | exon(s)skipped | 789_I | -0,42 | -0,43 | NM_001033925 |  |
| TIAL1 | novel_exons | 789_I | -0,42 | -0,43 | NM_001033925 |  |
| MAP3K7 | novel_exons | G00149 | -0,42 | -0,46 | NM_003188 |  |
| SFRS2 | alt_splice_donor | 38 | -0,41 | -0,41 | NM_003016 |  |
| SFRS2 | alt_splice_acceptor | 38 | -0,41 | -0,41 | NM_003016 |  |
| SFRS2 | intron_retained | 38 | -0,41 | -0,41 | NM_003016 |  |
| SFRS2 | alt_splice_donor | 38 | -0,41 | -0,41 | NM_003016 |  |
| SFRS2 | alt_splice_acceptor | 38 | -0,41 | -0,41 | NM_003016 |  |
| SFRS2 | novel_exons | 38 | -0,41 | -0,41 | NM_003016 |  |
| SmE1_/_SNRPE | alt_splice_donor | 43 | -0,41 | -0,44 | NM_003094 |  |
| SmE1_/_SNRPE | alt_splice_donor | 43 | -0,41 | -0,44 | NM_003094 |  |
| TNFRSF10B | exon(s)skipped | G00263 | -0,40 | -0,38 | NM_003842 |  |
| AATF | alt_splice_donor | G00002 | -0,39 | -0,35 | NM_012138 |  |
| AATF | alt_splice_acceptor | G00002 | -0,39 | -0,35 | NM_012138 |  |
| hnRNP_M | novel_exons | 114 | -0,38 | -0,27 | NM_005968 |  |
| RAF1 | exon(s)skipped | G00359 | -0,37 | -0,33 | NM_002880 |  |
| RAF1 | novel_exons | G00359 | -0,37 | -0,33 | NM_002880 |  |
| PLK1 | alt_splice_donor | G00214 | -0,36 | -0,34 | NM_005030 |  |
| GRB2 | exon(s)skipped | G00116 | -0,36 | -0,38 | NM_002086 |  |
| PPIL3b_/_PPIL3 | exon(s)skipped | 236 | -0,35 | -0,32 | NM_032472 |  |
| RAD51 | exon(s)skipped | G00227 | -0,34 | -0,31 | NM_002875 |  |
| SF3B3 | exon(s)skipped | 681_I | -0,33 | -0,30 | NM_012426 |  |
| SF3B3 | Mutually-Exclusive-Exons | 681_I | -0,33 | -0,30 | NM_012426 |  |
| RNPS1 | exon(s)skipped | 242 | -0,32 | -0,32 | NM_080594 |  |
| RNPS1 | novel_exons | 242 | -0,32 | -0,32 | NM_080594 |  |
| RNPS1 | novel_exons | 242 | -0,32 | -0,32 | NM_080594 |  |
| RNPS1 | novel_exons | 242 | -0,32 | -0,32 | NM_080594 |  |
| FUBP1 | novel_exons | 281_I | -0,32 | -0,19 | NM_003902 |  |
| ITGB1 | exon(s)skipped | G00331 | -0,31 | -0,26 | NM_002211 |  |
| ITGB1 | alt_splice_donor | G00331 | -0,31 | -0,26 | NM_002211 |  |
| WDR57 | Mutually-Exclusive-Exons | 858_I | -0,29 | -0,31 | NM_004814 |  |
| hnRNP_E2_/_PCBP2 | alt_splice_acceptor | 232 | -0,28 | -0,26 | NM_031989 |  |
| MAP2K4 | exon(s)skipped | G00144 | -0,26 | -0,24 | NM_003010 |  |
| RAMP | exon(s)skipped | G00230 | -0,25 | -0,22 | NM_016448 |  |
| MAPK14 | novel_exons | G00155 | -0,24 | -0,28 | NM_001315 |  |
| MAPK14 | novel_exons | G00155 | -0,24 | -0,28 | NM_001315 |  |
| SF3A3 | exon(s)skipped | 677_I | -0,24 | -0,26 | NM_006802 |  |
| SmB/B'_/_SNRPB | novel_exons | 259 | -0,23 | -0,20 | NM_198216 |  |
| PDCD2 | novel_exons | G00205 | -0,23 | -0,24 | NM_002598 |  |
| BLM | exon(s)skipped | G00284 | -0,22 | -0,20 | NM_000057 |  |
| 15.5_tri-snRNP_/_NHP2L1 | novel_exons | 96 | -0,22 | -0,19 | NM_005008 |  |
| 15.5_tri-snRNP_/_NHP2L1 | exon(s)skipped | 96 | -0,22 | -0,19 | NM_005008 |  |
| 15.5_tri-snRNP_/_NHP2L1 | exon(s)skipped | 96 | -0,22 | -0,19 | NM_005008 |  |
| PTPN12 | exon(s)skipped | G00356 | -0,20 | -0,17 | NM_002835 |  |
| SLM2_/KHDRBS3 | exon(s)skipped | 130 | -0,20 | -0,13 | NM_006558 |  |
| C1QBP | novel_exons | 73_I | -0,17 | -0,17 | NM_001212 |  |
| RB1CC1 | alt_splice_acceptor | G00231 | -0,17 | -0,21 | NM_014781 |  |
| ELAC2 | exon(s)skipped | G00097 | -0,16 | -0,13 | NM_018127 |  |
| PUF60 | novel_exons | 241 | -0,15 | -0,14 | NM_078480 |  |
| CROP | novel_exons | 165_I | -0,15 | -0,12 | NM_006107 |  |
| CROP | Mutually-Exclusive-Exons | 165_I | -0,15 | -0,12 | NM_006107 |  |
| UAP56_(BAT1) | novel_exons | 243 | -0,08 | -0,07 | NM_080598 |  |
| UAP56_(BAT1) | novel_exons | 243 | -0,08 | -0,07 | NM_080598 |  |
| AURKB | exon(s)skipped | G00016 | -0,03 | -0,01 | NM_004217 |  |
| AURKB | alt_splice_donor | G00016 | -0,03 | -0,01 | NM_004217 |  |
| FN1 | novel_exons | 274_I | 0,03 | 0,03 | NM_212482 |  |
| NOL5A | novel_exons | 127 | 0,05 | 0,02 | NM_006392 |  |
| NOL5A | novel_exons | 127 | 0,05 | 0,02 | NM_006392 |  |
| NOL5A | novel_exons | 127 | 0,05 | 0,02 | NM_006392 |  |
| SAD1 | exon(s)skipped | 134 | 0,06 | 0,03 | NM_006590 |  |
| ATM | exon(s)skipped | G00460 | 0,06 | 0,09 | NM_138292 |  |
| SPTAN1 | novel_exons | G00246 | 0,07 | 0,08 | NM_003127 |  |
| FRG1 | novel_exons | 77 | 0,12 | 0,12 | NM_004477 |  |
| TCL1A | alt_splice_acceptor | G00253 | 0,16 | 0,16 | NM_021966 |  |
| TCL1A | exon(s)skipped | G00253 | 0,16 | 0,16 | NM_021966 |  |
| PAX5 | exon(s)skipped | G00199 | 0,17 | 0,17 | NM_016734 |  |
| TFE3(CA150) | novel_exons | 139 | 0,18 | 0,11 | NM_006706 |  |
| SmD1_/_SNRPD1 | alt_splice_acceptor | 146 | -1,00 | -1,00 | NM_006938 |  |
| tra2_beta_/_SFRS10 | novel_exons | 79 | -0,83 | -0,74 | NM_004593 |  |
| tra2_beta_/_SFRS10 | alt_splice_donor | 79 | -0,83 | -0,74 | NM_004593 |  |
| SRp20_/_SFRS3 | novel_exons | 39 | -0,81 | -0,73 | NM_003017 |  |
| SRp20_/_SFRS3 | novel_exons | 39 | -0,81 | -0,73 | NM_003017 |  |
| 9G8_/_SFRS7 | exon(s)skipped | 123 | -0,79 | -0,79 | NM_006276 |  |
| 9G8_/_SFRS7 | exon(s)skipped | 123 | -0,79 | -0,79 | NM_006276 |  |
| 9G8_/_SFRS7 | exon(s)skipped | 123 | -0,79 | -0,79 | NM_006276 |  |
| 9G8_/_SFRS7 | exon(s)skipped | 123 | -0,79 | -0,79 | NM_006276 |  |
| CCNA2 | exon(s)skipped | G00313 | -0,73 | -0,70 | NM_001237 |  |
| AP5GL | alt_splice_acceptor | G00008 | -0,68 | -0,51 | NM_004849 |  |
| SIP1 | exon(s)skipped | 54 | -0,58 | -0,59 | NM_003616 |  |
| U2AF35_/_U2AF1 | novel_exons | 140 | -0,57 | -0,57 | NM_006758 |  |
| U2AF35_/_U2AF1 | novel_exons | 140 | -0,57 | -0,57 | NM_006758 |  |
| BUB3 | alt_splice_acceptor | 87 | -0,52 | -0,50 | NM_004725 |  |
| ZNF207 | exon(s)skipped | 50 | -0,50 | -0,55 | NM_003457 |  |
| ZNF207 | exon(s)skipped | 50 | -0,50 | -0,55 | NM_003457 |  |
| FOS | exon(s)skipped | G00111 | 0,71 | 0,71 | NM_005252 |  |
|  |  |  |  |  |  |  |

| **HEP3B AS changes** |  |  |  |  |  |  |
| --- | --- | --- | --- | --- | --- | --- |
|  |  |  | **GE** | |  |  |
| **gene name** | **event** | **GeneID** | **GE average** | **GE median** | **Reference RefSeq** |  |
| BCL9 | novel_exons | G00028 | -0,0585 | -0,0533 | NM_004326 |  |
| TRIB3 | novel_exons | G00037 | -0,4801 | -0,4779 | NM_021158 |  |
| TRIB3 | novel_exons | G00037 | -0,4801 | -0,4779 | NM_021158 |  |
| CDC25A | exon(s)skipped | G00062 | -0,2493 | -0,2879 | NM_001789 |  |
| CHEK1 | alt_splice_donor | G00072 | -0,4335 | -0,3838 | NM_001274 |  |
| DIABLO | novel_exons | G00092 | -0,1831 | -0,2029 | NM_019887 |  |
| DNCH1 | exon(s)skipped | G00093 | 0,3506 | 0,3049 | NM_001376 |  |
| E2F4 | alt_splice_donor | G00096 | -0,3481 | -0,2896 | NM_001950 |  |
| E2F4 | exon(s)skipped | G00096 | -0,3481 | -0,2896 | NM_001950 |  |
| FOS | exon(s)skipped | G00111 | 0,1137 | 0,1233 | NM_005252 |  |
| MAPKAPK5 | novel_exons | G00163 | -0,3417 | -0,2895 | NM_003668 |  |
| MKNK2 | novel_exons | G00172 | -0,3636 | -0,3148 | NM_017572 |  |
| MKNK2 | alt_splice_donor | G00172 | -0,3636 | -0,3148 | NM_017572 |  |
| PDCD6IP | novel_exons | G00208 | -0,1537 | -0,1477 | NM_013374 |  |
| PMS1 | exon(s)skipped | G00217 | -0,0748 | -0,1025 | NM_000534 |  |
| RB1CC1 | alt_splice_acceptor | G00231 | -0,2896 | -0,319 | NM_014781 |  |
| SPTAN1 | novel_exons | G00246 | 0,0876 | 0,0535 | NM_003127 |  |
| TCL1A | alt_splice_acceptor | G00253 | 0,169 | 0,1065 | NM_021966 |  |
| TCL1A | exon(s)skipped | G00253 | 0,169 | 0,1065 | NM_021966 |  |
| TNFRSF10B | exon(s)skipped | G00263 | -0,2494 | -0,2552 | NM_003842 |  |
| TNFRSF1B | alt_splice_acceptor | G00265 | 0,0331 | 0,0507 | NM_001066 |  |
| VEGF | alt_splice_donor | G00278 | -0,081 | -0,0834 | NM_003376 |  |
| VEGF | exon(s)skipped | G00278 | -0,081 | -0,0834 | NM_003376 |  |
| ZNF198 | exon(s)skipped | G00279 | -0,3692 | -0,3561 | NM_003453 |  |
| MALT1 | novel_exons | G00430 | -0,4478 | -0,4266 | NM_006785 |  |
| MALT1 | exon(s)skipped | G00430 | -0,4478 | -0,4266 | NM_006785 |  |
| PRKDC | exon(s)skipped | G00433 | 0,1563 | 0,075 | NM_006904 |  |
| PRKDC | exon(s)skipped | G00433 | 0,1563 | 0,075 | NM_006904 |  |
| PRKDC | alt_splice_donor | G00433 | 0,1563 | 0,075 | NM_006904 |  |
| PRKDC | exon(s)skipped | G00433 | 0,1563 | 0,075 | NM_006904 |  |
| BRIP1 | exon(s)skipped | G00454 | -0,4051 | -0,3652 | NM_032043 |  |
| ATM | exon(s)skipped | G00460 | -0,0484 | -0,0469 | NM_138292 |  |
| MAP2K7 | novel_exons | G00463 | -0,2077 | -0,2039 | NM_145185 |  |
| EWS | novel_exons | 1009 | 0,1115 | 0,1141 | NM_005243 |  |
| CDK9 | exon(s)skipped | 1015 | -0,2252 | -0,2195 | NM_001261 |  |
| CDK9 | alt_splice_acceptor | 1015 | -0,2252 | -0,2195 | NM_001261 |  |
| NOVA1 | alt_splice_acceptor | 28 | 0,0349 | 0,0314 | NM_002515 |  |
| SFRS2 | alt_splice_acceptor | 38 | -0,1839 | -0,1871 | NM_003016 |  |
| SFRS2 | intron_retained | 38 | -0,1839 | -0,1871 | NM_003016 |  |
| SFRS2 | alt_splice_acceptor | 38 | -0,1839 | -0,1871 | NM_003016 |  |
| SFRS2 | novel_exons | 38 | -0,1839 | -0,1871 | NM_003016 |  |
| SIP1 | novel_exons | 54 | -0,465 | -0,412 | NM_003616 |  |
| SRp54(SFRS11) | exon(s)skipped | 88 | -0,4559 | -0,4538 | NM_004768 |  |
| SRp54(SFRS11) | novel_exons | 88 | -0,4559 | -0,4538 | NM_004768 |  |
| SRp55_/_SFRS6 | novel_exons | 122 | 0,073 | 0,0993 | NM_006275 |  |
| 9G8_/_SFRS7 | exon(s)skipped | 123 | -0,4695 | -0,4729 | NM_006276 |  |
| 9G8_/_SFRS7 | exon(s)skipped | 123 | -0,4695 | -0,4729 | NM_006276 |  |
| 9G8_/_SFRS7 | exon(s)skipped | 123 | -0,4695 | -0,4729 | NM_006276 |  |
| 9G8_/_SFRS7 | exon(s)skipped | 123 | -0,4695 | -0,4729 | NM_006276 |  |
| NOL5A | novel_exons | 127 | -0,1639 | -0,1805 | NM_006392 |  |
| NOL5A | novel_exons | 127 | -0,1639 | -0,1805 | NM_006392 |  |
| CUG-BP | novel_exons | 132 | -0,2914 | -0,2912 | NM_006560 |  |
| CUG-BP | novel_exons | 132 | -0,2914 | -0,2912 | NM_006560 |  |
| CUG-BP | novel_exons | 132 | -0,2914 | -0,2912 | NM_006560 |  |
| CUG-BP | novel_exons | 132 | -0,2914 | -0,2912 | NM_006560 |  |
| CUG-BP | novel_exons | 132 | -0,2914 | -0,2912 | NM_006560 |  |
| U2AF35_/_U2AF1 | novel_exons | 140 | -0,1573 | -0,1517 | NM_006758 |  |
| U2AF35_/_U2AF1 | novel_exons | 140 | -0,1573 | -0,1517 | NM_006758 |  |
| SmD1_/_SNRPD1 | alt_splice_acceptor | 146 | -0,2659 | -0,2648 | NM_006938 |  |
| LSM1 | exon(s)skipped | 174 | -0,3984 | -0,4026 | NM_014462 |  |
| MGC13125 | alt_splice_donor | 237 | -0,1208 | -0,1008 | NM_032725 |  |
| SPF45 | novel_exons | 238 | -0,4762 | -0,4703 | NM_032905 |  |
| RNPS1 | novel_exons | 242 | -0,3267 | -0,3127 | NM_080594 |  |
| RNPS1 | novel_exons | 242 | -0,3267 | -0,3127 | NM_080594 |  |
| RNPS1 | novel_exons | 242 | -0,3267 | -0,3127 | NM_080594 |  |
| RNPS1 | exon(s)skipped | 242 | -0,3267 | -0,3127 | NM_080594 |  |
| RNPS1 | alt_splice_donor | 242 | -0,3267 | -0,3127 | NM_080594 |  |
| UAP56_(BAT1) | novel_exons | 243 | 0,0902 | 0,0833 | NM_080598 |  |
| UAP56_(BAT1) | novel_exons | 243 | 0,0902 | 0,0833 | NM_080598 |  |
| SFRS12 | exon(s)skipped | 246 | -0,3321 | -0,324 | NM_139168 |  |
| Hcc1_(RNPC2) | exon(s)skipped | 258 | -0,4542 | -0,4649 | NM_004902 |  |
| Hcc1_(RNPC2) | novel_exons | 258 | -0,4542 | -0,4649 | NM_004902 |  |
| QKI | alt_splice_acceptor | 263 | 0,0542 | 0,016 | NM_206853 |  |
| QKI | alt_splice_donor | 263 | 0,0542 | 0,016 | NM_206853 |  |
| CDC40 | novel_exons | 116_I | -0,4244 | -0,4047 | NM_015891 |  |
| DEK | exon(s)skipped | 193_I | 0,0971 | 0,0948 | NM_003472 |  |
| DHX9 | novel_exons | 202_I | -0,052 | -0,11 | NM_001357 |  |
| MBNL1 | Mutually-Exclusive-Exons | 419_I | -0,486 | -0,4845 | NM_021038 |  |
| MBNL1 | novel_exons | 419_I | -0,486 | -0,4845 | NM_021038 |  |
| MBNL1 | Mutually-Exclusive-Exons | 419_I | -0,486 | -0,4845 | NM_021038 |  |
| MBNL1 | novel_exons | 419_I | -0,486 | -0,4845 | NM_021038 |  |
| C1QBP | novel_exons | 73_I | 0,0468 | 0,0574 | NM_001212 |  |
| THOC1 | novel_exons | 784_I | -0,4196 | -0,3379 | NM_005131 |  |
| AP5GL | alt_splice_acceptor | G00008 | -1,0818 | -0,8767 | NM_004849 |  |
| AP5GL | exon(s)skipped | G00008 | -1,0818 | -0,8767 | NM_004849 |  |
| AP5GL | exon(s)skipped | G00008 | -1,0818 | -0,8767 | NM_004849 |  |
| STK6 | novel_exons | G00015 | -1,4179 | -1,3543 | NM_003600 |  |
| STK6 | alt_splice_donor | G00015 | -1,4179 | -1,3543 | NM_003600 |  |
| STK6 | alt_splice_donor | G00015 | -1,4179 | -1,3543 | NM_003600 |  |
| STK6 | alt_splice_donor | G00015 | -1,4179 | -1,3543 | NM_003600 |  |
| STK6 | exon(s)skipped | G00015 | -1,4179 | -1,3543 | NM_003600 |  |
| STK6 | novel_exons | G00015 | -1,4179 | -1,3543 | NM_003600 |  |
| BCL2L1 | novel_exons | G00024 | -0,5297 | -0,5416 | NM_001191 |  |
| BCL2L1 | alt_splice_donor | G00024 | -0,5297 | -0,5416 | NM_001191 |  |
| CDC2 | alt_splice_donor | G00061 | -1,0178 | -0,9851 | NM_001786 |  |
| CREB1 | exon(s)skipped | G00081 | -0,6416 | -0,6075 | NM_004379 |  |
| CREB1 | alt_splice_donor | G00081 | -0,6416 | -0,6075 | NM_004379 |  |
| CREB1 | alt_splice_acceptor | G00081 | -0,6416 | -0,6075 | NM_004379 |  |
| CREB1 | novel_exons | G00081 | -0,6416 | -0,6075 | NM_004379 |  |
| GADD45A | exon(s)skipped | G00114 | 0,5347 | 0,5342 | NM_001924 |  |
| MAP2K4 | exon(s)skipped | G00144 | -0,7358 | -0,7012 | NM_003010 |  |
| MAP2K4 | exon(s)skipped | G00144 | -0,7358 | -0,7012 | NM_003010 |  |
| MAPK14 | novel_exons | G00155 | -0,5262 | -0,4587 | NM_001315 |  |
| MAPK14 | novel_exons | G00155 | -0,5262 | -0,4587 | NM_001315 |  |
| MCL1 | exon(s)skipped | G00166 | -1,2846 | -1,2846 | NM_021960 |  |
| NFKB1 | exon(s)skipped | G00186 | -0,661 | -0,7054 | NM_003998 |  |
| NFKB1 | alt_splice_acceptor | G00186 | -0,661 | -0,7054 | NM_003998 |  |
| NFKBIA | alt_splice_acceptor | G00188 | -0,9937 | -0,9673 | NM_020529 |  |
| NFKBIA | exon(s)skipped | G00188 | -0,9937 | -0,9673 | NM_020529 |  |
| NFKBIA | exon(s)skipped | G00188 | -0,9937 | -0,9673 | NM_020529 |  |
| PDCD10 | alt_splice_donor | G00203 | -1,2023 | -1,1324 | NM_007217 |  |
| PDCD10 | exon(s)skipped | G00203 | -1,2023 | -1,1324 | NM_007217 |  |
| PDCD10 | alt_splice_acceptor | G00203 | -1,2023 | -1,1324 | NM_007217 |  |
| PDCD10 | exon(s)skipped | G00203 | -1,2023 | -1,1324 | NM_007217 |  |
| PPM1D | novel_exons | G00220 | -0,6274 | -0,6779 | NM_003620 |  |
| PPM1D | novel_exons | G00220 | -0,6274 | -0,6779 | NM_003620 |  |
| SOS1 | exon(s)skipped | G00245 | -1,0777 | -0,9382 | NM_005633 |  |
| SOS1 | exon(s)skipped | G00245 | -1,0777 | -0,9382 | NM_005633 |  |
| SOS1 | novel_exons | G00245 | -1,0777 | -0,9382 | NM_005633 |  |
| SOS1 | exon(s)skipped | G00245 | -1,0777 | -0,9382 | NM_005633 |  |
| SOS1 | alt_splice_acceptor | G00245 | -1,0777 | -0,9382 | NM_005633 |  |
| SOS1 | exon(s)skipped | G00245 | -1,0777 | -0,9382 | NM_005633 |  |
| SOS1 | exon(s)skipped | G00245 | -1,0777 | -0,9382 | NM_005633 |  |
| PTEN | alt_splice_acceptor | G00292 | -0,623 | -0,5553 | NM_000314 |  |
| BUB1B | alt_splice_acceptor | G00312 | -0,5927 | -0,5566 | NM_001211 |  |
| BUB1B | exon(s)skipped | G00312 | -0,5927 | -0,5566 | NM_001211 |  |
| MYC | alt_splice_acceptor | G00335 | -0,777 | -0,7676 | NM_002467 |  |
| PTPN12 | exon(s)skipped | G00356 | -0,7551 | -0,7456 | NM_002835 |  |
| PTPN12 | exon(s)skipped | G00356 | -0,7551 | -0,7456 | NM_002835 |  |
| PTPN12 | novel_exons | G00356 | -0,7551 | -0,7456 | NM_002835 |  |
| PTPN12 | exon(s)skipped | G00356 | -0,7551 | -0,7456 | NM_002835 |  |
| TTK | alt_splice_acceptor | G00365 | -0,7861 | -0,7617 | NM_003318 |  |
| MAP4K3 | exon(s)skipped | G00368 | -0,7152 | -0,7116 | NM_003618 |  |
| MAP4K3 | exon(s)skipped | G00368 | -0,7152 | -0,7116 | NM_003618 |  |
| MAP4K3 | exon(s)skipped | G00368 | -0,7152 | -0,7116 | NM_003618 |  |
| MAP4K3 | exon(s)skipped | G00368 | -0,7152 | -0,7116 | NM_003618 |  |
| MAP4K3 | exon(s)skipped | G00368 | -0,7152 | -0,7116 | NM_003618 |  |
| MAP4K3 | exon(s)skipped | G00368 | -0,7152 | -0,7116 | NM_003618 |  |
| MAP4K3 | exon(s)skipped | G00368 | -0,7152 | -0,7116 | NM_003618 |  |
| CCNC | novel_exons | G00398 | -0,4841 | -0,5074 | NM_005190 |  |
| CCNC | novel_exons | G00398 | -0,4841 | -0,5074 | NM_005190 |  |
| PIK3CA | exon(s)skipped | G00423 | -0,6435 | -0,6324 | NM_006218 |  |
| PLK2 | exon(s)skipped | G00427 | -0,9962 | -0,8309 | NM_006622 |  |
| PRP43_/_DDX15 | exon(s)skipped | 10 | -0,9529 | -0,937 | NM_001358 |  |
| DYRK1A | exon(s)skipped | 11 | -0,9343 | -0,9314 | NM_001396 |  |
| DYRK1A | alt_splice_donor | 11 | -0,9343 | -0,9314 | NM_001396 |  |
| DYRK1A | alt_splice_donor | 11 | -0,9343 | -0,9314 | NM_001396 |  |
| DYRK1A | exon(s)skipped | 11 | -0,9343 | -0,9314 | NM_001396 |  |
| DYRK1A | novel_exons | 11 | -0,9343 | -0,9314 | NM_001396 |  |
| PPP1R8 | novel_exons | 32 | -0,5921 | -0,5079 | NM_002713 |  |
| ZNF207 | alt_splice_donor | 50 | -0,6208 | -0,6387 | NM_003457 |  |
| ZNF207 | exon(s)skipped | 50 | -0,6208 | -0,6387 | NM_003457 |  |
| ZNF207 | alt_splice_donor | 50 | -0,6208 | -0,6387 | NM_003457 |  |
| DUSP11 | exon(s)skipped | 53 | -0,7999 | -0,7943 | NM_003584 |  |
| CLK1 | exon(s)skipped | 71 | -0,554 | -0,5285 | NM_004071 |  |
| CLK1 | intron_retained | 71 | -0,554 | -0,5285 | NM_004071 |  |
| CLK1 | exon(s)skipped | 71 | -0,554 | -0,5285 | NM_004071 |  |
| p68_/_DDX5 | novel_exons | 74 | -0,699 | -0,6305 | NM_004396 |  |
| RBP_7_/_RBM7 | exon(s)skipped | 188 | -1,2407 | -1,1687 | NM_016090 |  |
| SR89(LUC7L) | exon(s)skipped | 203 | -0,6449 | -0,5841 | NM_018032 |  |
| hnRNP_DL__/_HNRPDL | exon(s)skipped | 231 | -0,5263 | -0,5183 | NM_031372 |  |
| CROP | novel_exons | 165_I | -0,6428 | -0,6236 | NM_006107 |  |
| CROP | Mutually-Exclusive-Exons | 165_I | -0,6428 | -0,6236 | NM_006107 |  |
| CROP | novel_exons | 165_I | -0,6428 | -0,6236 | NM_006107 |  |
| LSM8 | novel_exons | 410_I | -0,9408 | -0,9374 | NM_016200 |  |
| MBNL2 | exon(s)skipped | 420_I | -0,5839 | -0,5519 | NM_144778 |  |
| RBM15 | exon(s)skipped | 632_I | -1,5102 | -1,3759 | NM_022768 |  |
| RBMS1 | novel_exons | 648_I | -0,5627 | -0,5313 | NM_002897 |  |
| TIAL1 | novel_exons | 789_I | -0,6094 | -0,601 | NM_001033925 |  |
| TIAL1 | alt_splice_acceptor | 789_I | -0,6094 | -0,601 | NM_001033925 |  |
|  |  |  |  |  |  |  |
